# Computational analysis of 5-fluorouracil anti-tumor activity in colon cancer using a mechanistic pharmacokinetic/pharmacodynamic model

**DOI:** 10.1371/journal.pcbi.1010685

**Published:** 2022-11-17

**Authors:** Chenhui Ma, Alex Almasan, Evren Gurkan-Cavusoglu

**Affiliations:** 1 Department of Electrical, Computer and Systems Engineering, Case Western Reserve University, Cleveland, Ohio, United States of America; 2 Department of Cancer Biology, Lerner Research Institute, Cleveland Clinic, Cleveland, Ohio, United States of America; 3 Case Comprehensive Cancer Center, Case Western Reserve University, Cleveland, Ohio, United States of America; University at Buffalo - The State University of New York, UNITED STATES

## Abstract

5-Fluorouracil (5-FU) is a standard chemotherapeutic agent to treat solid cancers such as breast, colon, head, and neck. Computational modeling plays an essential role in predicting the outcome of chemotherapy and developing optimal dosing strategies. We developed an integrated mechanistic pharmacokinetics/pharmacodynamics (PK/PD) model examining the influence of 5-FU, as an S-phase specific double-strand break (DSB)-inducing agent, on tumor proliferation. The proposed mechanistic PK/PD model simulates the dynamics of critical intermediate components and provides the accurate tumor response prediction. The integrated model is composed of PK, cellular, and tumor growth inhibition (TGI) sub-models, quantitatively capturing the essential drug-related physiological processes. In the cellular model, thymidylate synthase (TS) inhibition, resultant deoxynucleoside triphosphate (dNTP) pool imbalance, and DSB induction are considered, as well as 5-FU incorporation into RNA and DNA. The amount of 5-FU anabolites and DSBs were modeled to drive the kinetics of the pharmacological tumor response. Model parameters were estimated by fitting to literature data. Our simulation results successfully describe the kinetics of the intermediates regulating the 5-FU cytotoxic events and the pattern of tumor suppression. The comprehensive model simulated the tumor volume change under various dose regimens, and its generalizability was attested by comparing it with literature data. The potential causes of the tumor resistance to 5-FU are also investigated through Monte Carlo analysis. The simulation of various dosage regimens helps quantify the relationship between treatment protocols and chemotherapy potency, which will lead to the development of efficacy optimization.

## Author summary

Computational modeling plays an essential role in predicting the outcome of chemotherapy and developing optimal dosing strategies. In this study, we developed a computational model examining the influence of a chemotherapeutic agent, 5-fluorouracil, on tumor proliferation. 5-fluorouracil is a standard chemotherapeutic agent used to treat solid cancers such as breast, colon, head, and neck. Our model traces the dynamics of critical intermediate components and generates the accurate tumor response prediction in a physiologically-based manner for colon cancer. The comprehensive model simulates the tumor volume change under various dosage regimens, and its generalizability is attested by the validation against literature data. We used the models to investigate potential causes of the tumor resistance to 5-fluorouracil. Our model simulations for various dosage regimens help quantify the relationship between treatment protocols and chemotherapy potency.

## Introduction

In this study, we developed a novel mechanistic PK/PD model that integrates a detailed cellular model with the classical PK/PD models to analyze the treatment outcome of the chemotherapeutic agent 5-fluorouracil (5-FU) in colon cancer. The modeling framework enables the quantitative analysis of the drug metabolism together with the downstream antiproliferative events by providing process models for the distribution of 5-FU from its entry into the body to interstitial fluid outside the tumor cells and to its cellular uptake. In the cell model, the connection of cellular level molecular events that lead to tumor suppression are modeled in detail. Over the past decades, pharmacokinetics/pharmacodynamics (PK/PD) models have been widely used in predicting therapeutic outcomes through the evaluation of the causal concentration-effect relationships [1]. Specifically, the PK model concentrates on drug disposition in plasma and/or tissues and describes drug exposure stages. In contrast, the PD model examines the pharmaceutical responses (e.g., tumor volume, white blood cell counts) following the drug treatment. The structures of the PK models differ based on the type of the drug, route of administration (i.e., oral absorption, intravenous injection, intravenous infusion), as well as the availability of clinical data. Commonly used compartment PK models in the literature are one compartment, two-compartment and three-compartment models. The one-compartment PK model considers the drug distribution in the whole organism as homogeneous [2]. The two-compartment PK model represents the transfer of substances between central (richly perfused organs) and peripheral regions (poorly perfused organs) of the human body [3, 4]. One central and two peripheral compartments constitute three-compartment PK models [5, 6]. The multicompartment pharmacokinetics models are preferred over the one-compartment model, because they can accurately represent the plasma concentration-time profile [7]. The pharmacokinetic profile in the central or peripheral compartment is the input of the PD model. The drug transport across the cell membrane is modeled as the irreversible or reversible exchange with the tumor compartment [8]. The PD model for tumor response comprises the kinetics of the control group and the treated group. The population tumor growth kinetics in the absence of the drug intervention is described by ordinary differential equations, including a growth term and a death term. Exponential, Mendelsohn, linear, logistic, Gompertz, and Bertalanffy are the common options for simulating the normal tumor growth patterns [9]. Specifically, exponential, Mendelsohn, linear, and surface growth terms predict that tumors will continue growing without an upper bound. Logistic, Gompertz, and Bertalanffy models predict that tumors will grow to some maximum size and reach a stable equilibrium at that point, based on the finding that the tumor growth is subject to the limited nutrition supply and extracellular cues [9, 10]. Under the effect of tumor therapy, the tumor growth is altered and deviated from its baseline level. As a key player in PD model, the exposure-effect relationship(*E*_*drug*_) driving the drug-induced tumor kinetics is modeled as a function of drug concentration, which can be linear, log-linear and sigmoid [11, 12]. The involvement of drug effect in tumor growth dynamics can be modeled in various ways, depending on the biological question researchers propose to address, the assumptions on which the model construction is based, and the availability of experimental data. In general, the pharmacodynamic models take one or more of the forms of the hypothetical effect compartment model, indirect response model and signal transduction model, irreversible effect model, and complex mechanism-based model [13]. The interpretations of these PD models are provided as follows. The hypothetical effect compartment reproduces the true site of drug action and is applied between the PK and PD model only when the slow onset of observed effects can be explained by the rate-limiting drug distribution to the target site [14]. The indirect response model captures the nonlinear exposure-effect relationship and reflects how the reversible drug action alters the turnover of the drug response variables. Specifically, the sigmoid *E*_max,*damage*_ can be added to the production and loss term to represent either inhibitory or stimulatory effects of drug disposition on the production or the loss of the responses [15, 16]. The PD model encompassing the signaling pathways incorporates the time-dependent intermediate steps connecting the drug-receptor complex and pharmacodynamic response. The time elapsed from drug binding to the onset of the measurable responses is modeled by a transit compartment approach [17, 18]. The irreversible effect model is similar to the indirect response model in certain contexts where the tumor growth is incorporated. But this type of PD model is only applicable to the compounds with irreversible anti-tumor effects and does not alter the turnover rate of a biomarker. The mechanism-based pharmacodynamic models extend from the indirect response model and consider the multiple drug-related mechanistic events represented by the ordinary or partial differential equations defined for the model variables [19]. The underlying physiological processes also warrant the additional modeling components to capture the biological phenomenon.

In recent years, researchers have shown great interest in employing PK/PD models for the prediction of tumor responses to 5-FU. Several conventional PK/PD models in the context of 5-FU treatment have been proposed, in which the transit compartment model is employed to describe the progressive effect on damaged tumor cells [20–22]. Although researchers have gained desirable analysis outcomes through the PK/PD models, the physiological effects under tumor treatment are barely captured. To delve into the complex cellular responses towards chemotherapy and to realize precise prediction, the mechanistic details need to be incorporated in the model design phase. In the literature, semi-mechanistic PK/PD models have been built for 5-FU in recent years, which employ both biological complexity and predictive power of a typical empirical model. In the model developed by Arshad et al. [23], three transit compartments describing blood cell maturation were used to explore the relationship between 5-FU exposure and myelosuppression. In a physiologically-based PK model [24], the activities of the metabolic enzymes regulating the metabolism of Capecitabine to 5-FU are considered to pursue a better prediction of the therapy efficacy. The study [25] aiming for characterizing the PK/PD relationship for LY2835219 developed an integrated model incorporating CDK4/6 inhibition, cell-cycle arrest, and tumor growth inhibition. Altinok et al. proposed a cellular automaton model coupled with a PK model to investigate the effects of circadian rhythms on the therapeutic outcome of 5-FU [26]. Recognizing the benefits brought about by this type of computational model, we developed a mechanistic PK/PD model which encompasses multi-level biological phenomenon to capture the drug actions of 5-FU in the tumor cells.

5-FU exhibits its cytotoxicity through a metabolic pathway that has been widely studied since its approval. 80%-90% of 5-FU is further decomposed into simple molecules through catabolism taking place in the liver by dihydro-pyrimidine dehydrogenase (DPD), while its active forms of metabolites are produced through anabolism in tumor cells [27]. It is 5-FU anabolites that intervene in the normal cellular functions and exhibit deleterious effects on tumor growth. Fluoro-deoxyuridinetriphosphate (FdUTP) and fluorouridine triphosphate(FUTP) are the products of sequential phosphorylations initially catalyzed by thymidine phosphorylase (dThdPase) and uridine phosphorylase (UrdPase) or orotate phosphoribosyl transferase (OPRT), respectively [28, 29]. They are ultimately incorporated in RNA and DNA through the action of DNA polymerase, causing genomic instability. The conversion of 5-FU to fluorodeoxyuridine (FUDR) is followed by the production of fluorodeoxyuridylate (FdUMP) catalyzed by thymidine kinase (TK). FdUMP binds to thymidylate synthase (TS) at its nucleotide site in competition with deoxyuridine monophosphate (dUMP) and forms a ternary complex with a folate analog 5,10-methylenetetrahydrofolate (CH2THF) as a co-substrate, leading to reversible inactivation of TS enzymatic activity and inhibition of thymidine production [30, 31].

Accumulating evidence indicates that TS inhibition is the determinant of 5-FU’s anti-tumor effects and its activity has been demonstrated to be the predictor of tumor response to 5-FU treatment [31–33]. Hence, in our model, the dNTP pool imbalance caused by TS inhibition is attributed to the key factor in the formation of deleterious DNA fragmentation. The intactness of the components in the dNTP pool is required for DNA replication and DNA repair machinery to perform with high fidelity [34]. The thymidine deprivation and resulting dUMP accumulation can give rise to the perturbation of the biosynthesis of the other deoxynucleotides (dATP, dGTP, dCTP) via multiple feedback mechanisms. Interruption of biosynthesis of DNA precursors caused by the deficiency of the enzymes involved in nucleotide metabolism can give rise to futile DNA damage repair and accumulated DSBs [35]. (F)dUTP insertions into DNA facilitated by dUMP accumulation are recognized and excised by uracil DNA glycosylase (UNG), the first responder of the BER pathway [36]. But the uracil bases are incorporated repeatedly, causing the futile cycling repair by BER [36]. The accumulation of BER intermediates such as AP sites and single-strand breaks (SSBs) and persistent breakages triggers the formation of DSBs, resulting in cell cycle arrest, activation of homologous recombination repair(HRR), and apoptosis pathways. Indeed, some studies have established the relationship between the dNTP pool imbalance and 5-FU cytotoxicity [32, 37].

In this study, we constructed a detailed mechanistic model linking the concrete drug actions of 5-FU to the prediction of pharmacodynamic response of colon cancer cells. The cellular model focuses on 5-FU anabolism, RNA and DNA misincorporation, TS inhibition leading to contamination of the dNTP pool, and induction of DSBs when the cell population proceeds through the S phase, all of which are believed to be essential biological events upon the administration of 5-FU. In addition, we used the *γ*-H2AX foci count to approximate DSBs, *γ*-H2AX is the canonical biomarker of DSBs, and its qualitative consistency with DSBs has been well established in the literature [38]. The tumor growth inhibition (TGI) model is built based on the combination of the life span model and the indirect response model, and the dynamics of the normal tumor growth is captured by the logistic growth model involving carrying capacity. Considering the suppressive effect of drug on tumor growth, 5-FU anabolites are modeled to inhibit the tumor growth and 5-FU-induced DSB formation is modeled accelerate the conversion of proliferating cells to damaged cells, which quantitatively captures the observations that the induction of DSBs by 5-FU and 5-FU anabolites are candidate predictive markers of 5-FU treatment efficacy [39, 40]. In addition, the TGI model is constructed and validated using literature tumor volume data observed from the tumor xenografts administered with different dosage regimens. Another advantage of our mechanistic model is that the simulation-based resistance analysis can be conducted to provide the computational assessment of the potential causes of tumor resistance to 5-FU treatment. We not only examined the role of individual reactions in causing tumor resistance to 5-FU but also took into account the interactions among multiple factors. By comparing the time courses corresponding to these simulation scenarios and the time course of the tumor volume measured in 5-FU resistant cell line, we computationally identify which events contribute significantly to the drug resistance. Furthermore, we carried out global sensitivity analysis by applying the Morris method to screen out the subsets of the parameters with considerable effects on model outputs and then applying Sobol analysis that allows for the analysis of parameter interaction effects.

## Materials and methods

**Fig 1:**
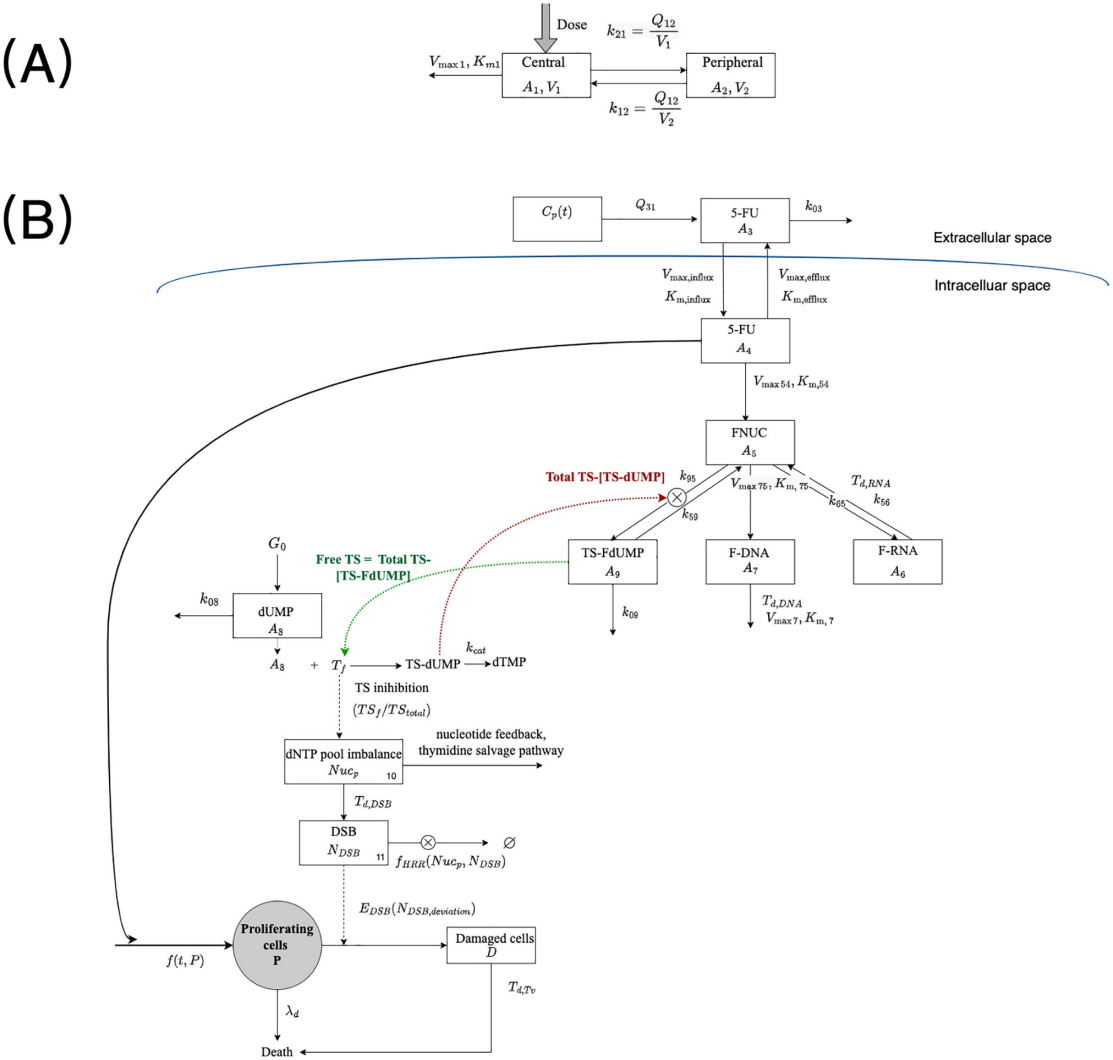
Schematic representation of PK, cellular and tumor growth inhibition (TGI) model. (A) PK model; (B) Cellular and TGI model. *A*_1_:amount of 5-FU in central plasma; *A*_2_: amount of 5-FU in peripheral compartment; *C*_*p*_(*t*): 5-FU concentration in central plasma. *A*_3_: amount of 5-FU in interstitial fluid; *A*_4_: amount of 5-FU in the intra-cellular domain; *A*_5_: amount of 5-FU anabolites; *A*_6_: incorporation of FUTP into RNA; *A*_7_: incorporation of FdUTP into DNA; *A*_8_: amount of dUMP;*A*_9_: amount of TS-FdUMP complex; *TS*_*f*_: amount of free TS; *TS*_*total*_: amount of total TS; *Nuc*_*p*_: extent of dNTP pool imbalance; *N*_*DSB*_: count of 5-FU induced DSBs; *N*_*DSB*,*deviation*_: deviation of 5-FU induced DSBs from baseline; *P*: amount of proliferating cells; *D*: amount of damaged cells. *V*: volumes of compartments; $V_{\text{max}},K_{,m}$: Michaelis–Menten parameters; *Q*: clearance between neighboring compartments; *k*: rate constant; *T*: delaying parameters; *G*_0_: production rate of dUMP;; λ_*d*_: death rate of tumor cells without 5-FU; *f*_*HRR*_: rate of repair of DSBs by HRR. *E*_*DSB*_: measure of contribution of DSBs in producing damaged cells.

Our mechanism-based model is composed of PK, cellular, and tumor growth inhibition (TGI) sub-models, the last two of which constitute the PD model. The model diagram is shown in Fig 1. The PK model is used to compute the concentration-time profile, and the TGI model focuses on the kinetics of tumor growth post-treatment. Our cellular sub-model captures physiological processes at the molecular level and connects the drug distribution in plasma and pharmacological response towards the drug action.

### PK model

The PK model is described by a two-compartment plasma-peripheral model with Michaelis-Menten clearance [8, 41–43]. The central compartment models the drug distribution in the central plasma. All the remaining tissue space is captured by a hypothetical compartment, referred to as the peripheral compartment so that the exchange of drug substances between central blood (compartment 1) and auxiliary region of human body (compartment 2) given by a linear kinetics, and non-linear elimination collectively describe the drug fate in the circulatory system.

The levels of 5-FU in the central plasma (*A*_1_(*t*), pmol⋅mg^−1^) and in the peripheral regions(*A*_2_(*t*), pmol⋅mg^−1^) change according to the Eqs (1) and (2).

$$\begin{array}{c} \frac{dA_{1}\left(t\right)}{dt}=\frac{Q_{12}}{V_{2}}A_{2}\left(t\right)-\frac{Q_{12}}{V_{1}}A_{1}\left(t\right)-\frac{V_{\text{max}\;1}\cdot C_{1}\left(t\right)}{K_{m1}+C_{1}\left(t\right)} \end{array}$$ (1)

$$\begin{array}{c} \frac{dA_{2}\left(t\right)}{dt}=\frac{Q_{12}}{V_{1}}A_{1}\left(t\right)-\frac{Q_{12}}{V_{2}}A_{2}\left(t\right) \end{array}$$ (2)

$$\begin{array}{c} C\left(t\right)=\frac{A(t)}{V} \end{array}$$ (3)

where *Q*_12_ is volume of 5-FU cleared inter-compartmentally between central and peripheral space per minute. *V*_1_ and *V*_2_ are the volumes of the central and peripheral compartments, respectively. *V*_max 1_ and *K*_m1_ describe the process of elimination of 5-FU from central plasma.

### Cellular model

The cellular model is built using a compartmental modeling approach, which is one of the common techniques utilized to model biochemical pathways. In the previous model involving similar molecular complexity developed by Wolf et al [44], the drug transfer from interstitial fluid to intra-cellular space and the emergence of products of drug action are considered. In our work, we construct a model including a more detailed description of physiological events associated with 5FU cytotoxicity. The cellular model considers the 5-FU concentration in interstitial fluid (compartment 3), formation of 5-FU anabolites (compartment 5), insertion of FUTP, FdUTP into RNA and DNA (compartment 6 and 7) binding of FdUMP to TS (compartment 9), changes in the components of the dNTP pool caused by reduced TS catalytic activity (compartment 10) and DSB induction as a response to imbalanced dNTP pool (compartment 11). For the purpose of modeling, the compounds are assumed to dissolve in a spatially homogenous environment and be confined in the compartments, in which certain reactions with the respective compounds as reactants would take place. The rate of change of these reactions are represented by the influx and efflux of corresponding compartments. The dynamics of those species are subject to a group of differential equations described by the law of mass action and the Michaelis-Menten kinetics. The time evolution of physiological intermediates, as the output of the cellular model, is involved in the determination of drug effect measures, driving the ultimate pharmacodynamic responses.

#### 5-FU in interstitial fluid and its anabolism

The differential equations describing the evolution of 5-FU in interstitial fluid (*A*_3_(*t*), pmol⋅mg^−1^), intra-cellular 5-FU (*A*_4_(*t*), pmol⋅mg^−1^) and 5-FU anabolites(*A*_5_(*t*), pmol⋅mg^−1^) are formulated in Eqs (4), (5) and (6). The kinetics of 5-FU in interstitial fluid (*A*_3_(*t*), pmol⋅mg^−1^) is governed by unidirectional transmission from central plasma [45, 46], first-order elimination from the extracellular domain (*k*_03_) and update and removal of 5-FU molecule across the tumor cell membrane [46]. The influx and efflux transporters anchored on the cell membrane contribute to uptake and clearance of 5-FU drug molecules [47]. Considering that the drug diffusion into the target cell is limited by the maximum number of available uptake transporters, the Michaelis kinetics, characterized by the saturation curve, is applied to describe the process of absorption of 5-FU by tumor cells. It is difficult to detect the individual low molecular-weight 5-FU anabolites (i.e. fluoronucleotides and fluoronucleosides, denoted as FNUC) accurately despite using high precision techniques [48]. Therefore, FNUCs are collectively represented by variable *A*_5_(*t*) and are modeled to be uniformly distributed and non-diffusing in one compartment. The rate of formation of *A*_5_(*t*) is through 5-FU anabolism. The decomposition of the TS-FdUMP complex and removal of 5-FU from RNA account for the replenishment of *A*_5_(*t*). The decrease of amount of *A*_5_(*t*) is regulated by the incorporation of FdUTP and FUTP into DNA and RNA and attachment of FdUMP on TS.

The time-dependent levels of incorporated FU residues into RNA(*A*_6_(*t*), FU-RNA, and DNA((*A*_7_(*t*), FU-DNA) are the solution of Eqs (7) and (8). The influxes into compartment 6 and compartment 7 quantitatively capture the incorporation of 5-FU into RNA and DNA, receptively, the rates of which are driven by the kinetics of 5-FU anabolites (*A*_5_(*t*)). The efflux terms in Eqs (7) and (8) describe the efficiency of excision of uracil bases by cell-intrinsic repair machineries. Different from the linear kinetics for FU-RNA, Michaelis-Menten kinetics is applied to FU-DNA since FdUTP insertion into DNA and restoring FU-containing DNA is regulated by protein enzymes. According to the experimental data regarding 5-FU insertion into genomes, the process of removing genomic 5-FU is not instantaneous but rather lasts for days. It is also shown in the literature that the initiation of cell cycle checkpoint and futile cycle DNA repair are the two main causes of delayed DNA repair process. The persistence of uracil analogs in genomes challenges genomic stability, initiating the cell cycle checkpoint and preventing cell cycle progression. The cellular repair machineries are precipitously assembled. However, the futile cycling repair, the causes of which have been discussed at length in the introduction, leads to the accumulation of unrepaired mismatched bases and impeded operation of DNA repair proteins. In conformity with the underlying mechanism, the rates of loss of FU-RNA and FU-DNA are the function of past states of FU-RNA and FU-DNA, realized by two delaying parameters (*T*_*d*,*RNA*_, *T*_*d*,*DNA*_). Specifically, for *t* ≤ *T*_*d*,*RNA*_(*T*_*d*,*DNA*_), the loss of genomic 5-FU in RNA(DNA) is modeled as the kinetics without delay, while, for *t* > *T*_*d*,*RNA*_(*T*_*d*,*DNA*_), the elimination of FU-RNA (FU-DNA) is considered as a process that depends on the past state of FUTP (FdUTP) insertion into RNA(DNA).

Overall, the differential equations governing the dynamics of extracellular 5-FU (*A*_3_(*t*), pmol⋅mg^−1^), intra-cellular 5-FU (*A*_4_(*t*), pmol⋅mg^−1^), 5-FU anabolites(*A*_5_(*t*), pmol⋅mg^−1^), FU- RNA(*A*_6_(*t*), pmol⋅mg^−1^) and FU-DNA (*A*_7_(*t*), pmol⋅mg^−1^) are shown below.

$$\begin{array}{c} \frac{dA_{3}\left(t\right)}{dt}=Q_{31}C_{p}\left(t\right)+\frac{V_{\text{max},\text{efflux}}A_{4}\left(t\right)}{K_{\text{m,efflux}}+A_{4}\left(t\right)}-\frac{V_{\text{max},\text{influx}}A_{3}\left(t\right)}{K_{\text{m,influx}}+A_{3}\left(t\right)}-k_{03}A_{3}\left(t\right) \end{array}$$ (4)

$$\begin{array}{c} \frac{dA_{4}\left(t\right)}{dt}=-\frac{V_{\text{max},\text{efflux}}A_{4}\left(t\right)}{K_{\text{m,efflux}}+A_{4}\left(t\right)}+\frac{V_{\text{max},\text{influx}}A_{3}\left(t\right)}{K_{\text{m,influx}}+A_{3}\left(t\right)}-\frac{V_{\text{max},54}A_{4}\left(t\right)}{K_{\text{m,54}}+A_{4}\left(t\right)} \end{array}$$ (5)

$$\begin{array}{c} \begin{array}{cc} \frac{dA_{5}\left(t\right)}{dt} & =\frac{V_{\text{max}\;54}\cdot A_{4}\left(t\right)}{K_{m54}+A_{4}\left(t\right)}+\frac{k_{56}A_{6}\left(t\right)}{{\left(1+k_{06}A_{6}\left(t-T_{d,RNA}\right)\right)}^{\gamma_{\text{lag,RNA}}}}+k_{59}A_{9}\left(t\right)\\ & -(k_{65}A_{5}\left(t\right)+\frac{V_{\text{max}\;75}\cdot A_{5}\left(t\right)}{K_{m75}+A_{5}\left(t\right)}+k_{95}\left(TS_{total}-TS-dUMP\right)A_{5}\left(t\right)) \end{array} \end{array}$$ (6)

$$\begin{array}{c} \frac{dA_{6}\left(t\right)}{dt}=k_{65}A_{5}\left(t\right)-\frac{k_{56}A_{6}\left(t\right)}{{\left(1+k_{06}A_{6}\left(t-T_{d,RNA}\right)\right)}^{\gamma_{\text{lag,RNA}}}} \end{array}$$ (7)

With *A*_6_(*t*) = 0, for −*T*_*d*,*RNA*_ ≤ *t* ≤ 0

$$\begin{array}{c} \frac{dA_{7}\left(t\right)}{dt}=\frac{V_{\text{max},75}\cdot A_{5}\left(t\right)}{K_{m,75}+A_{5}\left(t\right)}-\frac{V_{\text{max},7}\cdot A_{7}\left(t\right)}{(K_{m,7}+A_{7}\left(t\right){\left(1+k_{07}A_{7}\left(t-T_{d,DNA}\right)\right)}^{\gamma_{\text{lag,DNA}}}} \end{array}$$ (8)

With *A*_7_(*t*) = 0, for −*T*_*d*,*DNA*_ ≤ *t* ≤ 0.

In which *C*_*p*_(*t*) denotes concentration of 5-FU in central plasma, which is solely defined by the PK model, *Q*_31_ is the volume of 5-FU cleared from central plasma to extracellular space per unit time, *V*_max,influx_ along with *K*_m,influx_ describes the saturable uptake, *V*_max,efflux_ and *K*_m,efflux_ describe the saturable removal, *k*_03_ is the first order rate of eliminating 5-FU from interstitial fluid, *V*_max,54_ is the maximum rate of 5-FU anabolism, and *K*_m54_ is the corresponding half saturation value. *k*_56_ and *k*_65_ are the first order rates of incorporating FUTP into and removing FUTP from RNA. *k*_06_ and *T*_*d*,*RNA*_ as well as *k*_07_ and *T*_*d*,*DNA*_, describe the delayed excision of incorporated FU residues from RNA and DNA. *γ*_lag,RNA_ and *γ*_lag,DNA_ are used to adjust curve shape. The DNA polymerase with limited catalytic capability controls the insertion of FdUTP into DNA, expressed by parameters *V*_max,75_ and *K*_m,75_. *V*_max,7_ and *K*_m,7_ represent the rate of removal of FdUTP from DNA by UNG. Initial conditions for each compartment are zero. The units of incorporation of 5-FU into DNA and RNA are fmol⋅ *μ*g^−1^ DNA and pmol⋅ *μ*g^−1^ RNA, as shown in the data source literature, which are converted to pmol⋅ mg^−1^ tissue through the concentration of DNA and RNA(0.2 *μ*g/mg tissue and 2 *μ*g/mg tissue respectively) [49].

The next dynamics we need to model is the binding of FdUMP to TS, which is considered to be the main contributor to 5-FU cytotoxicity. The study on analyzing the protein structure of E.coli TS [50] found that the methylation of dUMP to dTMP by TS with CH2THF as the methyl donor is a sequential process in which the dUMP binds at the active site of TS before CH2THF does. However, from the quantitative perspective, the differences in rate between the nucleotide/folate-ordered and random binding mechanism of reaction at TS is minimal [51]. Thus, for the sake of simplicity, we assume that the reaction involving dUMP is subject to rapid-equilibrium random mechanism instead of the ordered bi-substrate reaction. Since FdUMP and dUMP share the common biochemical properties in terms of covalent binding to TS [52], the pattern of interaction between FdUMP and TS is also in the manner of rapid equilibrium, demonstrated by the first term in Eq (9). It is also assumed that the free TS is saturated with CH2FH4.

To model the rate of TS-FdUMP complex formation, the quantity of TS enzyme over time is also considered, as shown in Eq (10). It is a consistent finding that the TS expression increases in response to 5-FU treatment [53–55]. TS expression at both protein and mRNA levels are regulated by multiple factors such as activation of cyclin/CDK complexes and its auto-regulatory feedback [56]. The increase in TS level after 5-FU exposure, with contributing factors still indefinite, may be related to gene amplification and disrupted synthesis regulation [56]. Therefore, it is more appropriate to estimate the total empirically content of TS by a mono-exponential equation.

Free FdUMP binding sites (*TS*_*f*_) is expressed in terms of the all available FdUMP binding sites (*TS*_*total*_), and FdUMP-bound TS enzyme (TS-FdUMP). In the literature that we use as the data source, the experimental measurements of *TS*_*total*_ are carried out after and *TS*_*f*_ are carried out before the dissociation of the ternary complex respectively [57]. Pretreatment *TS*_*f*_ level is set as initial level of *TS*_*total*_. The amounts of *TS*_*total*_ and *TS*_*f*_ upon exposure to 5-FU are determined by Eqs (10) and (11).

The kinetics of TS-FdUMP complex(*A*_9_(*t*), pmol⋅mg^−1^) is expressed by Eq (9). Eqs (10) and (11) are the analytical expressions for *TS*_*f*_ and *TS*_*total*_.

$$\begin{array}{c} \frac{dA_{9}\left(t\right)}{dt}=k_{95}\left(TS_{total}-\left[TS-dUMP\right]\right)A_{5}\left(t\right)-k_{59}A_{9}\left(t\right)-k_{09}A_{9}\left(t\right) \end{array}$$ (9)

$$\begin{array}{c} TS_{total}=TS_{0}[\alpha +\left(1-\alpha \right)\text{exp}(-k_{d}t)] \end{array}$$ (10)

$$\begin{array}{c} TS_{f}=TS_{total}-\left[TS-FdUMP\right] \end{array}$$ (11)

In which the rate of enzyme-substrate complex formation is denoted as *k*_95_. *k*_59_ represents the complex dissociation rate. The complex dissociation is accompanied by the release of FdUMP and the recovery of free TS. *k*_09_ represents the first-order process of complex degradation. The production rate of the TS-FdUMP complex is proportional to (*TS*_*total*_ − [*TS-dUMP*]), implicating that the competition between dUMP and FdUMP on the TS nucleotide binding site impedes the attachment of FdUMP to TS. *α* is the ratio of increased TS synthesis rate in the presence of drug to the normal synthesis rate of TS, *k*_*d*_ is the ratio of degradation rate measured in treated cells and normal degradation rate constant, *TS*_0_ is the level of TS protein prior to 5-FU administration.

It is assumed that there is an instantaneous equilibrium relationship between free *dUMP* and TS-dUMP-folate ternary complex. Therefore, the complex [*TS-dUMP*] is given by Eq (12).

$$\begin{array}{c} \left[TS-dUMP\right]=\frac{dUMP_{f}\cdot TS_{f}}{K_{dUMP}} \end{array}$$ (12)

where *K*_*dUMP*_ is the dissociation constant for the enzyme-substrate complex and is the ratio of reverse and forward rate constants.

It is more convenient to express complex [*TS-dUMP*] as a function of free TS and the total dUMP(*dUMP*_total_) that is the sum of free dUMP and [*TS-dUMP*] complex, since the quantities of free TS and total dUMP are variables considered in the cellular model. By solving free dUMP out of total dUMP and [*TS-dUMP*], we can get the new expression for variable *dUMP*_*f*_ that is $\frac{dUMP_{total}}{\left(1+\frac{\left[TS_{f}\right]}{K_{dUMP}}\right)}$. Following this, [*TS-dUMP*] can be further expressed in terms of *dUMP*_total_ and *TS*_*f*_, as shown in Eq (13).

$$\begin{array}{c} \begin{array}{cc} \left[TS-dUMP\right] & =\frac{dUMP_{total}}{\left(1+\frac{TS_{f}}{K_{dUMP}}\right)}\cdot \frac{TS_{f}}{K_{dUMP}}\\ & =\frac{dUMP_{total}\cdot TS_{f}}{K_{dUMP}+TS_{f}} \end{array} \end{array}$$ (13)

The zero order synthesis, production of dTMP catalyzed by TS and degradation influence time-amount curve of total dUMP(*A*_8_(*t*), pmol⋅mg^−1^) (Eq (14)).

$$\begin{array}{c} \frac{dA_{8}\left(t\right)}{dt}=G_{0}-r_{TS}-k_{08}A_{8}\left(t\right) \end{array}$$ (14)

where *G*_0_ is the zero-order rate constant of dUMP synthesis. *k*_08_ is the first-order degradation rate of dUMP. *r*_*TS*_ represents the rate of conversion of dUMP to dTMP catalyzed by TS, which is responsible for the loss of total dUMP. The expression of *r*_*TS*_ is shown in Eq (15) and is linearly dependent on the amount of dUMP/TS/folate ternary complex through the catalytic rate constant *k*_*cat*_.

$$\begin{array}{c} \begin{array}{cc} r_{TS} & =k_{cat}(A_{8}\left(t\right)-{\text{dUMP}}_{f})\\ & =k_{cat}\left[TS-dUMP\right]\\ & =k_{cat}\cdot \frac{dUMP_{total}\cdot TS_{f}}{K_{dUMP}+TS_{f}} \end{array} \end{array}$$ (15)

#### dNTP pool imbalance

The perturbation of components in the DNA precursor pool (denoted as *Nuc*_*p*_) is influenced by aggregated effects of 5-FU stimulated TS inhibition, cellular nucleotide salvage pathway, and nucleotide feedback. *Nuc*_*p*_ is represented by the variation of the total amount of deoxynucleotides at a given time point versus the corresponding control level, following the same methodology as [58]. The mathematical expression for *Nuc*_*p*_ is determined as Eq (16).

$$\begin{array}{c} \sqrt{\text{ln}(\frac{\sum_{x\in \{\;\text{all}\;\text{dNTPs}\;\}}x}{\sum_{x_{0}\in \left\{\;\text{all}\;\text{dNTPs}\;\right\}}x_{0}}{)}^{2}} \end{array}$$ (16)

In which *x*_0_ and *x* denote the size of each constituent of dNTP pool before and after 5-FU treatment, respectively. The initial value of *Nuc*_*p*_ is zero, implicating that prior to treatment there is no tendency towards unusual nucleotide biosynthesis. As the level of all dNTPs decrease or increase drastically from the baseline *x*_0_, the perturbation measure would increase accordingly. The data used for model fitting is also converted to the measure of perturbation according to Eq (16).

The time profiles of *TS*_*f*_ and *TS*_*total*_, determined in the cellular model, are connected with changes of *Nuc*_*p*_ by introducing a new variable called perturbation in TS inhibition (*TS*_*p*_) into this submodel. The degree of TS inhibition is evaluated by the ratio of the number of free FdUMP binding sites (*TS*_*f*_, pmol⋅mg^−1^) and the total available TS binding sites (*TS*_*total*_, pmol⋅mg^−1^) [56]. *TS*_*p*_ is quantified as the deviation of TS inhibition measured post-treatment (*TS*_*f*_/*TS*_*total*_) from the normal portion of free TS levels (*TS*_*f*0_/*TS*_*total*0_) (Eq (17)).

$$\begin{array}{c} TS_{p}=\frac{TS_{f}/TS_{total}}{TS_{f0}/TS_{total0}} \end{array}$$ (17)

With initial condition of 1, the value of *TS*_*p*_ varies within the range 0 to 1. *TS*_*p*_ vanishes when TS nucleotide binding sites is fully occupied by FdUMP.

We applied and modified the scheme previously developed by Setzer et al [58] to simulate the dNTP pool imbalance induced by 5-FU. The degree of dNTP pool imbalance is quantified based on the two assumptions: 1). The 5-FU cytotoxicity is directly related to dTTP and the alteration of dNTP is the direct and immediate product of TS inhibition; 2). Any disruption of dNTP supplies as a result of TS inhibition would impede the normal process of DNA replication. The kinetics of dNTP pool imbalance is decided by the two differential equations, one for variable *Nuc*_*p*_ as discussed above and one for variable *U*. The cellular nucleotide salvage pathway is activated in the face of intra-cellular thymidine starvation caused by TS inhibition. Thus *U* is invented to describe the propensity of the cellular nucleotide salvage pathway to diminish the disturbance in the levels of constituents in the pool. The dynamics of *U* follow the pattern of a negative feedback loop (Eq (19)). As the first terms in Eqs (18) and (19) show, the rates of production of *Nuc*_*p*_ and *U* both increase as the perturbation in TS inhibition are elevated, and finally converge to corresponding upper bounds(*k*_1_ and *k*_10_). Therefore, as no FdUMP is attached to TS, all the binding sites on TS are available for dUMP, and the fluctuation in dNTP would return to baseline level. In addition, the collective effects of *TS*_*p*_, *Nuc*_*p*_ and *U* impose negative components to the rate of change of *Nuc*_*p*_ and are represented by the third term in Eq (18) which is smaller for the smaller level of *TS*_*p*_ and larger for the greater level of *Nuc*_*p*_ and *U*. Besides, the second term in Eq (18), solely dependent on the level of *Nuc*_*p*_, describes the amelioration of disturbance by biochemical interactions among components in the dNTP pool. The kinetics of *Nuc*_*p*_ and *U* are described by the differential equations(Eqs (18) and (19)).

$$\begin{array}{c} \begin{array}{cc} \frac{dNuc_{p}\left(t\right)}{dt} & =\frac{k_{1}{\left(1-TS_{p}\right)}^{\gamma_{dNTP}}}{k_{2}^{\gamma_{dNTP}}+{\left(1-TS_{p}\right)}^{\gamma_{dNTP}}}-\frac{k_{3}Nuc_{p}}{1+k_{4}Nuc_{p}}\\ & -\frac{k_{5}U\cdot Nuc_{p}}{\left(1+k_{6}U)(1+k_{7}^{\gamma_{dNTP}}{\left(1-TS_{p}\right)}^{\gamma_{dNTP}})(1+k_{B}Nuc_{p}\right)} \end{array} \end{array}$$ (18)

$$\begin{array}{c} \frac{dU}{dt}=\frac{k_{10}{\left(1-TS_{p}\right)}^{\gamma_{\text{dNTP}}}}{k_{8}^{\gamma_{\text{dNTP}}}+{\left(1-TS_{p}\right)}^{\gamma_{\text{dNTP}}}}-\frac{k_{9}U}{1+k_{A}U} \end{array}$$ (19)

In which *γ*_*dNTP*_ is a hill parameter. All the other parameters are the unitless quantities.

#### DSB induction

dNTP pool imbalance and cellular repair pathways are implicated in the production and elimination of DSBs, denoted as *N*_*DSB*_(*t*) in the model. The intactness of DNA precursors in the dNTP pool partially guarantees the success of DNA biosynthesis and DNA damage repair [32]. The perturbed dNTP pool caused by TS inhibition would result in double strand DNA break during DNA replication in the S phase [32, 37]. In response to the aberrant DNA duplex structure, the cell cycle checkpoints are triggered to allow the repair machinery to restore the genomic stability; if the DNA repair fails, apoptosis pathway would be activated to clear the damaged cells [34].

The literature data shows that the induction of DSBs caused by 5-FU is delayed following the occurrence of dNTP pool imbalance. Such an assumption is also biologically reasonable since the two cellular events are occurred at different molecular levels and are related to cell cycle progression. In the absence of detrimental effects of dNTP pool imbalance, the generation of severe DNA lesions depends on the zero-order rate constant *k*_0_. The outflow term $\frac{V_{\text{max},HR}\cdot N_{DSB}\left(t\right)}{K_{m,HR}+N_{DSB}\left(t\right)}$ stands for the efficiency of normal DNA repair responses. Mathematically, the dynamics of DSB generation (*N*_*DSB*_(*t*)) for 0 ≤ *t* ≤ *T*_*d*_ is governed by Eq (20)

$$\begin{array}{c} \frac{dN_{DSB}\left(t\right)}{dt}=k_{0}-\frac{V_{\text{max},HR}N_{DSB}\left(t\right)}{K_{m,HR}+N_{DSB}\left(t\right)} \end{array}$$ (20)

By contrast, the rate of formation of 5-FU-induced DSB depends on the past states of perturbation in dNTP pool (*Nuc*_*p*_) and is modeled as a full sigmoid formula so that the amount of DNA fragmentation caused by 5-FU may converge to an upper limit *V*_max,*dNTP*_. The elimination of DSB is mediated not only by DNA repair response, but also by the availability of DNA precursors [59]. It has been suggested that the polymerization of DNA in the course of homologous recombination repair (HR) is obstructed by imbalanced nucleotide pools resulting from FdUMP binding to TS [60]. Therefore, the kinetics of HR efficiency decreases as the perturbation in the dNTP pool is elevated. For *t* > *T*_*d*_, the dynamics of DSB induction is governed by Eq 21.

$$\begin{array}{c} \begin{array}{cc} \frac{dN_{DSB}\left(t\right)}{dt} & =k_{0}(1+\frac{V_{dNTP}\cdot Nuc_{p}{\left(t-T_{d,DSB}\right)}^{\gamma_{DSB}}}{K_{dNTP}^{\gamma_{DSB}}+Nuc_{p}{\left(t-T_{d,DSB}\right)}^{\gamma_{DSB}}})\\ & -\frac{V_{\text{max},HR}\cdot N_{DSB}\left(t\right)}{\left(K_{m,HR}+N_{DSB}\left(t\right)\right)\left(Nuc_{p}{\left(t-T_{d,DSB}\right)}^{\gamma_{DSB}}\cdot k_{i}^{\gamma_{DSB}}+1\right)} \end{array} \end{array}$$ (21)

with *Nuc_p_*(*t*) = 0 for −*T*_*d*,*DSB*_ ≤ *t* ≤ 0.

In which the delaying effect of dNTP pool imbalance on *N*_*DSB*_(*t*) is modeled as the explicit delay parameter *T*_*d*,*DSB*_. *γ*_*DSB*_ is used to adjust the shape of the curve.

5-FU-mediated disturbance in genomic stability (*N*_*DSB*,*deviation*_(*t*)) is qualified on the basis of kinetics of *N*_*DSB*_(*t*) and is incorporated into the TGI model. *N*_*DSB*,*deviation*_(*t*) measure deviation of the DSBs exclusively caused by dNTP pool contamination from the time-dependent baseline level, given by Eq (22)

$$\begin{array}{c} N_{DSB,deviation}\left(t\right)=\frac{N_{DSB}\left(t\right)-N_{DSB_{0}}\left(t\right)}{N_{DSB_{0}}\left(t\right)} \end{array}$$ (22)

In which *N*_*DSB*,*deviation*_(*t*) is a unitless quantity. The time course of *N*_*DSB*,0_(*t*) is simulated based on the placebo treatment [61]. The value of *N*_*DSB*,*deviation*_(*t*) is zero when no drug is administered and $N_{DSB_{0}}\left(t\right)$ is the number of DSB in the absence of any medical intervention and is varied with time as it is probable for other endogenous factors to induce DSBs to a lesser extent.

### Tumor growth inhibition model

The TGI model is built on the basis of the indirect response model and life span model. It is assumed that the overall tumor cell population, denoted as *T*, comprises proliferative and damaged cells. The pharmaceutical response examined in this module is the change of tumor volume(*cm*^3^). Accordingly, the tumor growth inhibition (TGI) model is composed of the differential equations governing the kinetics of proliferating cell population (*P*(*t*)) and damaged quiescent cell population (*D*(*t*)), as shown in Eqs (23) and (24).

$$\begin{array}{c} \begin{array}{cc} \frac{dP(t)}{dt} & =\frac{IC_{50}^{\gamma_{\text{Tv}}}}{IC_{50}^{\gamma_{\text{Tv}}}+A_{5}{\left(t\right)}^{\gamma_{\text{Tv}}}}\lambda_{g}P\left(t\right)\cdot (1-\frac{P(t)}{P_{\text{max}}})\\ & -(1+\frac{E_{\text{max},damage}N_{DSB,deviation}{\left(t\right)}^{\gamma_{\text{Tv}}}}{EC_{50,damage}^{\gamma_{\text{Tv}}}+N_{DSB,deviation}{\left(t\right)}^{\gamma_{\text{Tv}}}})\lambda_{d}P\left(t\right) \end{array} \end{array}$$ (23)

$$\begin{array}{c} \begin{array}{cc} \frac{dD(t)}{dt} & =\lambda_{d}\frac{E_{\text{max},damage}N_{DSB,deviation}{\left(t\right)}^{\gamma_{\text{Tv}}}}{EC_{50,damage}^{\gamma_{\text{Tv}}}+N_{DSB,deviation}{\left(t\right)}^{\gamma_{\text{Tv}}}}P\left(t\right)\\ & -\lambda_{d}\frac{E_{\text{max},damage}N_{DSB,deviation}^{\gamma_{\text{Tv}}}\left(t-T_{\text{d,Tv}}\right)}{EC_{50,damage}^{\gamma_{\text{Tv}}}+N_{DSB,deviation}^{\gamma_{\text{Tv}}}\left(t-T_{\text{d,Tv}}\right)}P\left(t-T_{\text{d,Tv}}\right) \end{array} \end{array}$$ (24)

$$\begin{array}{c} T=P+D \end{array}$$ (25)

$$\begin{array}{c} P\left(0\right)=T_{0},P\left(t\right)=0,t<0,D\left(t\right)=0,t<0 \end{array}$$ (26)

Where *T*_d,Tv_ is the mean life span of damaged cells. Cells *D* would be processing the damage for *T*_d,Tv_ until they are eliminated from the entire tumor cell population. *A*_5_(*t*) is amount 5-FU anabolites at time *t*, computed in cellular model. *IC*_50_ is the concentration of 5-FU anabolites that causes the 50% decrease in tumor proliferation rate. *E*_max,*damage*_ is the maximal fractional factor of DSB-induced stimulatory effect on the production of damaged quiescent cells. *EC*_50,*damage*_ is the level of *N*_*DSB*,*deviation*_ necessary to produce the half-maximal stimulatory effect. λ_*g*_ is the tumor growth rate. λ_*d*_ is the natural death rate. *P*_max_ is the carrying capacity of tumor growth. *γ*_Tv_ is a curve-adjusting parameter, whose value is fixed as 0.2. The initial value of *P*(*t*) is the last measurement of tumor volumes before administration starts, while the initial value of *D*(t) is zero.

The TGI model incorporates two mechanistic components, 5-FU anabolites and 5-FU induced DSBs to account for the inhibition of tumor proliferation by 5-FU treatment. Their individual effects on the pharmacological responses are mathematically formulated by full sigmoid *E*_max,*damage*_ model, giving rise to *E*_*a*_(*t*) (Eq (27)) and *E*_*DSB*_(*t*) (Eq (28))

$$\begin{array}{c} E_{a}\left(t\right)=\frac{IC_{50}^{\gamma_{\text{Tv}}}}{IC_{50}^{\gamma_{\text{Tv}}}+A_{5}{\left(t\right)}^{\gamma_{\text{Tv}}}} \end{array}$$ (27)

whose value is subject to the upper limit of 1. The smaller the value of *IC*_50_, the more responsive tumor cells would be to the suppressive effects imposed by 5-FU anabolites.

Likewise, *N*_*DSB*,*deviation*_(*t*) is presumed to enhance the loss of proliferative cells with the rate given by *E*_*DSB*_(*t*)

$$\begin{array}{c} E_{DSB}\left(t\right)=\frac{E_{\text{max},damage}N_{DSB,deviation}^{\gamma_{\text{Tv}}}\left(t\right)}{EC_{50,damage}^{\gamma_{\text{Tv}}}+N_{DSB,deviation}^{\gamma_{\text{Tv}}}\left(t\right)} \end{array}$$ (28)

The maximum allowable value of *E*_*DSB*_(*t*) is *E*_max,*damage*_. *E*_max,*damage*_, *EC*_50,*damage*_ and *IC*_50_ are drug-sensitivity parameters and modulate the potency of the chemotherapy.

Two different scenarios associated with the TGI model are discussed in detail.

1. Tumor dynamics in the absence of drug intervention
  The dynamics of proliferative cells in the absence of drug intervention is mediated by Eq (29).
  $$\begin{array}{c} \frac{dP(t)}{dt}=\lambda_{g}P\left(t\right)\cdot (1-\frac{P(t)}{P_{\text{max}}})-\lambda_{d}P\left(t\right) \end{array}$$ (29)
  In normal circumstances, the proliferative tumor cells would grow according to the logistic model and naturally shrink according to the rate constant λ_d_. Linear constant λ_*d*_ regulates the rate of the conversion from the proliferating cells to dead cells which is not affected by chemotherapy. Since in this case, there is no extra DSBs that are triggered by 5-FU treatment, the term ($\frac{E_{\text{max},damage}N_{DSB,deviation}^{\gamma_{\text{Tv}}}}{EC_{50,damage}^{\gamma_{\text{Tv}}}+N_{DSB,deviation}{\left(t\right)}^{\gamma_{\text{Tv}}}}$) is zero, meaning that no damaged cells emerge from normally cycling cell population. Taken together, the three parameters λ_*g*_, *P*_max_, λ_d_ outline the tumor growth pattern in the absence of medical intervention.
2. Treatment responses
  With 5-FU treatment, the tumor progression is assumed to be subject to 5-FU anabolites and DSBs induced by the perturbed dNTP pool. The effects of two quantities are modeled as hill functions and serve as system biomeasures driving tumor dynamics, as shown in Eq (24). The biochemical reactions mediated by 5-FU anabolites result in abnormality in DNA replication and repair, leading to the incomplete preparation for cell division. Such feature is reflected by the term *E*_*a*_(*t*) that is in inverse proportion to the concentration of 5-FU anabolites. Besides, as a contributory factor in 5-FU cytotoxicity, 5-FU-induced DSBs are responsible for the production of damaged cells with a rate *E*_*DSB*_(*t*). Therefore, in our model design, the increase of quiescent tumor cells is driven by the irreversible transition from proliferative tumor cells instead of their proliferation. The tumor cells that incur DSB lesions are not deemed to be eliminated from the entire tumor cell population instantaneously after the onset of DSB, and yet they would undergo progressive stages of damage before they die, which is biologically plausible since the processing of DSB is related with time-dependent signal cascades regulating cell cycle progression. To that end, the life span model, as reviewed in literature [62, 63], is implemented to capture the elongated survival time of non-growing cells. As components of the life span model scheme, the delay differential equation (DDE) is incorporated in the TGI model and describes the delayed onset of the observed drug effects. *T*_d,Tv_ denotes the mean transit time through all the damage stages for *P*(*t*). Therefore, in the case of 5-FU treatment, the alterations of variable *A*_5_(*t*), *N*_*DSB*,*deviation*_(*t*) directly change the dynamics of tumor growth. The damaged cells would emerge once 5-FU induced DSB is above the baseline value $N_{DSB_{0}}\left(t\right)$. Between the two consecutive doses, *N*_*DSB*,*deviation*_(*t*) and *A*_5_(*t*) are expected to return to zero and the change in tumor volume would then become a function of λ_*g*_, *P*_max_, λ_d_, claiming the pattern of unperturbed tumor growth. Besides, the rate constant of tumor natural death and the baseline rate of irreversible conversion from the proliferative to quiescent cells are assumed to be equal toλ_*d*_.

### Model implementation and Parameter estimation

We utilized the Bayesian inference method to estimate the expected values of the model parameters as the Bayesian inference can capture the uncertainty of model parameters given the experimental data. Specifically, the Markov chain Monte Carlo (MCMC) technique is applied to obtain the approximated expected values. In order to address the computational intensity and the difficulty of convergence of the posterior distributions when the model parameters are estimated simultaneously, we conducted the parameter estimations in a sequential manner as follows: We fitted the model parameters of the plasma concentration first and fixed the parameter values as the means of the samples generated by the MCMC technique to estimate the parameters defined in the cellular model. In a similar way, the expected values of cellular model parameters were fixed for fitting of the TGI model. Accordingly, the model parameters are collected in four disjoint sets, denoted as **Θ**_***i***_, *i* = 1…4. Specifically, **Θ**_**1**_ stands for the set of PK parameters; **Θ**_**2**_ represents the set of parameters regulating the kinetics of variables ranging from *A*_3_ (5-FU in the interstitial fluid) to *A*_9_(TS-FdUMP complex); **Θ**_3_ represents the set of the parameters in the ODE equations for *Nuc*_*p*_, and *N*_*DSB*_. **Θ**_**4**_ represents the parameters in the TGI model. The posterior distribution is formulated by the product of the likelihood function and the prior distribution. The likelihood function is expressed through the additive noise model describing the difference between the observed value of the model variables and their predictions. The likelihood function for estimating the parameters in the parameter set Θ_*i*_, *i* = 1…4 is calculated as Eq (30):

$$\begin{array}{c} \begin{array}{cc} L_{i}\left(d_{i}|\Theta_{i},\sigma_{i}\right) & =\prod_{j=1}^{n_{i}}L_{ij}\left(d_{ij}|\Theta_{i},\sigma_{ij}\right)\\ & =\prod_{j=1}^{n_{i}}\frac{1}{{\left(2\pi \sigma_{ij}^{2}\right)}^{L_{ij}/2}}\text{exp}(-\frac{1}{2\sigma_{ij}^{2}}\left|\right|f_{ij}\left(\Theta_{i}\right)-d_{ij}{\left|\right|}^{2}) \end{array} \end{array}$$ (30)

where $d_{ij},i=1\ldots 4,j=1\ldots n_{i},d_{ij}\in R^{L_{ij}}$ represents the time series data of the *j* th model variable(denoted as *R*_*ij*_) whose kinetics are governed by the parameters in **Θ**_***i***_. The difference between each measurement and model prediction of *R*_*ij*_ is described by the measurement noise *ε*_*ij*_. We assumed that *ε*_*ij*_ are independent random variables and normally distributed with zero mean and the variance $\sigma_{ij}^{2}$. Instead of keeping *ε*_*ij*_ constant, we treated them as random variables whose marginal distributions can be learned from the data. $f_{ij}\left(\Theta_{i}\right)\in R^{L_{ij}}$ represents the simulated time course of *j* th variable using the parameter set **Θ**_*i*_. The choice of the prior distribution for each model parameter is listed in the Supporting Information S3 Text. It is assumed that all the parameters are random variables and are independent of each other. Thus, the joint posterior distribution of the model parameters in the parameter set **Θ**_*i*_, *i* = 1…4 can be determined by Eq (31).

$$\begin{array}{c} \begin{array}{cc} \pi (\Theta_{i}|d_{i}) & \propto L_{i}\left(d_{i}|\Theta_{i},\sigma_{i}\right)\pi (\Theta_{i})\pi \left(\sigma_{i}\right)\\ & \propto L_{i}\left(d_{i}|\Theta_{i},\sigma_{i}\right)\prod_{k=1}^{m_{i}}\pi \left(\theta_{ik}\right)\prod_{j=1}^{n_{i}}\pi \left(\sigma_{ij}\right) \end{array} \end{array}$$ (31)

where *m*_*i*_ represents the number of parameters in **Θ**_***i***_. We used the robust adaptive Metropolis algorithm(RAM) [64] as the MCMC technique. The mean values of the estimated parameters together with the credible regions for each parameter are listed in Table 1. The histograms of the estimated posterior distributions for the model parameters are provided in the Supporting Information S3 Text.

**Table 1:** Parameter estimates for PK and cellular model.

Parameter	Unit	Posterior mean(*q*_0.25_,*q*_0.75_)	Parameter	Unit	Posterior mean(*q*_0.25_,*q*_0.75_)
**PK model**			**Cellular model**		
*V*_max_	*μ* g⋅mL^−1^ ⋅min^−1^	329.539(313.795,346.084)	**TS inhibition**		
*Q*_21_	mL⋅mg^−1^ ⋅min^−1^	320.071(39.926,680.584)	*k*_95_	min^−1^	0.038(0.035,0.043)
*V*_1_	mL⋅ (mg tissue)^−1^	16823.472(15776.621,18123.180)	*k*_59_	min^−1^	2.882(2.877,2.888)
*V*_2_	mL⋅ (mg tissue)^−1^	2999.821(2715.355,3266.188)	*k*_09_	min^−1^	0.152(0.138,0.171)
*K*_*m*_	*μ* g⋅ mL^−1^	17334.977(16261.304,18220.139)	*K*_*dUMP*_	a	21.935(21.932,21.938)
**Cellular model**			*G*_0_	mg⋅min^−1^	0.155(0.131,0.185)
**5-FU in interstitial fluid and its anabolism**			*k*_cat_	min^−1^	17.430(17.419,17.444)
*Q*_31_	mL⋅mg^−1^ ⋅min^−1^	0.981(0.915,1.033)	*k*_08_	min^−1^	0.032(0.021,0.044)
*V*_max,influx_	pmol⋅mg^−1^ ⋅min^−1^	538.451(537.306,539.436)	*α*_*TS*_	a	2.021
*K*_m,influx_	mL⋅ (mg tissue)^−1^	171.153(170.931,171.378)	*k*_*d*_	a	1.034
*V*_max,efflux_	pmol⋅mg^−1^ ⋅min^−1^	536.642(536.044,537.006)	*TS*_0_	pmol⋅mg^−1^	1.860e-2
*K*_m,efflux_	pmol⋅mg^−1^	6.704(6.571,6.829)	$\sigma_{TS}^{2}$	b	0.203(0.199,0.207)
*k*_03_	min^−1^	135.995(134.929,137.845)	$\sigma_{dUMP}^{2}$	b	2(1.9999,2.0001)
*V*_max,54_	pmol⋅mg^−1^ ⋅min^−1^	27.138(26.149,28.348)	**dNTP pool imbalance**		
*K*_m,54_	pmol⋅mg^−1^	2325.154(2324.075,2326.215)	*k*_1_	a	0.213(0.211,0.215)
**5-FU incorporation into DNA and RNA**			*k*_2_	a	32.50(32.49,32.51)
*k*_65_	min^−1^	0.089(0.082,0.096)	*k*_3_	a	3.713(3.711,3.714)
*k*_56_	min^−1^	0.521(0.518,0.525)	*k*_4_	a	73.389(73.368,73.409)
*k*_06_	min^−1^	0.114(0.111,0.119)	*k*_5_	a	3.695(3.682,3.705)
*γ*_lag,RNA_	a	0.310(0.299,0.326)	*k*_6_	a	0.303(0.302,0.305)
*V*_max,75_	pmol⋅mg^−1^ ⋅min^−1^	0.0199(0.0171,0.0223)	*k*_7_	a	3.339(3.334,3.345)
*K*_m,75_	pmol⋅mg^−1^	0.8398(0.8344,0.8467)	*k*_8_	a	1.382(1.375,1.387)
*V*_max,57_	pmol⋅mg^−1^ ⋅min^−1^	5.534(5.525,5.542)	*k*_9_	a	0.27672(0.27663,0.27684)
*K*_m,57_	pmol⋅ mg^−1^	0.95908(0.95894,0.95922)	*k*_*B*_	a	35.65(35.63,35.67)
*k*_07_	min^−1^	0.808(0.796,0.819)	*k*_*A*_	a	6.038(6.022,6.054)
*γ*_lag,DNA_	a	0.554(0.549,0.560)	*k*_10_	a	0.0794(0.0789,0.08)
*T*_*d*,*RNA*_	day	0.396(0.363,0.428)	*γ*_dNTP_	a	1.500e-1
*T*_*d*,*DNA*_	day	4.360(4.344,4.378)	$\sigma_{dNTP}^{2}$	b	0.013(0.0068,0.0158)
$\sigma_{intra}^{2}$	b	199.9999(199.9888,200.0107)	**DSB induction**		
$\sigma_{FNUC}^{2}$	b	200.004(199.974,200.033)	*V*_max,*dNTP*_	min^−1^	10.516(10.515,10.519)
$\sigma_{DNA}^{2}$	b	2.009(1.996,2.022)	*V*_max,*HR*_	Thousands count/min	10.082(10.072,10.087)
			*K*_*m*,*HR*_	Thoudands count	194.020(194.014,194.028)
			*k*_*i*_	min^−1^	0.121(0.112,0.129)
			*k*_0_	min^−1^	2.803(2.789,2.815)
			*γ*_*DSB*_	a	0.6
			*T*_*d*,*DSB*_	Day	1.801(1.800,1.802)
			$\sigma_{DSB}^{2}$	b	199.9999(199.9995,200.0003)

a: unitless parameter

b: variance of the measurement noise.

## Results

### Model construction

#### PK model

**Fig 2:**
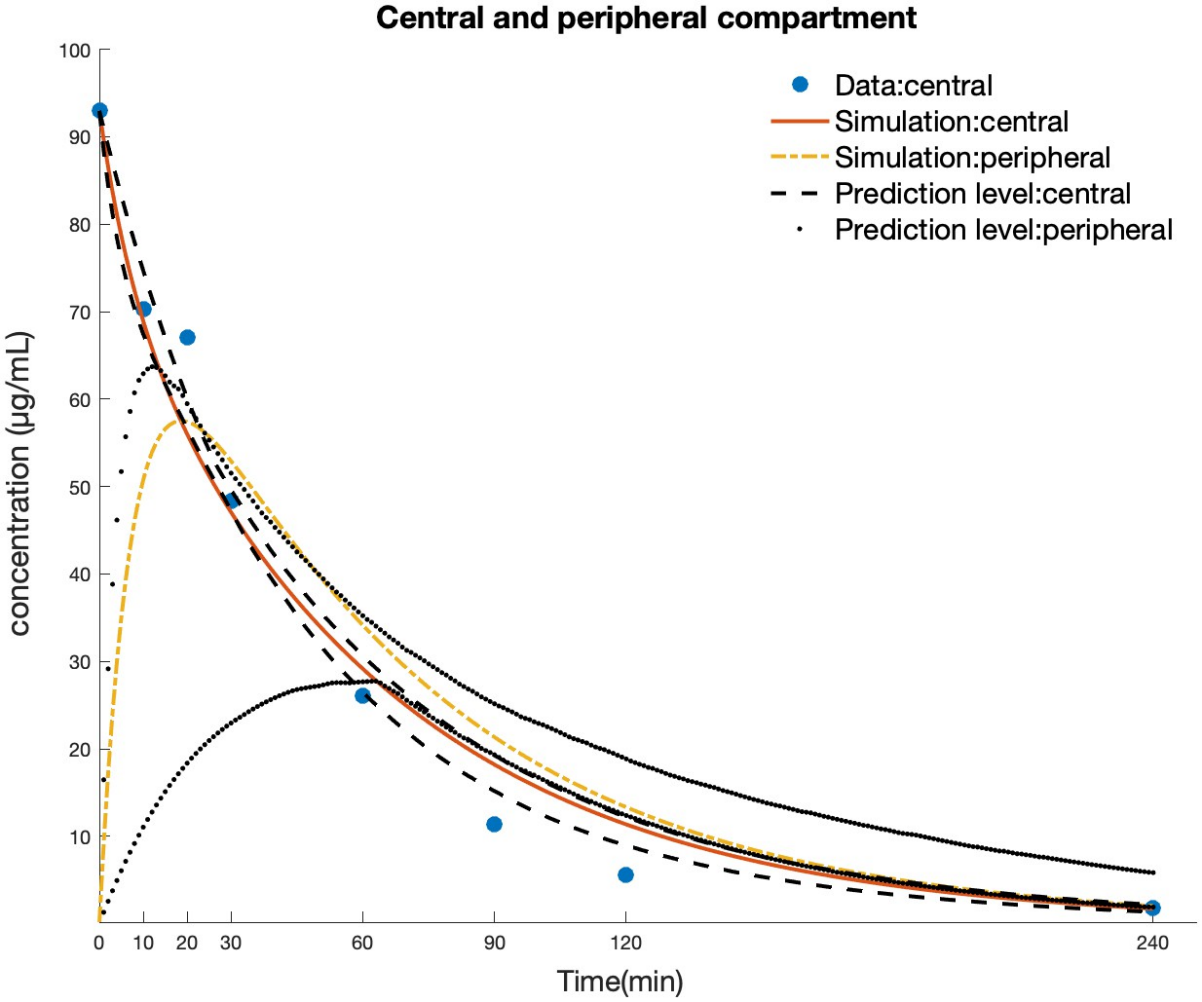
Model simulation of 5-FU concentration in central and peripheral compartment. Blue solid circle represents literature data; red solid curve, time profile of 5-FU in central plasma; yellow dash-dotted curve, the time profile of 5-FU in peripheral compartment. black dashed line: 0.7 credible regions of model simulation of central compartment; black dotted line: 0.7 credible regions of model simulation of peripheral compartment.

The PK model is composed of central and peripheral compartments. The parameters in the PK model are determined by fitting the model to the plasma concentration data measured on animal models injected with a single 100 mg/kg 5-FU dose [65]. To maintain unit consistency throughout the integrated model, the unit of 5-FU plasma concentration is converted to a molarity concentration unit *pmol*/*mL* from *μg*/*mL* via the molar mass of 5-FU(130.077 g/mol). The time course of 5-FU in the peripheral compartment is estimated from the PK data. The parameter estimates are shown in Table 1 and simulated concentration-time curves for compartments 1 and 2 are shown in Fig 2. From the result of parameter estimation, the estimated volume of the central compartment is smaller than that of the peripheral compartment, which agrees with the estimation results shown in the previous PK/PD study [20]. The elimination phase of the peripheral compartment is slower than that of the central compartment.

#### Cellular model

The cellular model is composed of 5-FU concentration in interstitial fluid (Eq (4)), intra-cellular 5-FU(Eq (5)) formation of 5-FU anabolites (Eq (6), insertion of FUTP, FdUTP into RNA and DNA (Eqs (7) and (8)), binding of FdUMP to TS (Eq (9)) and dUMP (Eq (14)), perturbed dNTP pool caused by reduced TS catalytic activity (Eq (18)) and DSB induction (Eq (19)), whose trajectories are expressed by the solutions of the differential equations. The parameters in the cellular model are estimated by fitting to data measured on colon xenograft tumors. The literature data sources are enumerated in Table 2 and the parameter estimates are provided in Table 1. The initial values of the variables are derived from literature data, that is the content of species at 0 h upon exposure to 5-FU. Instead of fitting to literature deta, the time course of *A*_3_(*t*) is estimated dependent on the fluxes between compartment 3 and the neighboring measurable quantities.

**Table 2:** Literature data for the model variables.

Moleuclar species	Model variable	Data source	Experiment	Dose	Route of adiministration
5-FU in plasma	*A*_1_(*t*)	[65]	in vivio, C57/BL6 mice bearing colon 38 tumors	100 mg/kg	IP injection
intracellular 5-FU	*A*_3_(*t*)	[66]	in vivo, Female C57BL/6 mice 8 bearing C38 murine colon tumor	115 mg/kg.	IP injection
5-FU anabolites	*A*_4_(*t*)	[66]	in vivo, Female C57BL/6 mice 8 bearing C38 murine colon tumor	115 mg/kg.	IP injection
FUTP incorpoartion into RNA	*A*_6_(*t*)	[67]	in vivo, Female BALB/c mice bearing colon 26-B tumor	80 mg/kg	IP injection
FdUTP incorporation into DNA	*A*_7_(*t*)	[68]	in vitro, Colon26-B tumors	80 mg/kg	-
TS-dUMP	*A*_9_(*t*)	[57]	in vivo, Male and female Wistar rats bearing dimethylhydrazine (DMH)-induced colon carcinoma	100 mg/kg	IP injection
Total TS	*TS*_*total*_	[57]	in vivo, Male and female Wistar rats bearing dimethylhydrazine (DMH)-induced colon carcinoma	100 mg/kg	IP injection
dUMP	*A*_9_(*t*)	[69]	in vivo, BALB/c mice bearing colon tumor 06/A	80 mg/kg	IP injection
dNTP pool	*Nuc*_*p*_(*t*)	[70]	in vitro, L5178 lymphoma cells	1 *μ*M	-
DSB	*N*_*DSB*_(*t*)	[61]	in vitro, myeloid leukemia (OCI-AML2) cells	3*μmol*/L	-

a: unitless parameter

b: variance of the measurement noise.

**Fig 3:**
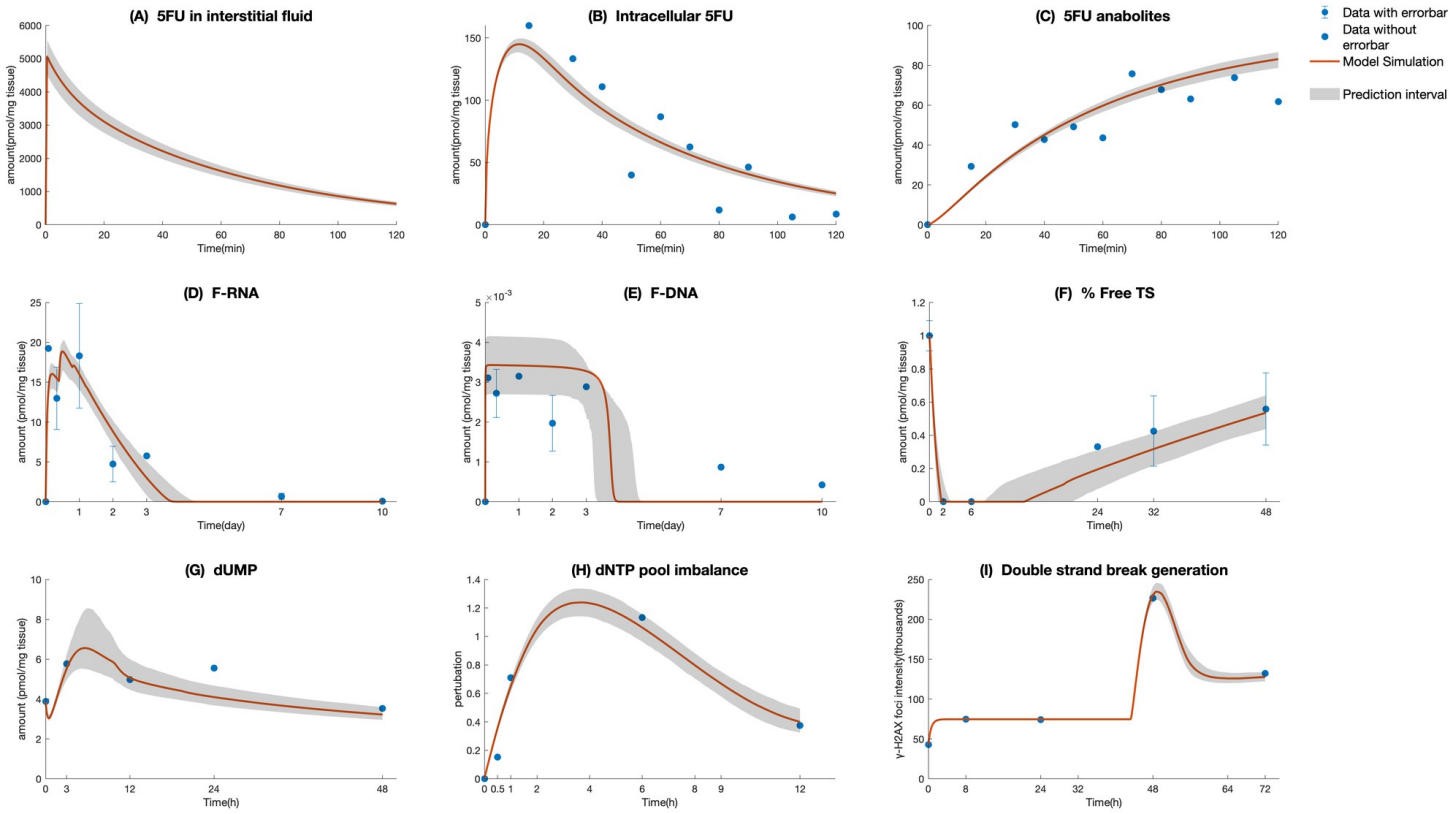
Time profiles of intermediate components in cellular model. (A) 5-FU in interstitial fluid, (B) intra-cellular 5-FU, (C) 5-FU anabolites, (D) F-RNA, (E) F-DNA, (F) % Free TS, (G) dUMP, (H) dNTP pool imbalance, (I) double-strand break generation. Red curve, model simulation; solid blue circle, literature data points; and error bar represents standard errors. The shaded areas represent the 0.7 credible regions.

As shown in the model simulations (Fig 3C), the accumulation of 5-FU anabolites begins within minutes upon exposure to 5-FU, subsequent to the transmission of 5-FU from interstitial fluid to intra-cellular space. The model is able to capture the sharp decrease of non FdUMP bound TS enzyme in parallel to a rapid increase in dUMP, indicating pronounced inhibition of TS activity. In Fig 3F and 3G, it can be observed that the peak of dUMP amount and the lowest level of free TS enzyme coincide at roughly 5 hours after 5-FU administration, after which the decline in dUMP is accompanied by the restoration of free TS. In terms of 5-FU incorporation into the DNA, as demonstrated in both model simulation (Fig 3D and 3E) and literature data, the fraction of FU-RNA is higher than that of FU-DNA. Such a phenomenon may be related to the fact that RNA replication happens all the time in the cell while DNA replication only happens in the S phase. In addition, the duration of incorporation of 5-FU in RNA and DNA is longer than that of other substances, which can be explained by the involvement of cell cycle kinetics in regulating the genomic activity. Fig 3I depicting the formation of DNA lesions caused by 5-FU shows that in the early stages following the 5-FU administration, DSBs are maintained at a certain level before their count noticeably increase after approximately 40 hours and ultimately reaches an equilibrium state. In comparison with the timing of onset of other species, the appearance of 5-FU-induced DNA fragmentation is delayed, because 5FU can only exert anti-tumor effects when DNA replication takes place. By visually comparing Fig 3G and 3H, we also noticed that the maximum values of dNTP pool imbalance and 5-FU-induced DSB are reached asynchronously, in terms with the observations shown in the experimental studies focusing on the impacts of dNTP pools on the generation of lethal DNA damage and cell fate [37]. However, the time elapsed between the peak of dNTP pool imbalance and the generation of 5-FU-initiated DSB may be different if the model is fit to distinct colon cell lines with differential growth pattern and initial cell cycle phase. The histograms of the cellular model parameters are given in the Supporting Information S3 Text. We classified the parameters into identifiable and unidentifiable groups according to the compactness of the supports and shapes of their distributions. As shown in Fig B and Fig C in Supporting Information S3 Text, most of the parameters apart from *Q*_31_, *K*_*m*,*influx*_, *k*_63_, *γ*_*lag*,*DNA*_, *T*_*d*,*DNA*_, *k*_95_, *k*_59_, *G*_0_, *k*_*cat*_, *k*_3_, *k*_4_, *k*_5_, *k*_*B*_, and *k*_*A*_, *k*_*i*_ are identifiable.

#### Tumor growth inhibition model

**Fig 4:**
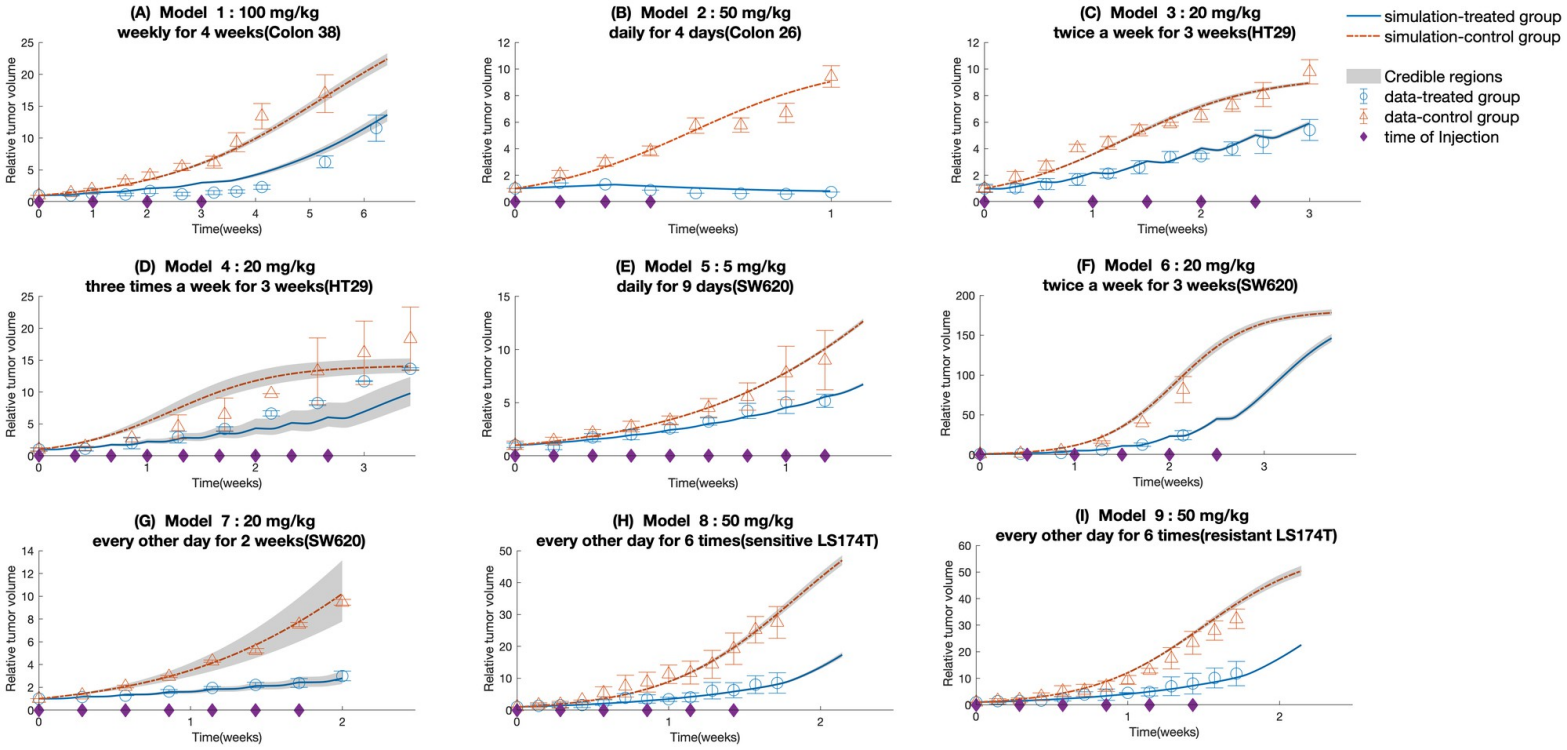
Time-dependent outputs of the 9 TGI model using parameter estimates shown in Table 4. Red line represents the time course of the control group; blue line represents the time course of colon tumor growth treated with corresponding dosage regimens shown in Table 3; triangle, literature data for the control group; circle, literature data for the treated group; error bar, standard errors; asterisk on x-axis represents injection time. The shaded areas in each panel represent the 0.7 credible intervals.

We applied the mechanistic PK/PD model to the dosage regimens shown in Table 3 to probe the alteration in the dose-related parameters as the changes of doses and injection schedules. The parameters in PK and cellular model are kept the same as the ones obtained from colon xenograft tumors given a single 100 mg/kg 5-FU. The initial values of 5-FU in central plasma are varied according to the dosing schemes. The PK model is reloaded at the frequency of dose administration. The parameters describing the kinetics of control groups are estimated for all the dose regimes whose data are from various sources in the literature, keeping the same value for *γ*_*Tv*_. The Fig 4 demonstrates that the model simulations using the mean parameter estimates shown in Table 4 are consistent with the tumor volume dynamics in literature data. Moreover, due to the lack of repeated experimental measurements from the plasma-treated (PBS) control groups, the zero net growth rate around the maximum level is assumed, indicating that the model parameterization could not explain tumor growth above the equilibrium. As the drug effect wears off, the tumor volume would eventually approach an asymptote equal to *P*_max_, the value of which is directly estimated from the time series data with the limited duration. For dosage regimens (1), (2), (3), (5), the parameters in the TGI model are estimated by fitting the literature data, forming models (1), (2), (3), (5). Following this, the parameter estimates for the TGI model 3 are reused on protocols (4), (6), and (7) except for the drug-irrelevant parameters, considering that they have the same amount of single dose and similar dosing schedules. The estimated value of *T*_*d*,*Tv*_ from model (3) is also applied to models (8) and (9). On the other hand, the estimation of TGI parameters except for *T*_*d*,*Tv*_ for the protocols (8) and (9) are carried out separately due to their unique dosing structures and type of cell line. Besides, the parameters governing normal tumor growth pattern (i.e. λ_*d*_, *P*_max_, λ_*g*_) are estimated for each protocol since the experimental data for each protocol is from a different study in the literature, and is subject to the characteristics of the cell lines and progression phases of the xenograft tumors. The control group data is used to characterize the parameters λ_*d*_, *P*_max_, λ_*g*_. The literature tumor volume data are normalized to the respective starting values. It is also assumed that for each model, the first doses are injected at 0 h after the treatment course begins.

**Table 3:** The nine different dosage regimens that are evaluated in our study.

TGI model	Dosage regimen	Cell line	Literature
(1)	100mg/kg, weekly for 4 weeks	Colon 38	[71]
(2)	50 mg/kg, daily for 4 days	Colon 26	[72]
(3)	20 mg/kg, twice a week for 3 weeks	HT 29	[73]
(4)	20 mg/kg, three times a week for 3 weeks	HT 29	[74]
(5)	5 mg/kg, daily for 9 days	SW620	[75]
(6)	20 mg/kg, twice a week for 3 weeks	SW620	[76]
(7)	20 mg/kg, every other day for 2 weeks	SW620	[77]
(8)	50 mg/kg, every other day for 6 times	sensitive LS174T	[78]
(9)	50 mg/kg, every other day for 6 times	resistant LS174T	[78]

**Table 4:** Parameter estimates for the nine TGI models.

	*T*_d,Tv_, min	*IC*_50_, pmol ⋅ *mg*^−1^	*E*_max,*damage*_, units	*EC*_50,*damage*_, units	λ_*g*_ min^−1^	*P*_max_, cm^−3^	λ_d_, min^−1^	$\sigma_{treated}^{2}$	$\sigma_{control}^{2}$	*γ*_Tv_
Model 1	4.9738e2 (4.9736e2,4.9741e2)	7.6262e0 (7.5690e0,7.6779e0)	6.7387e-1 (6.7368e-1,6.7428e-1)	5.1420e0 (5.1326e0,5.1478e0)	7.6643e-5 (7.5381e-5,7.7463e-5)	4.8414e0 (4.8295e0,4.8492e0)	1.1564e-5 (1.0181e-5,1.3015e-5)	2.2878e-2 (1.2150e-2,2.7278e-2)	3.5396e-2 (1.6802e-2,4.4669e-2)	a
Model 2	5.8137e2 (5.8136e2,5.8137e2)	1.4192e0 (1.4170e0,1.4220e0)	2.3961e1 (2.3952e1,2.3973e1)	1.6143e2 (1.6141e2,1.6146e2)	4.2827e-4 (4.2824e-4,4.2829e-4)	1.4204e0 (1.4202e0,1.4204e0)	2.7973e-5 (2.7790e-5,2.8156e-5)	2.0999e-3 (9.2526e-4,2.5272e-3)	1.6150e-1 (1.5918e-1,1.6449e-1)	a
Model 3	8.9345e2 (8.9342e2,8.9347e2)	3.7658e-3 (3.6944e-3,3.8767e-3)	1.7511e-1 (1.6683e-1,1.8534e-1)	2.3802e-1 (2.3647e-1,2.4322e-1)	2.1252e-4 (2.1120e-4,2.1388e-4)	1.2468e0 (1.2448e0,1.2502e0)	5.0506e-5 (5.0297e-5,5.0895e-5)	1.8068e0 (1.8022e0,1.8106e0)	5.3202e-3 (3.1314e-3,6.4018e-3)	a
Model 4	b	b	b	b	2.5101e-4 (2.4870e-4,2.5343e-4)	1.7230e0 (1.6809e0,1.7754e0)	4.4318e-5 (3.4239e-5,5.3867e-5)	5.2956e-1 (4.3654e-1,5.9777e-1)	1.4767e-1 (7.2128e-2,1.8219e-1)	a
Model 5	1.2537e3 (1.2537e3,1.2537e3)	6.9275e-1 (6.9099e-1,6.9642e-1)	7.8173e-1 (7.0030e-1,8.4510e-1)	5.7608e-1 (5.3271e-1,6.0047e-1)	2.2528e-4 (2.2398e-4,2.2612e-4)	2.3627e0 (2.3562e0,2.3678e0)	3.1695e-6 (2.9728e-6,3.3098e-6)	6.2889e-4 (3.7498e-4,7.7701e-4)	2.4629e-1 (2.4258e-1,2.5090e-1)	a
Model 6	b	b	b	b	2.8371e-4 (2.7986e-4,2.8864e-4)	1.1623e1 (1.1504e1,1.1794e1)	3.4289e-5 (3.2932e-5,3.6441e-5)	1.0461e-2 (2.5496e-3,1.0932e-2)	1.5163e0 (1.5001e0,1.5331e0)	a
Model 7	b	b	b	b	1.5008e-4 (1.4464e-4,1.5520e-4)	2.6419e0 (2.5477e0,2.7285e0)	1.7707e-5 (1.3445e-5,2.0765e-5)	7.2275e-1 (7.0288e-1,7.4131e-1)	1.0279e-1 (6.6635e-3,2.1899e-1)	a
Model 8	1.1160e3 (1.1160e3,1.1160e3)	8.7883e1 (8.7795e1,8.8040e1)	5.0672e-1 (4.6947e-1,5.4911e-1)	6.2875e1 (6.2721e1,6.2961e1)	2.3163e-4 (2.2959e-4,2.3392e-4)	8.9848e0 (8.9516e0,9.0314e0)	4.0360e-6 (3.2719e-6,4.6727e-6)	1.0464e-2 (6.5112e-3,1.2935e-2)	5.0422e-2 (3.6380e-2,6.1261e-2)	a
Model 9	1.3002e3 (1.3002e3,1.3003e3)	7.2201e1 (7.2142e1,7.2265e1)	1.5250e0 (1.4508e0,1.5971e0)	4.9656e1 (4.9637e1,4.9670e1)	2.7370e-4 (2.7162e-4,2.7565e-4)	7.5644e0 (7.3151e0,7.7718e0)	5.5131e-6 (4.8012e-6,5.7914e-6)	1.3928e-2 (8.9215e-3,1.6304e-2)	8.6216e-1 (8.3173e-1,8.8676e-1)	a

a: Fixed as 0.2

b: Simulation using parameter estimates obtained from HT29 xenograft tumor under dose regimen (3) (Model 3)

On Table 4, the 0.5 credible interval for each parameter is shown inside the brackets. The numbers of samples used for calculating the empirical mean and associated credible intervals for all the TGI models are 8001, 5101, 10202, 4952, 4801, 7202, 1150, 10501, 9512 respectively.

The values of parameters shown in Table 4 also agree with the extent of adverse effects of the studied dosage regimens. Among all the nine models, the estimated value of *IC*_50_ for model (1) is the largest. It is partially due to more anabolites that are synthesized from a higher level of drug exposure to tumor cells. The DSB-related parameters *E*_max_, *EC*_50,*damage*_ of model (2) are the highest, which is caused by the acute accumulation of DNA lesions as a result of daily administration. As for the mild dose schedule featuring models (1) and (3), the amount of single dose plays a more dominant role in determining the levels of *E*_max_ and *EC*_50,*damage*_, which is indicated by the larger value of the two model parameters for injection of 100 mg/kg 5-FU than that of 20 mg/kg 5-FU. The values of those growth-related parameters are subject to the characteristics of the cell lines and progression phases of the specific xenograft tumors. Overall, the parameter estimates of 9 xenograft tumor models examined in this study fall in the same order of magnitude. Besides, the computational analysis of 5-FU anabolites-effect relationship(*E*_*a*_(*t*)) and DSB-effect relationship (*E*_*DSB*_(*t*)) are documented in Supporting Information S1 Text. Fig D—Fig L in Supporting Information S3 Text show the histograms for the estimated TGI model parameters. The identifiable and unidentifiable parameters for the TGI models (1)—(9) are listed on the figure captions in Supporting Information S3 Text. We conducted a global sensitivity analysis where we first used the Morris method to screen the parameters that have more influence on the model outputs. Then the variance-based global sensitivity analysis, often referred to as Sobol method, is conducted based on the selected parameters. The Morris method revelaed that the changes in the parameters *K*_*m*_, *K*_*m*,*efflux*_, *K*_*m*,54_, *K*_*m*,*influx*_, *P*_*max*_, *V*_*max*,*efflux*_, *V*_*max*,54_, *V*_*max*,75_, *V*_*max*,*influx*_, *V*_*max*_, λ_*d*_, λ_*g*_, *k*_03_, *k*_56_, *k*_59_, *k*_95_, *k*_65_ created considerable perturbations on the model output and these were chosen to be analyzed using the Sobol analysis. The detailed discussion of the analysis and the results are provided in Supporting Information S2 Text. We further used the Sobol global sensitivity to identify the degree of the changes in the model outputs produced by the joint interactions of the input parameters. The first order and total order Sobol index of each parameter with the tumor volume at 5th, 25th and 45 day after treatment begins are given in Supporting Information S2 Text. The results show that model outputs at 45 th day are more sensitive to λ_*g*_, *P*_max_, *V*_*max*,*efflux*_, and *V*_*max*,*influx*_ than the other input factors, which is consistent with the result of preliminary screening by Morris method.

### Quantitative evaluation of treatment efficacy using established TGI models

We further examined extensively the effect of dosage regimens on cytotoxic effects of 5-FU by looking at the area under the curves of *E*_*a*_(*t*) and *E*_*DSB*_(*t*) (denoted as *AUC*_*e*,*a*_ and *AUC*_e,damage_ respectively), the area under the time profiles of *A*_5_(*t*) and *N*_*DSB*,*deviation*_ (denoted as *AUC*_*a*_ and *AUC*_*DSB*_ respectively), the trajectories of *E*_*a*_(*t*) and *E*_*DSB*_(*t*) (shown in S1 Text), and tumor responses (Fig 4). The numerical results of *AUC*_*e*,*a*_, *AUC*_e,damage_, *AUC*_*DSB*_, and *AUC*_*a*_ are shown in Table 5. *AUC*_*e*,*a*_ and *AUC*_e,damage_ are used as the indicators of the cumulative cytotoxic effect of the drug and can be used to predict cumulative tumor responses. Following the model design, *AUC*_e,damage_ increases with the accumulation of drug exposure, while *AUC*_e,a_ experiences the opposite trend. For the protocols (1), (3), (4), (6), (7), their accumulative doses over a week are similar, so is the accumulative effect of *E*_*a*_(*t*) and *E*_*DSB*_(*t*). But by close comparison of the individual *AUC*_e,damage_, the DNA damage caused by 100 mg/kg weekly injection exerts more significant anti-tumor effects among those regimens. And the treatment with 20 mg/kg 5-FU every other day (model (7)) stands out with the smallest *AUC*_e,a_. Furthermore, through the comparison of the AUC measures calculated for the protocol (2) and protocol (8), we can observe that the moderate dose coupled with the intense dose frequency can lead to striking anti-tumor effects. The *AUC*_*DSB*_ and *AUC*_*a*_ corresponding to daily 50 mg/kg 5-FU treatment are the extremest in comparison with those of other schemes. Likewise, the associated trajectory of the relative tumor volume change also demonstrates that daily administration of 50 mg/kg 5-FU for 4 times shows more noticeable cytotoxic effects and causes the tumor shrinkage within the first week after the treatment begins. Such phenomenon can be explained by the observation that the intracellular drug action mediators with extensive amounts resulted from frequent injections are present and continue to take effect over a protracted period of time. The simulation results of models (3), (4), (7) and the corresponding calculated *AUC*_*e*_ show that given the same size of dose and period of treatment, the more frequent drug injection results in the more eminent drug efficacy. It is also worth looking at the time profiles of *E*_*DSB*_(*t*) and *E*_*a*_(*t*) for model (1) to assess the combinations of high dose and less frequent schedule. It can be observed that while the drug effects would fade away during the dose intervals, high doses with a weekly schedule can still yield drastic tumor suppression.

**Table 5:** AUC and AUCe for 9 TGI models.

Parameters	Model 1	Model 2	Model 3	Model 4	Model 5	Model 6	Model 7	Model 8	Model 9
${\text{AUC}}_{e,a}^{\;a}$	4.7900	2.3128	4.9206	3.8823	5.0412	4.9206	3.1336	3.9429	3.8757
${\text{AUC}}_{e,DSB}^{\;a}$	1.2874	33.1088	0.3889	0.4494	1.7674	0.3895	0.4631	0.7832	2.4385
${\text{AUC}}_{a}^{\;a}$	162.4190	396.7319	17.2567	25.8850	4.2039	17.2567	34.5052	194.0453	194.0453
${\text{AUC}}_{DSB}^{\;a}$	2.8343	4.0616	0.9044	1.1920	0.6292	0.9044	1.3578	3.6296	3.6296

a: Calculated by integrating the time courses over a week

*AUC*_*e*,*a*_ and *AUC*_e,damage_ are calculated by integration of the profile of *E*_*a*_(*t*) and *E*_*DSB*_(*t*) over a week respectively. AUC_*a*_ and AUC_*DSB*_, calculated in the same way as *AUC*_*e*,*a*_ and *AUC*_e,damage_, are the area under the time profile of normalized count of DSB(*N*_*DSB*,*deviation*_) and the amount of 5-FU anabolites (*A*_5_(*t*)).

Taken together, 5-FU suppresses cell proliferation of colon cancer in a time- and dose-dependent manner and the accumulative PK exposure can improve the overall therapeutic efficacy. The treatment with daily injection of 50 mg/kg 5-FU four times exhibits the most detrimental effect on tumor burden. A high-dose 5-FU taken over long period may produce significant anti-tumor effects. Regarding the same doses given over the same treatment duration at specific intervals, the more intense schedule may increase the likelihood of exposing vulnerable tumor cells to 5-FU and yields a more significant anti-tumor effect.

### Simulation analysis of tumor resistance towards 5-FU exposure

In this section, we investigate various scenarios on the mechanisms underlying the resistance of tumor responses towards 5-FU treatment using numerical simulations. We evaluated simultaneous changes in physiologically-related parameters that would generate a resistant behavior. We summarized the parameter variations we used to identify possible resistance mechanisms on Table 6, which also lists how these variable changes reflect the biologically relevant resistance mechanisms studied in the literature. The last three entries on the table show our novel hypothesis on other possible causes that might lead to treatment resistance. Considering that resistance may be caused by interwoven cellular pathways, we simulated the simultaneous changes of certain factors to examine the aggregate effects. The Monte Carlo method is applied to generate *N*(= 500) parameter sets and time courses of tumor volume change are simulated for each parameter set. Each parameter set comprises the concerned parameters with newly sampled values from their associated sampling intervals(Eq (32)) and the remaining parameters with baseline values. The resultant time courses are examined for their closeness to the resistant tumor response to identify the parameter changes that resulted in a resistant behavior. The baseline values of parameters are the ones defined for PK and cellular model and the TGI model 8 (sensitive LS174T cell line). The dosage regimen adopted by the model simulations for resistance analysis is the one corresponding to the TGI models (8) and (9). The resistant tumor response is assumed to be the simulation of the TGI model (9). The parameters we experimented on in this section are the ones related with 5-FU drug actions and resistance mechanisms, which are V_max,54_, K_m,54_, G_0_, k_cat_, k_08_, k_59_, k_95_, k_09_, k_5,dNTP_, T_*d*,DSB_, *α*_*TS*_, *T*_*d*_, *E*_max,*damage*_, *EC*_50,*damage*_. The simulation scenarios highlighting their combined effects are: (A) Increased TS expression plus reduced production and stability of TS-FdUMP complex, (B) Accumulation of dUMP plus reduced production and reduced stability of TS-FdUMP complex, (C) Decreased accumulation of anabolites plus reduced production and stability of TS-FdUMP complex, (D) Reduced production and stability of TS-FdUMP complex plus delayed production of DNA fragmentation, (E) Effect of the salvage pathway plus delayed production of DNA fragmentation., (F) The delay of production of damaged cells and the effect of the salvage pathway., (G) The reduced effect of DNA fragmentation on the production of damaged cells plus reduced stability of TS-FdUMP complex. As the effects of specific parameters are analyzed, the values of the others in the model are fixed.

$$\begin{array}{c} p_{i}^{l}+\text{rand}\times \left(p_{i}^{u}-p_{i}^{l}\right) \end{array}$$ (32)

Where $p_{i}^{l}$ and $p_{i}^{u}$ are the lower bound and upper bound of parameter *p*_*i*_. rand represents a random number drawn from the standard uniform distribution. The ranges of variation for each parameter are shown in Table 6.

**Table 6:** The range of variation for each parameter and their corresponding biological implication.

Table 5: The range of variation for each parameter along with the underlying biological implication			
Parameter	Upper bound	Lower bound	Biological implication from literature
V_max,54_	-	2 fold decrease	Decreased accumulation of anabolites [56]; metabolism may predict tumor sensitivity to 5-FU therapy [40]
*K*_*m*,54_	2 fold increase	-	Decreased accumulation of anabolites
G_0_	10 fold increase	10 fold decrease	dUMP accumulation [56]
k_cat_	10 fold increase	10 fold decrease	dUMP accumulation
k_08_	10 fold increase	10 fold decrease	dUMP accumulation
*k*_95,*complex*_	-	10 fold decrease	decreased enzyme affinity for FdUMP [56, 79]
*k*_59,*complex*_	10 fold increase	-	Decreased stability of ternary complex [56, 79]
*k*_09,*complex*_	10 fold increase	-	Decreased stability of ternary complex [56, 79]
k_5,dNTP_		10 fold decrease	The effects of salvage pathway on lessoning dNTP pool imbalance [80].
*T*_d,DSB_	2 fold increase	-	Delayed 5-FU-induced DNA fragmentation and cytotoxicity in the resistant cell lines [79].
*α*_*TS*_	10 fold increase	-	Increased TS protein expression [53, 56]
*T*_*d*_	10 fold increase	-	The delay of transforming the proliferating cells to damaged cells
*E*_max,*damage*_	-	10 fold decrease	The reduced effect of DNA fragmentation on production of damaged cells
*EC*_50,*damage*_	10 fold increase	-	The reduced effect of DNA fragmentation on production of damaged cells

The departure of each tumor growth time course simulated using a new set of parameters from the time course for resistant LS174T tumor cells (model (9)) is calculated as the sum of the Euclidean distances between the coupled points on the two time course trajectories. The time course closest to the insensitive / resistant tumor response is the one with the smallest departure. The mean time course for a certain case is calculated by averaging all the computed time courses. Maximal and minimal time courses among all the simulation results are determined by calculating the *ℓ*_2_-norm of Euclidean distances between the sample points along certain path and x-axis. Table 7 demonstrates parameter variations that can give rise to tumor response closest to the resistant LS174T xenograft tumor(model (9)) for all the seven cases A–G.

**Table 7:** Values and fold change from the baseline of the 11 parameters considered in resistance analysis producing the simulations closest to the time course of resistant LS174T xenograft tumor.

Parameter	Case A	Case B	Case C	Case D	Case E	Case F	Case G
V_max,54_			2.225e+01(1.2 fold ↓)				
*K*_*m*,54_			2.688e+03(1.2 fold ↑)				
G_0_		3.406e-01(2.2 fold ↑)					
k_cat_		5.417e+01(3.1 fold ↑)					
k_08_		6.769e-03(4.7 fold ↓)					
*k*_95,*complex*_	3.434e-02(1.1 fold ↓)	3.432e-02(1.1 fold ↓)	1.178e-02(3.3 fold ↓)	3.289e-02(1.2 fold ↓)			2.410e-02(1.6 fold ↓)
*k*_59,*complex*_	1.810e+01(6.3 fold ↑)	6.175e+00(2.1 fold ↑)	1.853e+01(6.4 fold ↑)	3.515e+00(1.2 fold ↑)			3.119e+00(1.1 fold ↑)
*k*_09,*complex*_	1.235e+00(8.1 fold ↑)	1.479e+00(9.7 fold ↑)	8.614e-01(5.6 fold ↑)	1.257e+00(8.2 fold ↑)			1.342e+00(8.8 fold ↑)
k_5,dNTP_					3.451e+01(9.3 fold ↑)	2.285e+01(6.2 fold ↑)	
T_*d*,DSB_				1.104e+01(6.1 fold ↑)	2.261e+00(1.3 fold ↑)		
*α*_*TS*_	1.037e+01(5.1 fold ↑)						
*T*_*d*_						3.886e+03(2.9 fold ↑)	
*E*_max,*damage*_							6.461e-01(1.3 fold ↓)
*EC*_50,*damage*_							6.872e+02(9.3 fold ↑)

**Fig 5:**
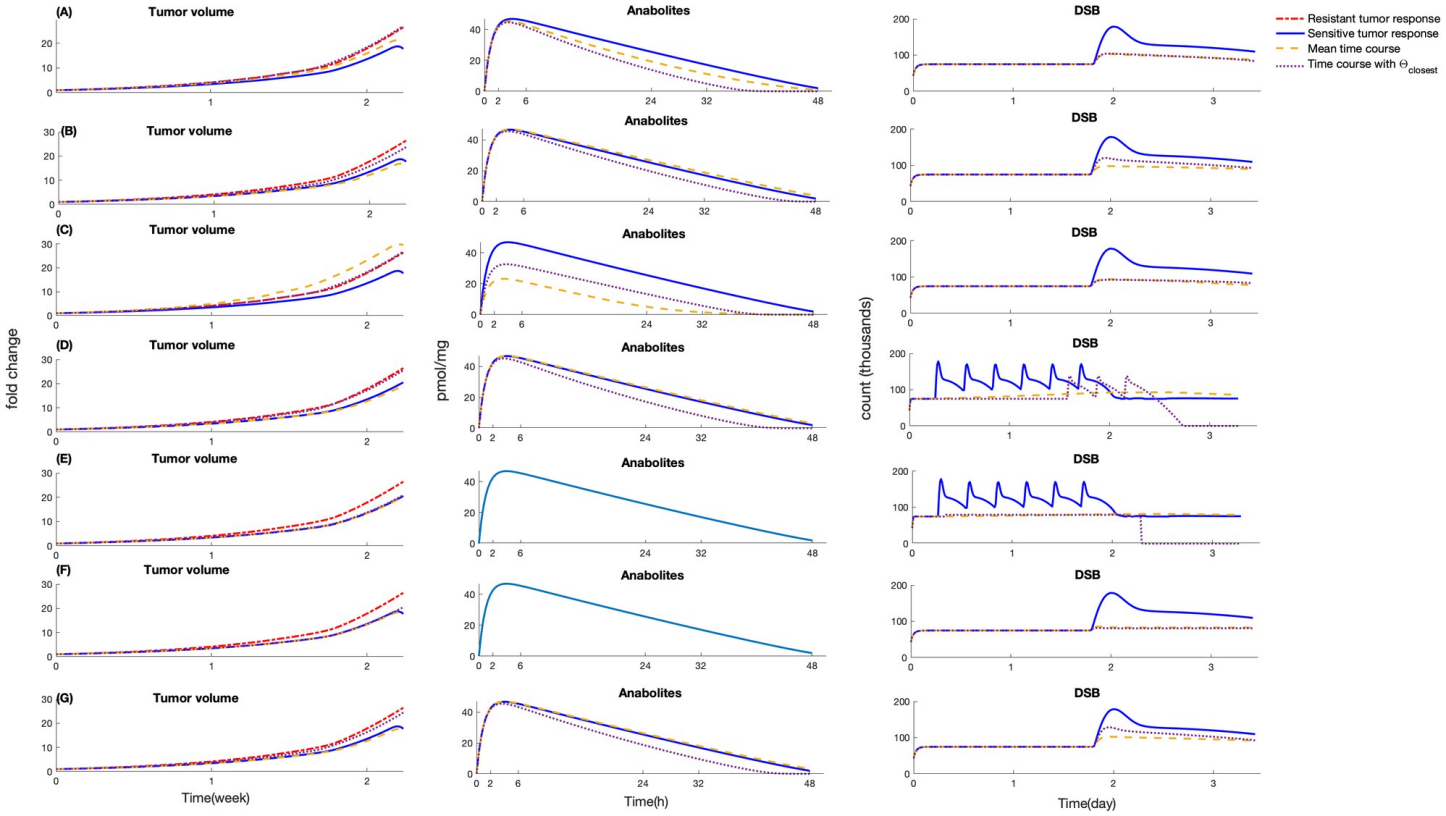
The simulation result of resistance analysis. The first column is the time profiles of tumor volume; second column, 5-FU anabolites, third column: 5-FU induced DSB. Each row represents each aforementioned scenario. Red dash-dotted line, model simulation of resistant LS174T xenograft tumor; blue solid line: model simulation of sensitive LS174T xenograft tumor; yellow dashed line, the mean time course of 500 computed time courses; Θ_closest_ denotes the parameter set associated with the time course of tumor volume closest to the tumor resistance response; purple dotted line: the time course generated by Θ_closest_.

The results that are given in Fig 5 demonstrate that the cases (A), (B), (C), (D), (G) achieve the goal of generating the kinetics of tumor volume close to that of TGI model (9). The treatment response of sensitive LS174T xenograft tumor we chose as the baseline level has changed into a resistant response through the coupling of the decreased accumulation of 5FU anabolites and the change in the kinetics of dUMP, TS-FdUMP complex, 5-FU induced DSB, and tumor volume. Fig 5C shows that the decreased net production rate of anabolites accompanied by the reduced stability of TS-FdUMP complex can lead to the mean resistant response even beyond the reference resistant response. For cases (D)and (E) involving the increase in *T*_*d*,DSB_, the mean time course of 5-FU-induced DSB captures the delaying occurrence of 5-FU induced DSBs and decreased peak values, compared to DSBs induction in the absence of tumor resistance. It can be explained by the depletion of TS-FdUMP complex and strengthened thymidine salvage pathway that reduces the DSB production caused by 5-FU interference, and the salvage pathway with expanded capacity may have more striking effects than decreased FdUMP binding to TS. Among the cases (A), (B), (C), (D) and (G) examining the effect of TS-FdUMP complex related parameters, the highest levels of mean and closest time courses of 5-FU induced DSBs are smaller than the reference level with case (A) and case (C) having the smallest peak values, indicating that the increased TS expression and fewer anabolites may further obstruct the generation of 5-FU induced DSBs. Although the cases (A), (B), (C), (D) and (G) already produce the striking inhibition on DSBs counts, the case (F) leads to the most significant effect by plummeting *N*_*DSB*_ to its baseline. The cases (D) and (G) demonstrate similar resistance responses in terms of the tumor volume and magnitude of 5-FU induced DSBs.

### Generalizability of the mechanism-based model

The generalizability and accuracy of our proposed mechanism-based model on the prediction of tumor responses given a specific dose and dosage regimen are evaluated in this section. We applied different dosing schedules to the TGI model developed above to see how well it can capture the literature data for the target dosing schedules. The values of efficacy-related parameters (i.e. *E*_max_, *EC*_50,*damage*_, *IC*_50_, *T*_d,Tv_) are kept the same for models (3), (4), (6), (7), because these models have the same amount of single dose, implying that the levels of drug-related intermediates are expected to be the same for the single administration. Because the cell line differences have been addressed through control groups, as shown in Table 4, the distinct dose schedules should be the sole drug-regulated factor that drives the differences in the outputs of these models. Therefore, the efficacy-related parameters (i.e. *E*_max_, *EC*_50,*damage*_, *IC*_50_, *T*_d,Tv_,) of models (4), (6) and (7) are kept equivalent to those of model (3).

In order to facilitate the assessment of model generalizability, we organized the TGI models except for models (1), (2), (8) and (9) into three different groups according to the rules defined as follows: (A) models with the same type of xenograft tumor and the same single dose but with different frequencies; (B) models with a different type of xenograft tumor under the same protocols; (C) models with a different type of xenograft tumor under the same amount of dose but different frequencies. Models (3) and (4) fall under scenario A. Models (3) and (6) satisfy the condition of scenario B. The last scenario C involves models (3), (4), (6), (7). Fig 4D indicates that the model 4 simulations for protocol (4) given in Table 3 moderately captures the data, which suggests that the model is structurally stable in the face of changes in the dose frequency. Regarding scenario B, the selections of protocols (3) and (6) from Table 3 show that the differences in treatment responses are caused by the distinction in the types of xenograft tumor. Fig 4C and 4F show that the model simulations agree well with literature data, indicating that our model can adapt to the different tumor types merely through adjusting the parameters for normal tumor growth. Lastly, the experimental measurements of model (3), (4), (6), and (7) suggest that changing dose schedules and types of cell lines influence tumor responses to multiple doses, which parallels the simulations of these four models. The model outcomes of (3), (6) and (7) successfully capture the experimental dynamics, whereas model (4) has a moderate fit to the experimental data. From the generalizability standpoint, model (4) that shares the same efficacy-related parameters was able to capture the general trend in the observed data. Based on the outcomes of evaluation discussed above, it can be concluded that the proposed model constructed under a certain dosing scheme is capable of predicting the tumor responses towards a similar dosing scheme or the one with the same amount of dose but different administration frequency, as long as the control tumor growth has been captured by the model with no drug input.

**Fig 6:**
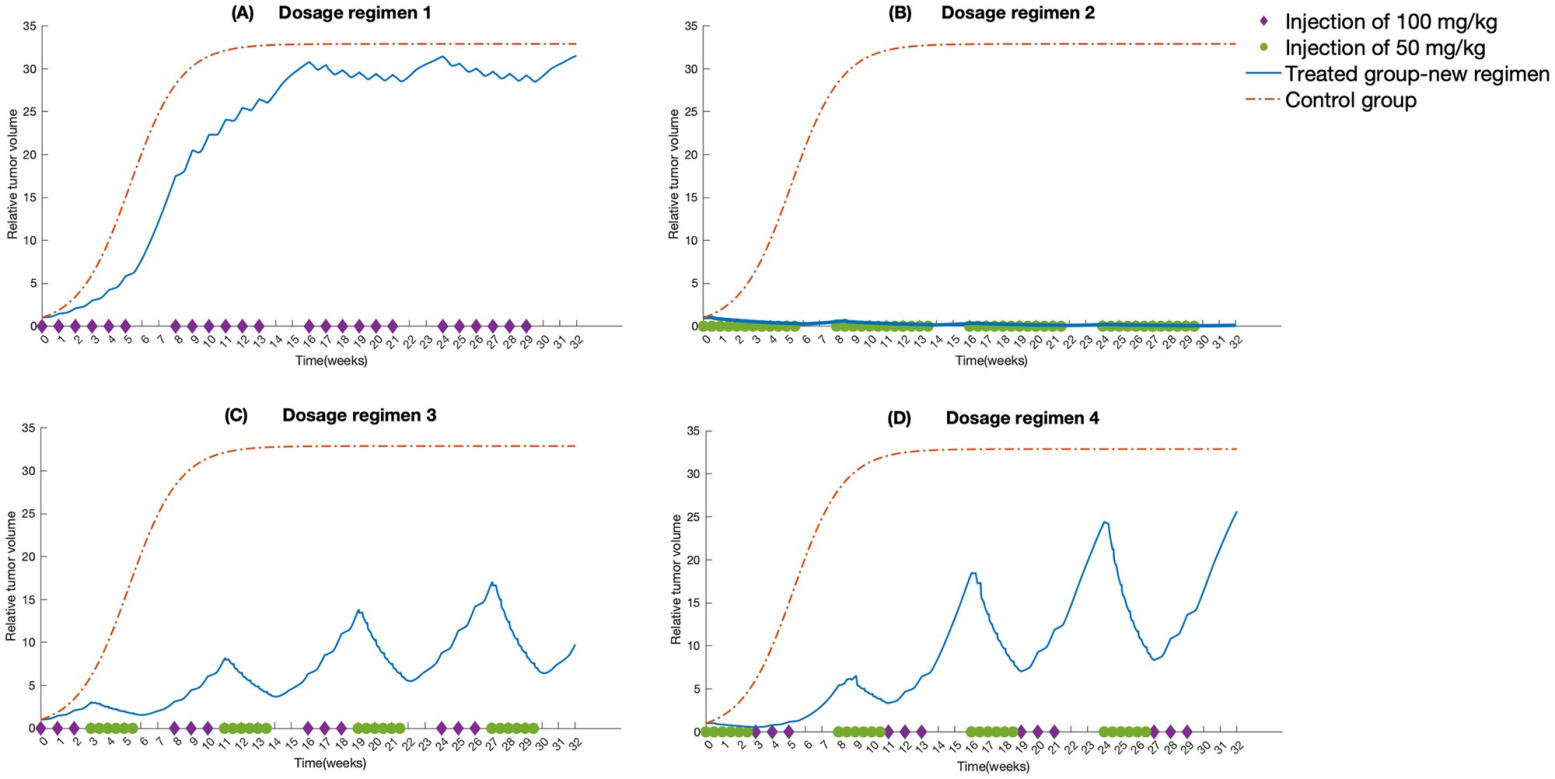
The simulation results associated with the four novel dosage regimens. The regimens under consideration are (A) 100 mg/kg on day 1 of weeks 1 to 6 of a eight-week cycle, for a total of 4 cycles; (B) 50 mg/kg twice a week of weeks 1 to 6 of a eight-week cycle, for a total of 4 cycles; (C) 100 mg/kg on day 1 of weeks 1 to 3 followed by 50 mg/kg twice a week of weeks 4 to 6, for a total of 4 cycles (32 weeks); (D) 50 mg/kg twice a week of weeks 1 to 3 followed by 100 mg/kg on day 1 of weeks 4 to 6, for a total of 4 cycles (32 weeks). Red dash line: model simulation of control; blue solid line: simulation results associated with the four dosage regimens; purple diamond-shaped dot: time of injection of 100 mg/kg 5FU; green round dot: time of injection of 50 mg/kg 5FU.

We utilized our models to computationally experiment with dose regimens that are different than the ones given in Table 3 to further examine the anticancer efficacy of 5-FU treatment. We developed four hypothetical regimens with different combinations of doses, dosing frequencies and treatment durations than the ones we used from the literature. The regimens used for these computational experiments are (A) 100 mg/kg on day 1 of weeks 1 to 6 of an eight-week cycle, for a total of 4 cycles; (B) 50 mg/kg twice a week of weeks 1 to 6 of an eight-week cycle, for a total of 4 cycles; (C) 100 mg/kg on day 1 of weeks 1 to 3 followed by 50 mg/kg twice a week of weeks 4 to 6, for a total of 4 cycles (32 weeks); (D) 50 mg/kg twice a week of weeks 1 to 3 followed by 100 mg/kg on day 1 of weeks 4 to 6, for a total of 4 cycles (32 weeks). The simulation results are shown in Fig 6. Despite having the same total amount of doses over a week, dosage regimens (A) and (B) result in notably different treatment outcomes due to the difference in administration times. The simulation result in Fig 6B shows that within the first cycle, the consecutive twice-a-week injection of 50 mg/kg 5-FU causes a significant tumor shrinkage while the tumor still grows under weekly injection of 100 mg/kg as shown in Fig 6A. Fig 6C and 6D demonstrate that the order of administration of different dosage regimens makes a difference for the treatment options comprising mixed doses. Injection of 50 mg/kg 5-FU twice a week for three weeks following weekly injection of 100 mg/kg 5-FU can inhibit the tumor growth more than the regimen with the opposite order. Computationally, all of the dose regimens presented here show more favorable treatment outcomes compared to the TGI model (1) with the dose regimen 100mg/kg administered weekly for four weeks. Comparison between TGI model(1) and dosing regimen (3) on relative tumor change between three weeks and seven weeks after treatment reveals that shortening the intervals between consecutive lower dosages might yield better outcomes of cytotoxicity. It should be noted that these dose regimens are developed to conduct computational experiments on treatment outcomes and it is necessary to experimentally identify the toxicity profiles and the maximum tolerable doses for the evaluation of dosing schedules before they can be used in a clinical setting. It can be concluded from the computational experiments that applying dosage regimens with high potency after the ones with less potency may result in clinically favorable treatment outcomes.

## Discussion

Mathematical modeling is a powerful tool for capturing the target pharmacology and making predictions on tumor response by translating current understanding towards underlying mechanisms into rigorous mathematical terms. Our physiology-based model allows for the analysis of the dynamics of critical intermediate components related to 5-FU effective drug actions on different time scales and across physical domains. Our cellular model quantitatively reflected the drug effects and related the mechanisms of TS inhibition with disturbance in the dNTP pool and induction of DSBs. The tumor heterogeneity was integrated into the TGI model. The combination of inhibition of proliferating progenitor cells and production of damaged quiescent cells characterized the dynamics of suppressed tumor growth. We converted the time profiles of the 5-FU anabolites and 5-FU-induced DSBs into nonlinear measures of drug effect on inhibiting tumor growth rate and simulating the loss of proliferative cells to provide a realistic prediction of the kinetics of tumor progression under 5-FU treatment. The integrated computational model has the potential to promote the identification of contributions of the physiological intermediates on the clinically favorable responses and potentially enable the precise prediction of medical treatment by antagonistic agents.

Like the other mathematical models, our model is inevitably influenced by a certain level of uncertainty. The uncertainty may stem from the insufficient measurements, parameter estimation, and alternative model structures, which propagate and aggregate as the model progresses. Furthermore, the solution of the dynamic model could be distracted by a local minimum, in the sense that the model is inherently un-convex. This means that we cannot solely depend on the point estimates provided by the optimization scheme involving the minimization of the objective function. Instead, gaining the knowledge of the joint posterior distribution of model parameters is beneficial in terms of recognizing model identifiability. To that end, we implemented the sampling-based Bayesian inference method through the MCMC technique. The model identifiability was examined by plotting the histograms for each parameter in the sample set. In addition, although the time course measurements for system variables are collected from different literature, we attempt to constrain the scope of data to in vivo or in vitro measurements under 100 mg/kg 5-FU treatment.

Examination of the combined effect of doses, dosage regimens and duration of treatment via the established model helps us seek the concealed relationship among the treatment protocols. When simulating different dosage regimens, we adapt to the lack of time-series data of fluorinated compounds under other amounts of drug dose than 100 mg/kg by using the cellular model obtained using 100 mg/kg data collections. This may be due to the fact that the 5-FU anabolites possess a rapid rate of transformation, and capturing them under smaller doses to a precise degree may run into technical difficulties. Therefore, it is unrealistic to reevaluate the parameters of the cellular model for each dosing regimen. We used the cellular model determined by fitting to 100mg/kg literature data to simulate all the aforementioned dose and dose schedules. From the model simulations under multiple dosage regimens, we found that a single high dose outperforms the divided dose in terms of drug efficacy of causing tumor shrinkage. Besides, the more intense schedules are able to yield higher therapeutic efficacy compared to the protocols with the same doses and treatment course but less frequent schedules. Taken together, our model can provide an accurate prediction of pharmacological responses to different dosage regimens. Through such investigation, based on our comprehensive model, it has the potential to advance the quantification of chemotherapy potency and to facilitate the development of efficacy optimization.

We also extended the application of the model to the analysis of tumor resistance responses to the cellular defense mechanisms attributed to tumor insensitivity towards 5-FU, influencing the final treatment outcome. For instance, deoxyuridine triphosphate nucleotidohydrolase (dUTPase), an enzyme catalyzing the degradation of FdUTP to FdUMP, plays a contributory role in enhancing tumor resistance. The experimental study suggests that the cellular (F)dUTP accumulation resulting from dUTPase activity may be related to the insensitivity of tumor to fluoropyrimidine-induced cytotoxicity [79]. Moreover, based on the experiments conducted on mice bearing murine mammary carcinoma, the researchers have found that the incorporation into RNA can be blocked by deoxyuridine (dUrd), rescuing the tumor cells from the lethal toxicity of FUra [81]. Homologous recombination repair (HR) can protect the tumor cells against exogenous insults. DSBs can be repaired by HR with high fidelity [82]. The expression level of the HR proteins is also related to the tumor resistance to the 5-FU anti-tumor effects [60]. In addition, it has been proposed that a slow-down in cell cycle and the reduced expression of CDK2 protein can prevent the incorporation of 5-FU metabolites into DNA [56]. However, all the cellular mechanisms described are more or less relevant to 5-FU -induced TS inhibition since TS is a key player in cellular nucleotide metabolism. Therefore, we conducted the analysis of tumor resistance responses around 5-FU induced TS inhibition and its downstream events, such as the dNTP pool imbalance and DSB induction. As a result of our computational analysis, the decreased accumulation of 5FU anabolites, variations of dUMP from baseline, reduced production of TS-FdUMP complex, and eased pressure from 5-FU induced DSB may bring about the tumor resistance responses.

Taken together, our integrated model forms a nested multi-scale structure and quantitatively connects the effects of 5-FU on tumor growth with genome instability and PK-dependent physiological turnover processes, facilitating simulation studies and resistance analysis. The PK, cellular, and tumor growth inhibition models were arranged in accordance with the mechanistic sequence, providing a realistic description of physiological events that ultimately decide the tumor fate. By fitting to literature data measured from preclinical animal models under different dosage regimens, the proposed model showed robust predictive performance and can simulate the PK and PD profiles under different protocols, reflecting that the model has the potential of being applied in the protocol designing phase. Our proposed semi-mechanistic PK/PD approach provides insight towards translation from xenograft tumor study to clinical efficacy analysis in a computational manner. The model simulations can also provide a tool for researchers to compare and evaluate the impacts of various possible dose and dose frequencies on the improvement of drug efficacy, driving the selection of desirable chemotherapy treatment.

## Supporting information

S1 Text Quantitative analysis of biomeasures *E*_*a*_(*t*) and *E*_*DSB*_(*t*) in TGI model. The file details the computational analysis of 5-FU anabolites-effect relationship(*E*_*a*_(*t*)) and DSB-effect relationship (*E*_*DSB*_(*t*)).
(PDF)

S2 Text Sensitivity analysis. (PDF)

S3 Text Identifiability analysis. (PDF)
