# Supplementary material for: Computational analysis of 5-fluorouracil anti-tumor activity in colon cancer using a mechanistic pharmacokinetic/pharmacodynamic model
Source: PLoS Comput Biol. 2022 Nov 17;18(11):e1010685. doi: 10.1371/journal.pcbi.1010685 (PMC9671373; doi:10.1371/journal.pcbi.1010685)
Supplement: S1 Text — The file details the computational analysis of 5-FU anabolites-effect relationship(Ea(t)) and DSB-effect relationship (EDSB(t)). (PDF) [file pcbi.1010685.s001.pdf]

Quantitative analysis of biomeasures $E_a(t)$ and $E_{DSB}(t)$ in TGI model.

The effect of two mechanistic components 5-FU anabolites($A_5(t)$) and DSB($N_{DSB,deviation}(t)$) are analyzed by plotting the time courses of $E_{DSB}(t)$ (Fig A) and $E_a(t)$ (Fig B) under 9 dosage regimens. The time profiles of the two effect terms are periodic due to the repeated injection. The figures demonstrate that 5-FU anabolites and DSBs exhibit inhibitory effects on tumor growth in a dose-dependent manner. Through the comparison of time courses of $E_{DSB}(t)$ and tumor volume shown in all the panels in Fig A, it can be seen that $E_{DSB}(t)$ explodes towards its highest level following each injection coincides with decreased tumor net growth within the dosing intervals. This followed by a decrease in $E_{DSB}(t)$ to the certain level before replenishing due to the next dose. As for $E_a(t)$, the trend is different because $E_a(t)$ plays a different role on tumor kinetics. Fig B illustrates that the decline in $E_a(t)$ is initiated almost immediately after each bolus injection because of the increase in 5-FU anabolites. Once it reaches the minimal level, $E_a(t)$ will rise rapidly to its baseline, during which tumor cells are returning to their normal growth pattern. Under the withdrawal of the drug, the tumor growth pattern would gradually return to normal and, in the final analysis, reach the equilibrium, the level of which is decided by the associated TGI model without treatment. Physiologically, the rebound of tumor volume may be related to the recovery of the critical proteins and reactivation of cell machineries along with the disappearance of drug effects. The changes in kinetics of $E_{DSB}(t)$ and $E_a(t)$ bring about the changes in tumor kinetics, differentiating the treated group from the untreated group. However, $E_a(t)$ and $E_{DSB}(t)$ for TGI model (2) show distinct patterns from these for the other models(Fig A model 2 and Fig B model 2). Following the rapid decrease, the level of $E_a(t)$ remains within a range between 0.2 and 0.3 with small fluctuations representing the concurrent elimination of 5-FU anabolites between two doses. On the other hand, $E_{DSB}(t)$ shows a noticeable growth shortly before the second dose because the induction of DSBs caused by TS inhibition happens to appear around 48 hours. Similar to $E_a(t)$, $E_{DSB}(t)$ keeps above the level of 7.5 in a protracted period even beyond the treatment duration. Such an extreme phenomenon results from the combination of the moderate size of the dose and intense dose schedule. The short time intervals between doses obstruct the removal of drug effects and bring about the accumulation of 5-FU anabolites and 5-FU induced DSBs, giving rise to tumor growth inhibition.

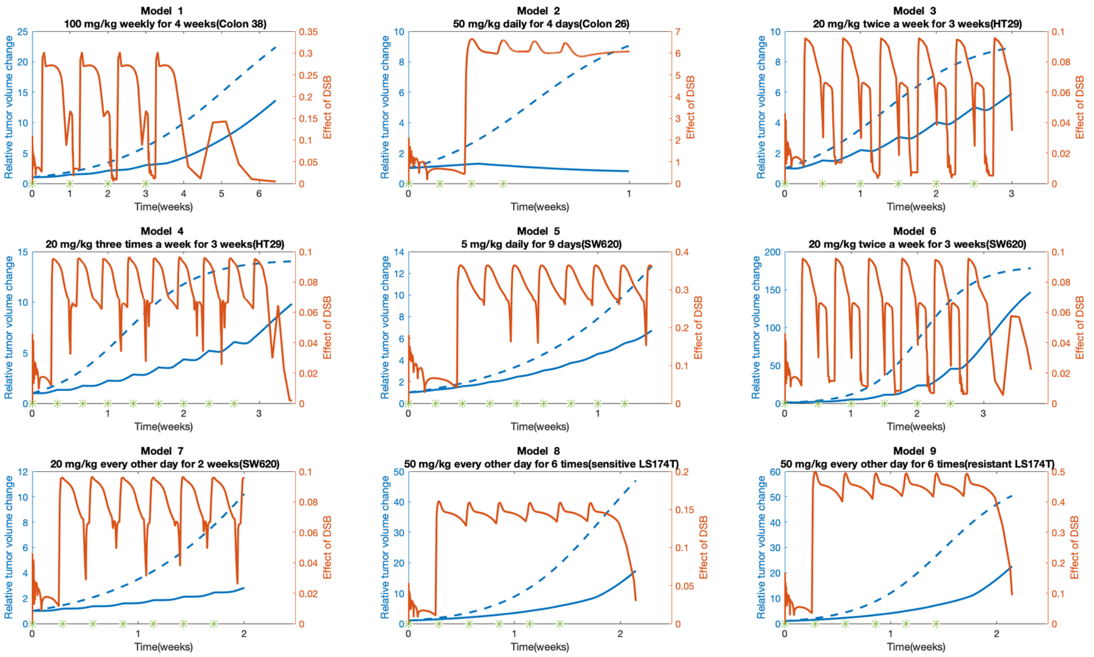

Fig A. Model-predicted time profiles of tumor volume and 5-FU induced DSB effect measure($E_{DSB}(t)$) for all the nine models Red line represents the time course of the control group; the blue line represents the time course of colon tumor growth treated with different dosing regimens; triangle, literature data for the control group; circle, literature data for the treated group; error bar, standard errors; asterisk on the x-axis represents injection time.

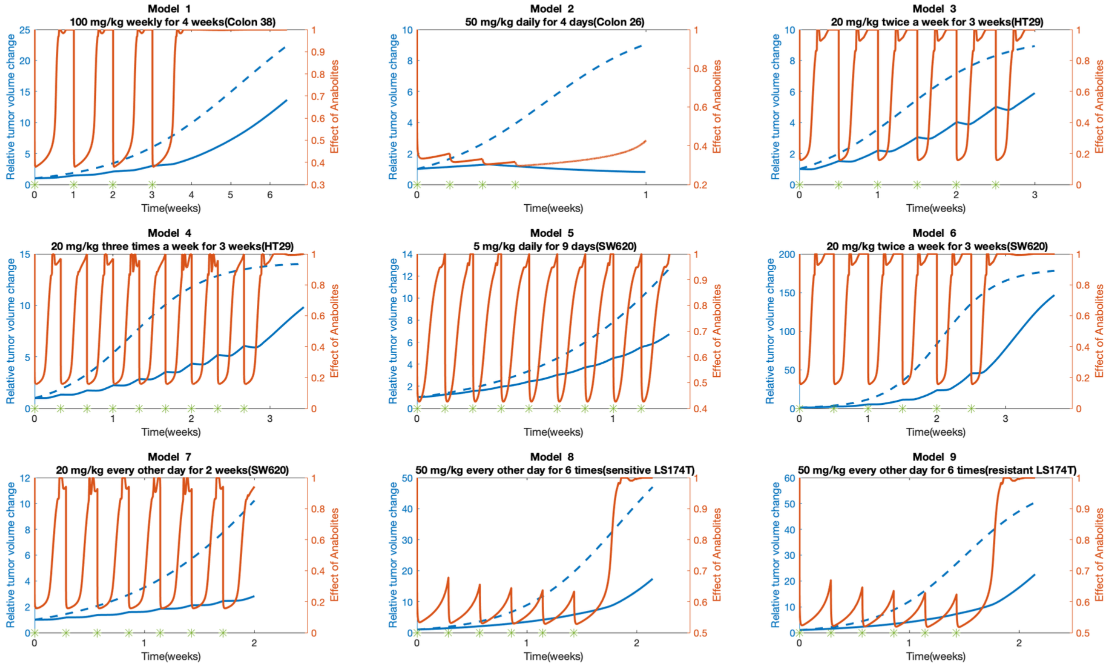

Fig B. Model-predicted time profiles of tumor volume and 5-FU anabolites effect measure ($E_a(t)$) for all the nine models. Dash blue line represents the change of tumor volume of the control group as time; solid blue line, change of tumor volume of the treated group as time; solid orange line, the time course of $E_a(t)$; asterisk on the x-axis represents injection time.
