# Supplementary material for: Computational analysis of 5-fluorouracil anti-tumor activity in colon cancer using a mechanistic pharmacokinetic/pharmacodynamic model
Source: PLoS Comput Biol. 2022 Nov 17;18(11):e1010685. doi: 10.1371/journal.pcbi.1010685 (PMC9671373; doi:10.1371/journal.pcbi.1010685)
Supplement: S2 Text — (PDF) [file pcbi.1010685.s002.pdf]

Sensitivity analysis

We adopted the method Link et al applied in the simulation study [1] to conduct the global sensitivity analysis. We used the Morris method to screen the parameters that have more influence on the model outputs. Then the variance-based global sensitivity analysis, often referred to as Sobol method, is conducted based on the selected parameters.

1 Morris method

A full global analysis of all model parameters was computationally expensive. Therefore, it is necessary to select subsets of parameters that seemed likely to have substantial effects on model outputs to reduce computational burden. We first applied the Morris method, which overcomes the obstacles of standard OAT approaches(one-at-the-time) that cannot measure the effect of the parameter interactions [2]. The full description of Morris method can be found in [1, 3]. For each parameter, the Morris mean indicating the overall effect of input factor on the output and Morris standard deviation indicating nonlinear effect in interactions with the other parameters are computed. Then, the selection of parameters that will be further evaluated in the Sobol analysis is done by ranking the parameters in order of importance to model output. If the L2-norm of Morris mean and standard deviation is larger than 0.5, the parameter is believed to have considerable effect on the model output and would be included in the Sobol analysis; otherwise, the parameters would not be included and their values would remain the estimated values. We conducted the Morris method three times over the tumor volume at 5th, 25th and 45th day after treatment begins and collected the parameters as the ones in the union of selected parameters identified by each attempt.

The Morris mean and standard deviation of each parameter with model outputs as the tumor volume sizes at 5th, 25th and 45th day post-treatment are shown in the bar plot Fig A-C. By visual examination, it can be observed that the changes in the parameters K_m, $K_{m,efflux}$, $K_{m,54}$, $K_{m,influx}$, P_{max}, $V_{max,efflux}$,$V_{max,54}$, $V_{max,75}$, $V_{max,influx}$, V_{max}, λ_d ,λ_g, k_{03}, k_{56} ,k_{59}, k_{95}, k_{65} created considerable perturbations on the model output and these were chosen to be analyzed using the Sobol analysis.

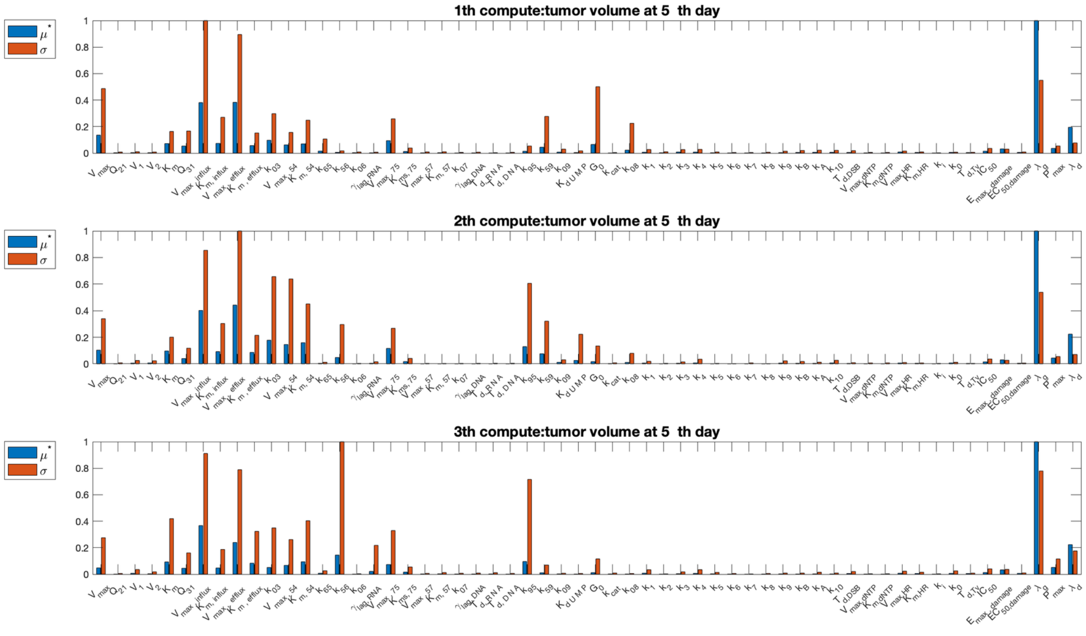

Fig A. Result of Morris method with tumor volume size at 5th day after treatment begins. The red bar represents the Morris mean; The blue bar represents the Morris standard deviation.

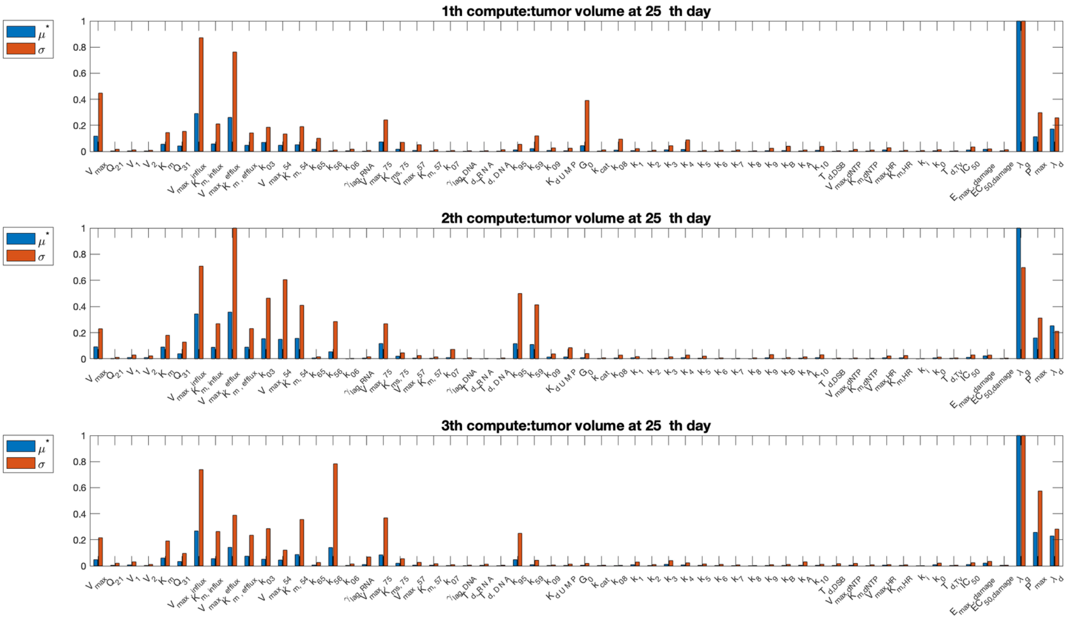

Fig B. Result of Morris method with tumor volume size at 25th day after treatment begins. The red bar represents the Morris mean; The blue bar represents the Morris standard deviation.

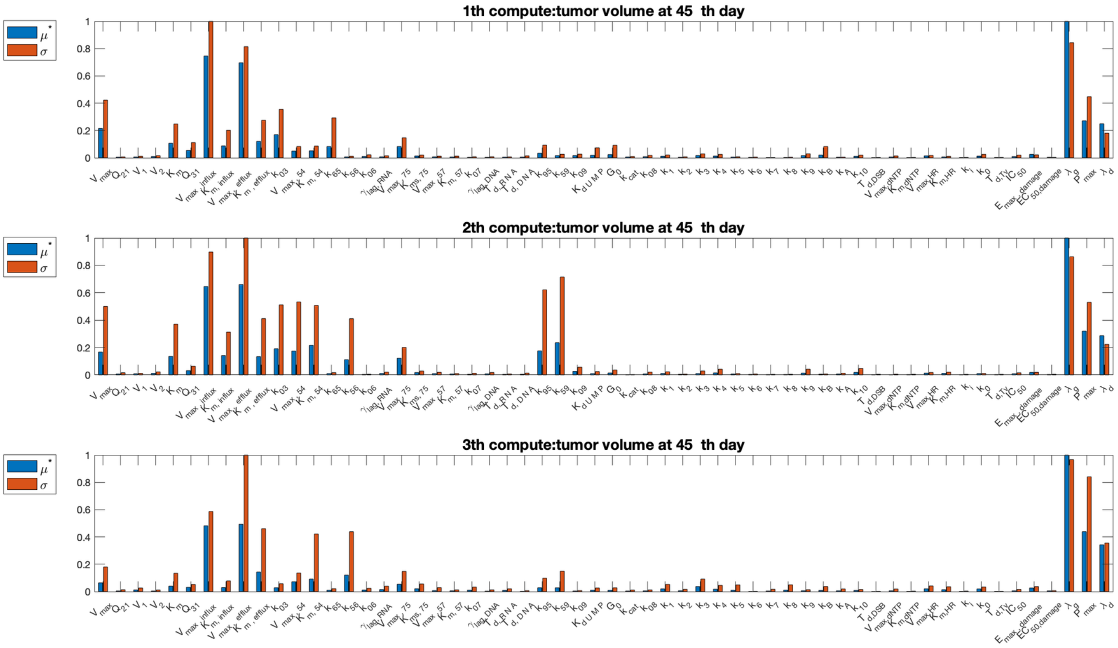

Fig C. Result of Morris method with tumor volume size at 45th day after treatment begins. The red bar represents the Morris mean; The blue bar represents the Morris standard deviation.

2 Sobol global analysis

We further used the Sobol global sensitivity to identify the degree of the changes in the model outputs produced by the joint interactions of the input parameters. The first-order and total order Sobol index are calculated for the parameters mentioned above. Specifically, the first-order Sobol index measures the fraction of total variance that is attributed to the variations in each input parameter. On the other hand, the total-order Sobol index provides the fraction of the overall response variance that can be attributed to any joint parameter variations that include variations of

concerned parameter. The approximations of the first-order and total-order Sobol index are detailed in [4].

The first order and total order Sobol index of each parameter with the tumor volume at 5th, 25th and 45 day after treatment begins are shown in Fig D. The figure presents that model outputs at 45 th day are more sensitive to λ_g, $P_{\max}$, $V_{max,efflux}$, and $V_{max,influx}$ than the other input factors, which is consistent with the result of preliminary screening by Morris method. It is noteworthy that the first-order and total-order Sobol index preserve that relative numerical relationships between the parameters reported by Morris method.

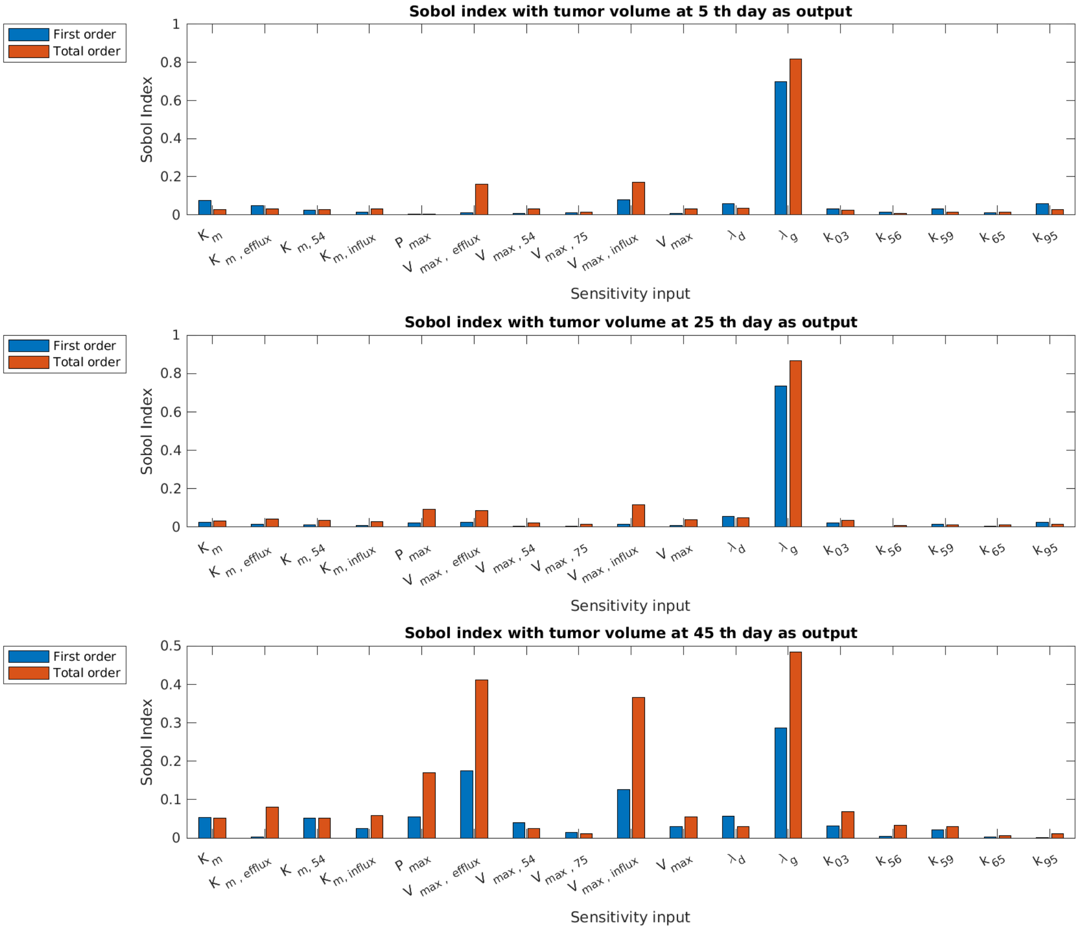

Fig D. The first order and total order Sobol index with the tumor volume at 5th, 25 th and 45th day posttreatment. The blue bar represents the first-order Sobol index; The orange bar represents the total-order Sobol index.

References

[1] Kathryn G. Link, Michael T. Stobb, Jorge Di Paola, Keith B. Neeves, Aaron L. Fogelson, Suzanne S. Sindi, and Karin Leiderman. A local and global sensitivity analysis of a mathematical model of coagulation and platelet deposition under flow. 13(7):e0200917.

[2] T. Sumner, E. Shephard, and I. D. L. Bogle. A methodology for global-sensitivity analysis of time-dependent outputs in systems biology modelling. 9(74):2156–2166.

[3] Francesca Campolongo, Jessica Cariboni, and Andrea Saltelli. An effective screening design for sensitivity analysis of large models. 22(10):1509–1518.

[4] Andrea Saltelli, Paola Annoni, Ivano Azzini, Francesca Campolongo, Marco Ratto, and Stefano Tarantola. Variance based sensitivity analysis of model output. Design and estimator for the total sensitivity index. 181(2):259–270.
