# Supplementary material for: Computational analysis of 5-fluorouracil anti-tumor activity in colon cancer using a mechanistic pharmacokinetic/pharmacodynamic model
Source: PLoS Comput Biol. 2022 Nov 17;18(11):e1010685. doi: 10.1371/journal.pcbi.1010685 (PMC9671373; doi:10.1371/journal.pcbi.1010685)
Supplement: S3 Text — (PDF) [file pcbi.1010685.s003.pdf]

Identifiability analysis

1 Parameter estimates and prior distributions

In order to gain knowledge of the order of the magnitude of the model parameters before defining their prior distributions, we employed the parameter optimization scheme by using `fmincon` solver coupled with `multistart` object in Matlab (ver. R2022a) to get the global optimal parameter values minimizing the least sum of squares(LSS). Then, the parameter search space for the MCMC algorithm is defined around these initial estimates in the form of prior distributions for the parameters, such that these initial estimates are used as the expected values for parameters with Gaussian prior distributions or the scale parameters for parameters with non-Gaussian prior distributions. The parameter estimates for the PK and cellular models, and the associated confidence intervals and selected prior distributions are presented in the Table A. The components of each parameter set $\boldsymbol{\Theta_i}, i = 1..3$ are also shown in the Table A. The prior distributions defined for the parameters in the nine TGI models are shown in Table B.

Table A. Parameter estimates generated by MCMC analysis and prior distribution choices for parameters defined for PK and cellular models

Parameter		Unit	Posterior mean($q_{0.25}$,$q_{0.75}$)	Prior
**PK model**				
	$V_{\max}$	μg·mL^{-1} ·min^{-1}	329.539(313.795,346.084)	U(0,1e+3)
	Q_{21}	mL·mg^{-1} ·min^{-1}	320.071(39.926,680.584)	U(0,1e+3)
$\boldsymbol{\Theta_1}$	V_1	mL· (mg tissue)$^{-1}$	16823.472(15776.621,18123.180)	U(0,5e+4)
	V_2	mL· (mg tissue)$^{-1}$	2999.821(2715.355,3266.188)	U(0,5e+3)
	K_m	μg· mL $^{-1}$	17334.977(16261.304,18220.139)	U(0,5e+4)
**Cellular model**				
	**5-FU in interstitial fluid and its anabolism**			
	Q_{31}	mL·mg^{-1} ·min^{-1}	0.981(0.915,1.033)	Weibull(1,2)
	$V_{\max,\text{influx}}$	pmol·mg^{-1} ·min^{-1}	538.451(537.306,539.436)	Normal(5.4e2, 50)
	$K_{\text{m,influx}}$	mL· (mg tissue)$^{-1}$	171.153(170.931,171.378)	Normal(1.71e2, 20)
	$V_{\max,\text{efflux}}$	pmol·mg^{-1} ·min^{-1}	536.642(536.044,537.006)	Normal(5.37e2, 50)
	$K_{\text{m, efflux}}$	pmol·mg^{-1}	6.704(6.571,6.829)	Normal(6.35,3)
	k_{03}	min^{-1}	135.995(134.929,137.845)	Normal(1.27e2, 20)
	$V_{\max,54}$	pmol·mg^{-1} ·min^{-1}	27.138(26.149,28.348)	Normal(2.52e1, 10)
	$K_{\text{m},54}$	pmol·mg^{-1}	2325.154(2324.075,2326.215)	Normal(2.32e3, 50)
	**5-FU incorporation into DNA and RNA**			
	k_{65}	min^{-1}	0.089(0.082,0.096)	U(1e-3, 0.8)
	k_{56}	min^{-1}	0.521(0.518,0.525)	U(1e-3, 1.1)
	k_{06}	min^{-1}	0.114(0.111,0.119)	U(1e-5, 0.8)
	$\gamma_{\text{lag, RNA}}$	a	0.310(0.299,0.326)	U(1e-5, 0.8)
	$V_{\max,75}$	pmol·mg^{-1} ·min^{-1}	0.0199(0.0171,0.0223)	Gamma(1,1)
	$K_{\text{m},75}$	pmol·mg^{-1}	0.8398(0.8344,0.8467)	Gamma(1,1)
	$V_{\max,57}$	pmol·mg^{-1} ·min^{-1}	5.534(5.525,5.542)	Weibull(5,2)
	$K_{\text{m},57}$	pmol· mg^{-1}	0.95908(0.95894,0.95922)	Weibull(1,2)
	k_{07}	min^{-1}	0.808(0.796,0.819)	Weibull(1,2)
$\boldsymbol{\Theta_2}$	$\gamma_{\text{lag, DNA}}$	a	0.554(0.549,0.560)	Weibull(1,2)
	$T_{d,RNA}$	day	0.396(0.363,0.428)	Weibull(1,2)
	$T_{d,DNA}$	day	4.360(4.344,4.378)	Weibull(5,2)
	σ^2_{intra}	b	199.9999(199.9888,200.0107)	Lognormal(4, 0.4)
	σ^2_{FNUC}	b	200.004(199.974,200.033)	Lognormal(4, 0.4)
	σ^2_{RNA}	b	4.991(4.958,5.025)	Lognormal(4,1)
	σ^2_{DNA}	b	2.009(1.996,2.022)	Lognormal(1,1)
	**TS inhibition**			

Table A. Parameter estimates generated by MCMC analysis and prior distribution choices for parameters defined for PK and cellular models

Parameter		Unit	Posterior mean($q_{0.25}$,$q_{0.75}$)	Prior
	k_{95}	min^{-1}	0.038(0.035,0.043)	Gamma(1,1)
	k_{59}	min^{-1}	2.882(2.877,2.888)	Weibull(5,2)
	k_{09}	min^{-1}	0.152(0.138,0.171)	Gamma(1,1)
	K_{dUMP}	a	21.935(21.932,21.938)	Weibull(17,2)
	G_0	$mg \cdot min^{-1}$	0.155(0.131,0.185)	Gamma(1,1)
	k_{cat}	min^{-1}	17.430(17.419,17.444)	Weibull(18,2)
	k_{08}	min^{-1}	0.032(0.021,0.044)	U(1e-3,0.8)
	α_{TS}	a	2.021e+0	fixed
	k_d	a	1.034e+0	fixed
	TS_0	$pmol \cdot mg^{-1}$	1.860e-2	fixed
	σ^2_{TS}	b	0.203(0.199,0.207)	Lognormal(1,1)
	σ^2_{dUMP}	b	2(1.9999,2.0001)	Lognormal(1,0.5)
	**dNTP pool imbalance**			
	k_1	a	0.213(0.211,0.215)	U(1e-3,1)
	k_2	a	32.50(32.49,32.51)	Normal(32,10)
	k_3	a	3.713(3.711,3.714)	Weibull(4.2)
	k_4	a	73.389(73.368,73.409)	Weibull(73,2)
	k_5	a	3.695(3.682,3.705)	Weibull(4,2)
	k_6	a	0.303(0.302,0.305)	U(1e-3,1)
	k_7	a	3.339(3.334,3.345)	Weibull(4, 2)
	k_8	a	1.382(1.375,1.387)	Weibull(10,1)
	k_9	a	0.27672(0.27663,0.27684)	U(1e-3,1)
	k_B	a	35.65(35.63,35.67)	Normal(35,10)
	k_A	a	6.038(6.022,6.054)	Weibull(7,2)
	k_{10}	a	0.0794(0.0789,0.08)	U(1e-4,0.4)
$\boldsymbol{\Theta_3}$	γ_{dNTP}	a	1.500e-1	fixed
	σ^2_{dNTP}	b	0.013(0.0068,0.0158)	Gamma(1,1)
	**DSB induction**			
	$T_{d,DSB}$	Day	1.801(1.800,1.802)	Weibull(1.8, 2)
	$V_{\max,dNTP}$	min^{-1}	10.516(10.515,10.519)	Weibull(10, 2)
	$K_{m,dNTP}$	a	151.918(151.907,151.929)	Normal(152, 50)
	$V_{\max,HR}$	Thousands count/min	10.082(10.072,10.087)	Weibull(10, 2)
	$K_{m,HR}$	Thoudands count	194.020(194.014,194.028)	Normal(194, 50)
	k_i	min^{-1}	0.121(0.112,0.129)	U(1e-4, 1)
	k_0	min^{-1}	2.803(2.789,2.815)	Weibull(3,2)
	γ_{DSB}	a	6.000e-1	fixed
	σ^2_{DSB}	b	199.9999(199.9995,200.0003)	Lognormal(4, 0.4)

a: unitless parameter

b: variance of the measurement noise.

The numbers of samples used for calculating the credible intervals of the parameters in sets $\boldsymbol{\Theta_i}, i = 1..3$ are 12000, 9000, and 5748 respectively.

2 Approximated posterior distributions of the model parameters

We used the approximated posterior distributions for each parameter to evaluate the model identifiability. The approximated marginal posterior distributions for the parameters from Table A and Table B are presented by the histograms given in Fig A - L . We classified the parameters into identifiable and unidentifiable groups according to the compactness of the shape and support of their distributions. The histograms shown in Fig A - L for most of the parameters have compact bell-like shape, indicating that there exists a well-defined maximum for each of these parameters

Table B. Parameter estimates and prior choices for parameters defined for the nine TGI models.

Model		Model 1	Model 2	Model 3	Model 4	Model 5	Model 6	Model 7	Model 8	Model 9
$T_{\mathrm{d,Tv}}$,	Estimate	4.9738e2	5.8137e2	8.9345e2	8.9345e2	1.2537e3	8.9345e2	8.9345e2	1.1160e3	1.3002e3
min	Prior	Norm(500,100)	Norm(500,100)	Norm(900,100)	b	Norm(800,100)	b	b	Norm(800,100)	Norm(900,100)
IC_{50},	Estimate	7.6262e0	1.4192e0	3.7658e-3	3.7658e-3	6.9275e-1	3.7658e-3	3.7658e-3	8.7883e1	7.2201e1
pmol $\cdot$ mg^{-1}	Prior	Weibull(10,2)	Unif(1e-8,5)	Unif(1e-8,2)	b	Unif(1e-8,2)	b	b	Norm(50, 100)	Norm(50, 100)
$E_{\mathrm{max},damage}$,	Estimate	6.7387e-1	2.3961e1	1.7511e-1	1.7511e-1	7.8173e-1	1.7511e-1	1.7511e-1	5.0672e-1	1.5250e0
units	Prior	Unif(1e-8,1)	Weibull(22,2)	Unif(1e-8,2)	b	Unif(1e-8,2)	b	b	Unif(1e-8,1)	Unif(1e-8,5)
$EC_{50,damage}$,	Estimate	5.1420	161.4349	0.2380	0.2380	0.5761	0.2380	0.2380	62.8754	49.6560
units	Prior	Weibull(5.2,2)	Weibull(157,2)	Unif(1e-8,2)	b	Unif(1e-8,2)	b	b	Unif(1e-8,100)	Unif(1e-8,100)
λ_g ,	Estimate	7.6643e-5	4.2827e-4	2.1252e-4	2.5101e-4	2.2528e-4	2.8371e-4	1.5008e-4	2.3163e-4	2.7370e-4
min^{-1}	Prior	Unif(1e-8,1)	Unif(1e-8,1)	Unif(1e-8,1)	Unif(1e-8,1)	Unif(1e-8,1)	Unif(1e-8,1)	Unif(1e-8,1)	Unif(1e-8,1)	Unif(1e-8,1)
P_{max} ,	Estimate	4.8414e0	1.4204e0	1.2468e0	1.7230e0	2.3627e0	11.623e1	2.6419e0	8.9848e0	7.5644e0
cm^{-3}	Prior	Weibull(5,2)	Unif(1,10)	Unif(1,2)	Unif(1,5	Unif(0.5,4)	Weibull(13,2)	Weibull(13,2)	Weibull(5,2)	Weibull(5,2)
λ_{d},	Estimate	1.1564e-5	2.7973e-5	5.0506e-5	4.4318e-5	3.1695e-6	3.4289e-5	1.7707e-5	4.0360e-6	5.5131e-6
min^{-1}	Prior	Unif(1e-8,1)	Unif(1e-8,1)	Unif(1e-8,1)	Unif(1e-8,1)	Unif(1e-8,1)	Unif(1e-8,1)	Unif(1e-8,1)	Unif(1e-8,1)	Unif(1e-8,1)
$\sigma^2_{treated}$	Esitmate	2.2878e-2	2.0999e-3	1.8068e0	5.2956e-1	6.2889e-4	1.0461e-2	7.2275e-1	1.0464e-2	1.3928e-2
	Prior	Gamma(1,1)	Gamma(1,1)	Gamma(1,1)	Gamma(1,1)	Gamma(1,1)	Gamma(1,1)	Gamma(1,1)	Gamma(1,1)	Gamma(1,1)
$\sigma^2_{control}$	Estimate	3.5396e-2	1.6150e-1	5.3202e-3	1.4767e-1	2.4629e-1	1.5163e0	1.0279e-1	5.0422e-2	8.6216e-1
	Prior	Gamma(1,1)	Gamma(1,1)	Gamma(1,1)	Gamma(1,1)	Gamma(1,1)	Gamma(1,1)	Gamma(1,1)	Gamma(1,1)	Gamma(1,1)
γ_{Tv}	Estimate	a	a	a	a	a	a	a	a	a

a Fixed as 0.2.

b: No prior distribution beacause the parameter value is the same as TGI model (3) counterpart.

and that those parameters are identifiable. The list of unidentifiable parameters are given in the figure captions.

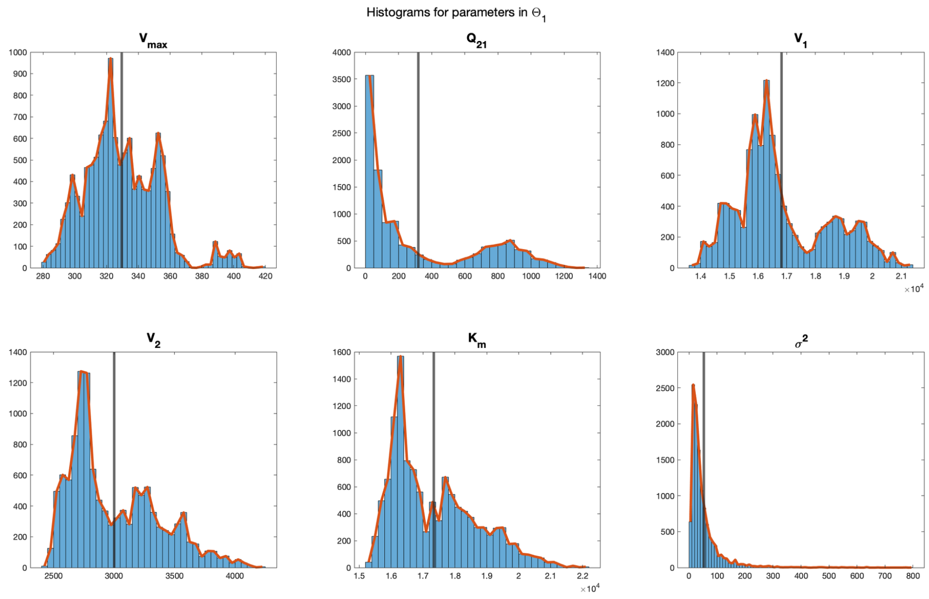

Fig A. Histograms of the parameters defined in the PK model. The grey vertical line represents the mean parameter value reported in Table A.

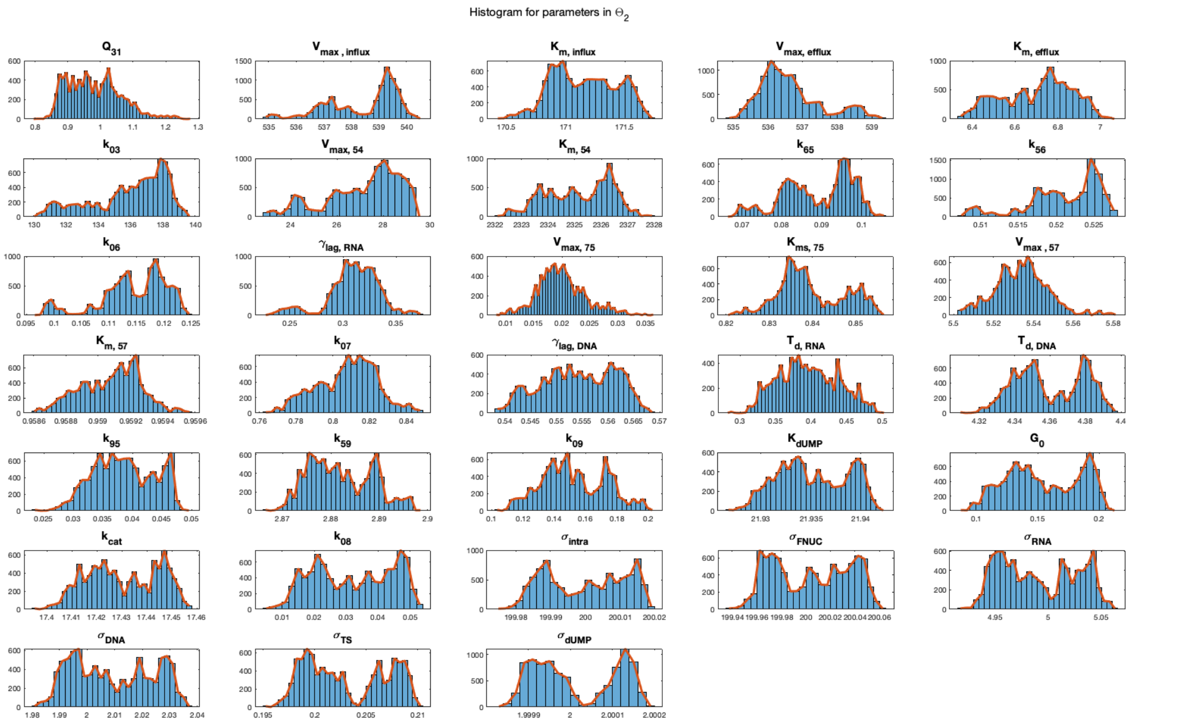

Fig B. Histograms of the parameters defined for model variables A_3(5-FU in interstitial fluid) to A_9(dUMP) in the cellular model. The grey vertical line represents the mean parameter value reported in Table A. The parameters apart from Q_{31}, $K_{m,influx}$, k_{65}, $\gamma_{lag,DNA}$, $T_{d,DNA}$, k_{95}, k_{59}, G_0, and k_{cat} are identifiable. The variances of the measurement noises are all unidentifiable.

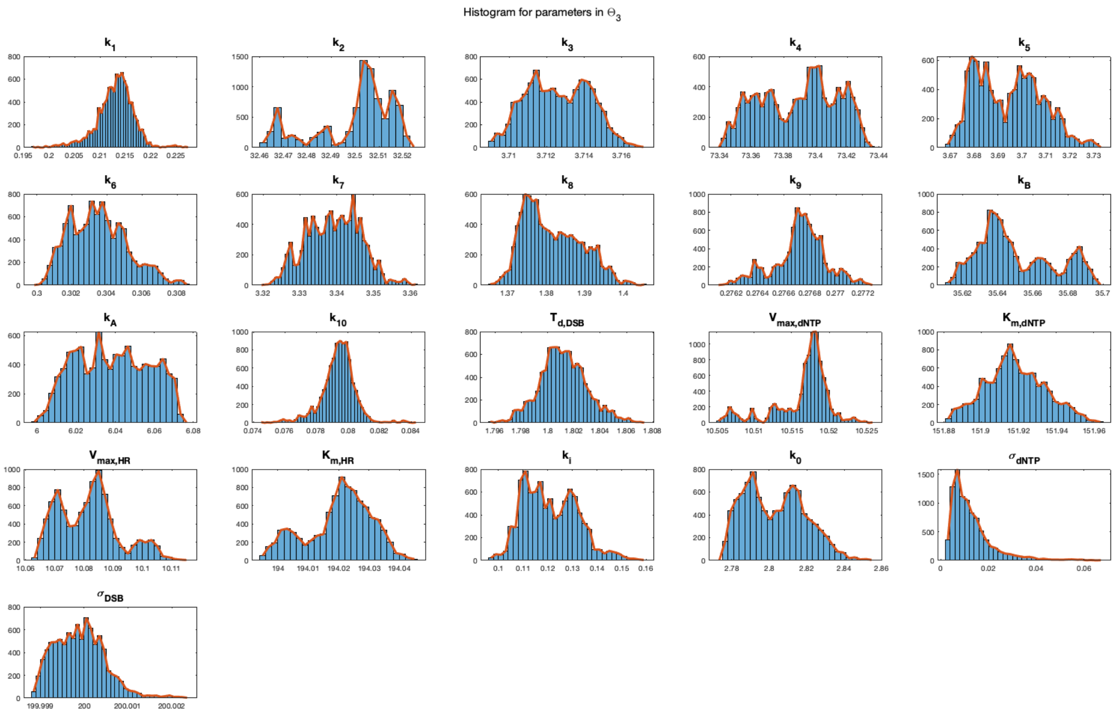

Fig C. Histograms of the parameters defined for model variables N_{dNTP} and N_{DSB} in the cellular model. The grey vertical line represents the mean parameter value reported in Table A. The parameters apart from k_3, k_4, k_5, k_B, k_A, k_i, and k_0 are identifiable.

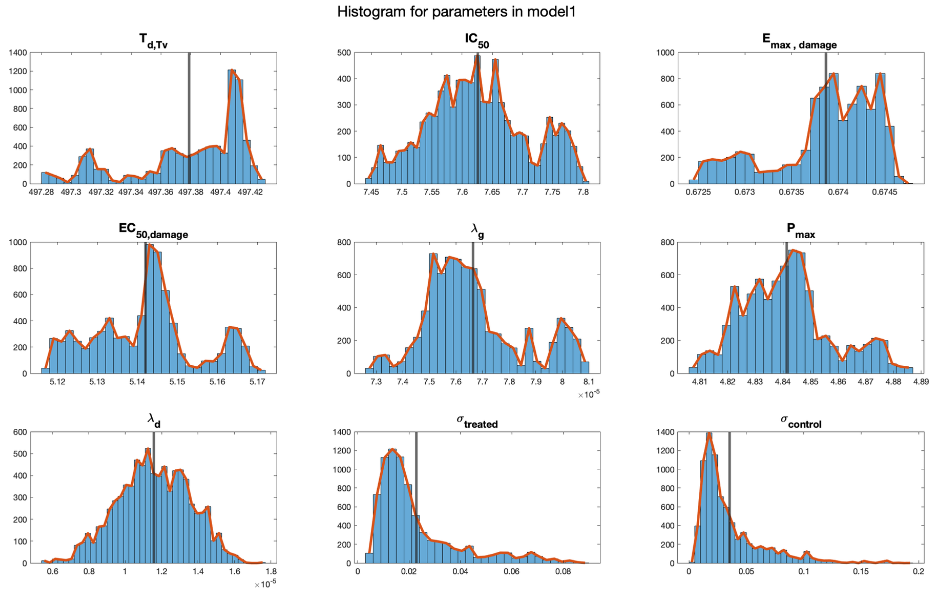

Fig D. Histograms of the parameters defined for TGI model 1. The grey vertical line represents the mean parameter value reported in Table B. As shown in the estimated posterior distributions of TGI model parameters, all the parameters apart from $E_{\max,damage}$ are identifiable.

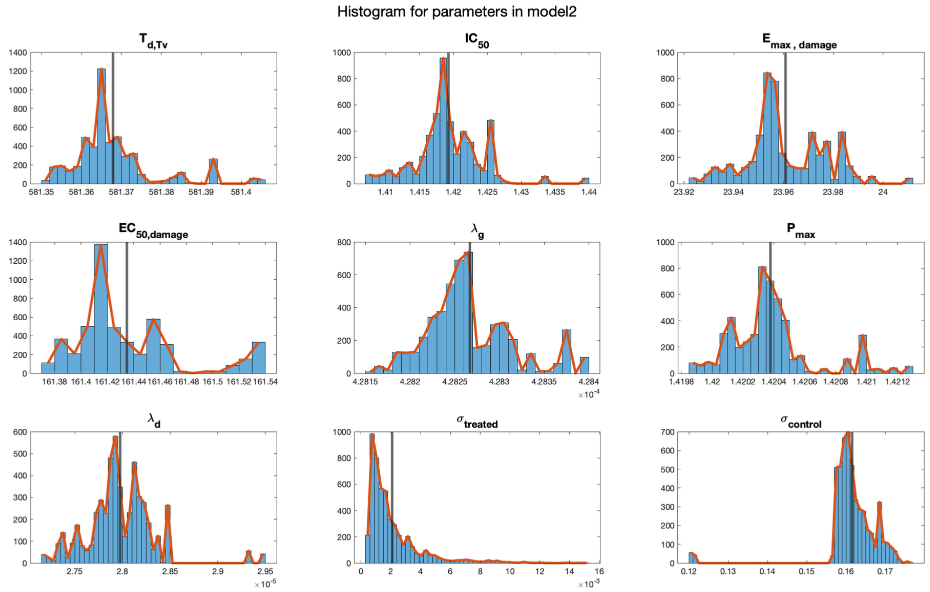

Fig E. Histograms of the parameters defined for TGI model 2. The grey vertical line represents the mean parameter value reported in Table B. In this case, the estimated distributions of all the parameters are monomodal.

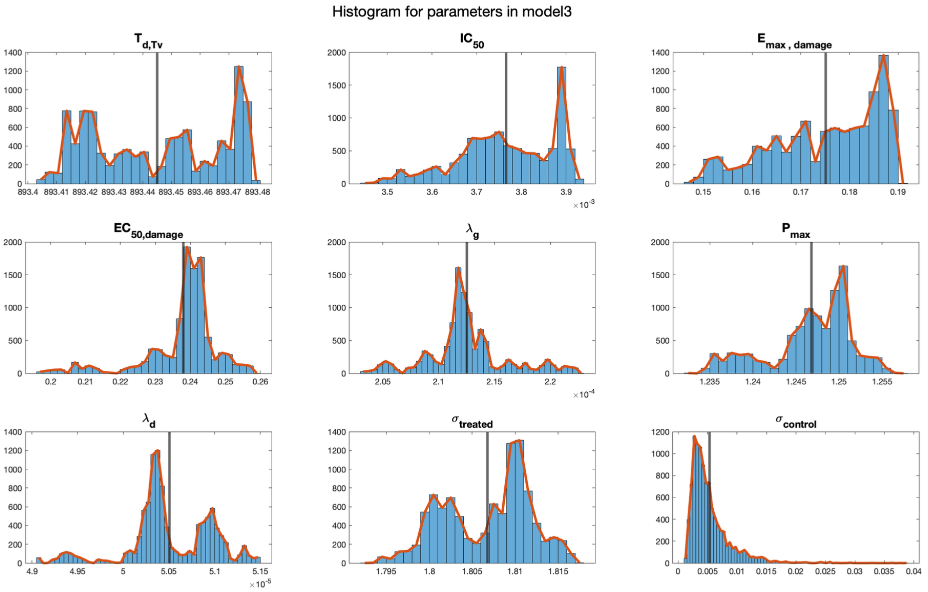

Fig F. Histograms of the parameters defined for TGI model 3. The grey vertical line represents the mean parameter value reported in Table B. It can be seen that the estimated distributions of almost all the parameters apart from $T_{d,Tv}$, $\sigma^2_{treated}$ λ_d, and $P_{\max}$ are monomodal.

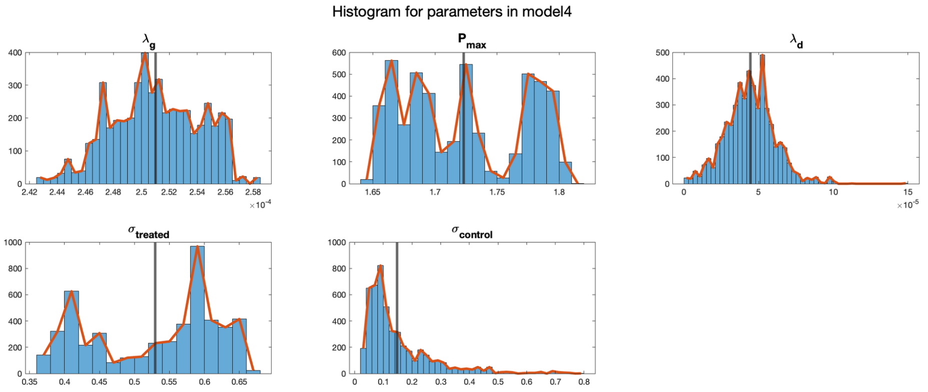

Fig G. Histograms of the parameters defined for TGI model 4. The grey vertical line represents the mean parameter value reported in Table B. The figure shows that λ_g, $P_{\max}$ and $\sigma^2_{treated}$ are not identifiable.

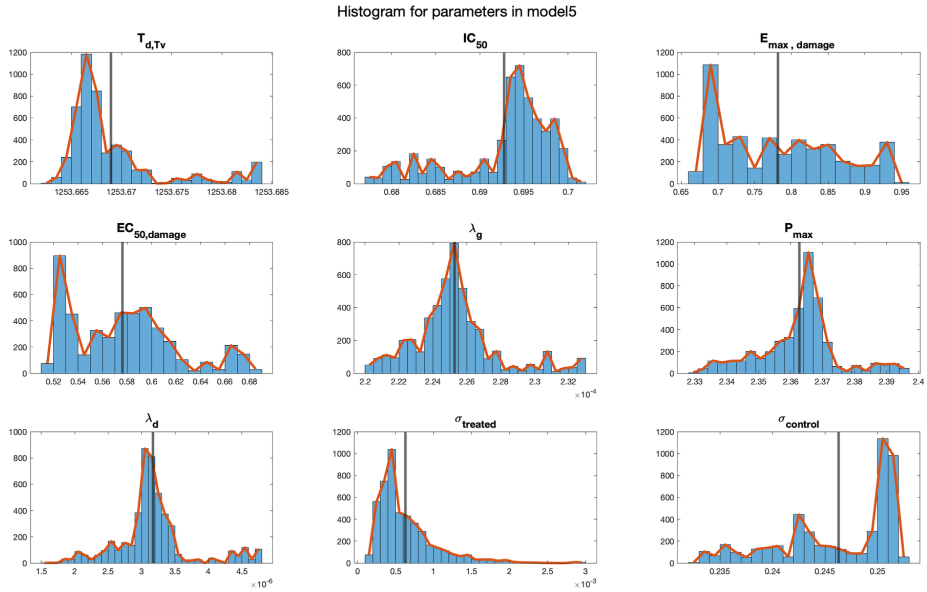

Fig H. Histograms of the parameters defined for TGI model 5. The grey vertical line represents the mean parameter value reported in Table B. In this case, the estimated distribution of all the parameters apart from $EC_{50,damage}$ are identifiable.

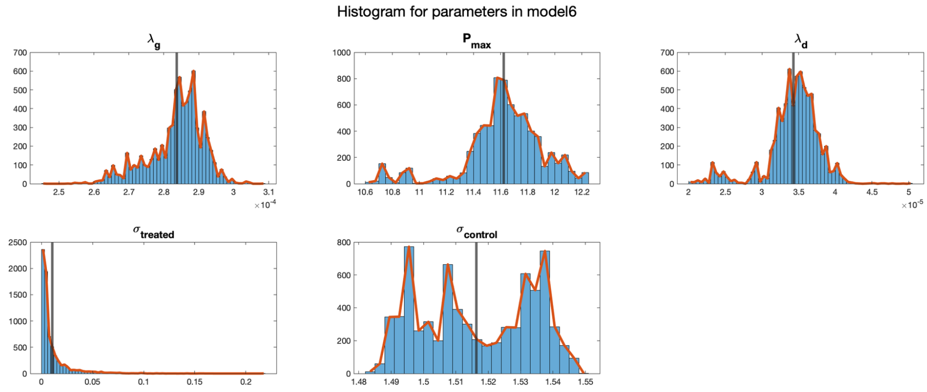

Fig I. Histograms of the parameters defined for TGI model 6. The grey vertical line represents the mean parameter value reported in Table B. In this case, only $\sigma^2_{control}$ is not identifiable.

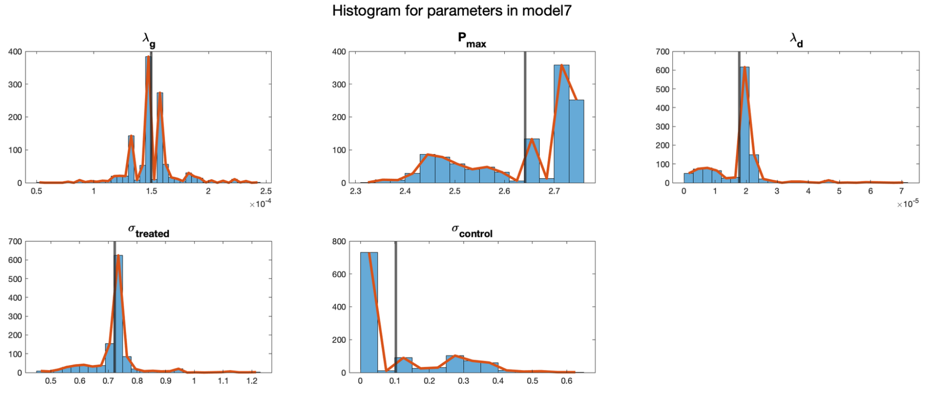

Fig J. Histograms of the parameters defined for TGI model 7. The grey vertical line represents the mean parameter value reported in Table B.In this case, all the TGI parameters to be estimated are identifiable.

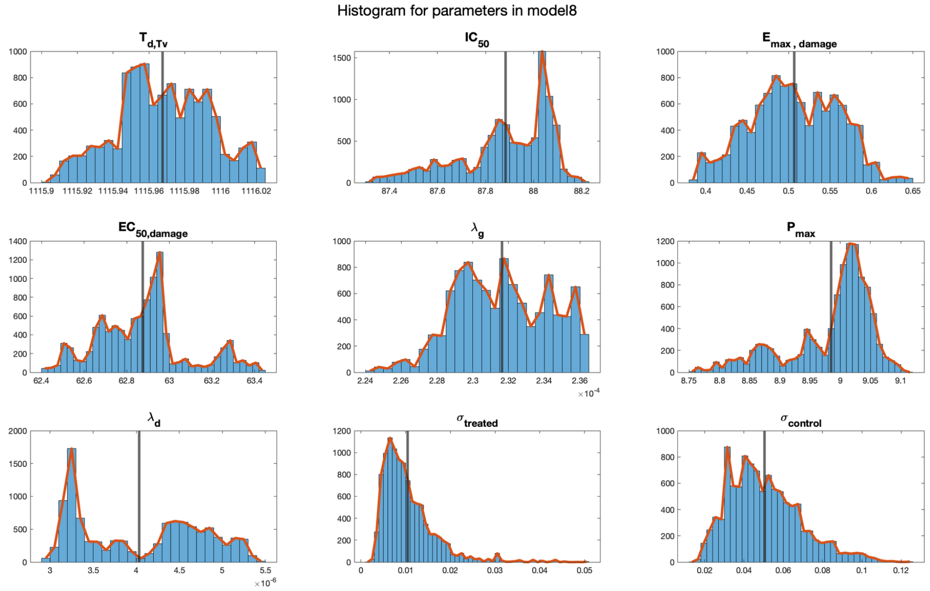

Fig K. Histograms of the parameters defined for TGI model 8. The grey vertical line represents the mean parameter value reported in Table B. The figure shows that the estimated distributions of $T_{d,Tv}$, $E_{\max,damage}$, λ_g, and $\sigma^2_{control}$ are not identifiable.

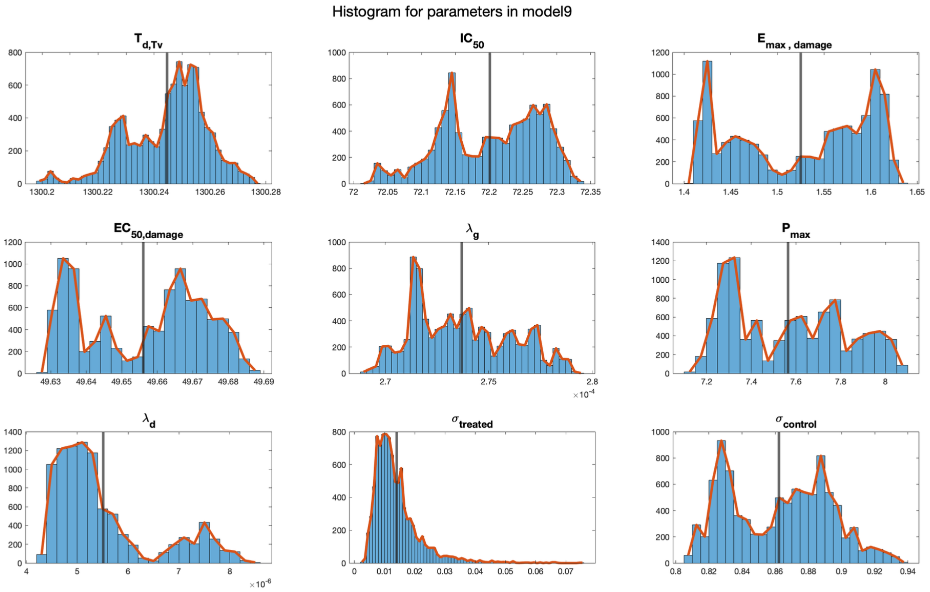

Fig L. Histograms of the parameters defined for TGI model 9. The grey vertical line represents the mean parameter value reported in Table B. The figure shows that the estimated distributions of $T_{d,Tv}$, IC_{50}, $E_{\max,damage}$, $EC_{50,damage}$ and $\sigma^2_{control}$ are not identifiable.

3 Model calibration with uniform prior distributions

We also employed the MCMC technique with uniform prior distributions. The estimated values for the the components of the parameter set $\boldsymbol{\Theta_i}, i = 2, 3$, and the associated confidence intervals and ranges for the uniform prior distributions are presented in the Table C. Likewise, the uniform prior distributions defined for the parameters in the nine TGI models and the parameter estimates are shown in Table D. The comparisons of the estimated values of the parameters shown in Table A and Table C, as well as in Table B and Table D show that the estimation results are similar for both estimation approaches even though different prior distributions were used. Therefore, we based our computational analysis on the model with the results of MCMC approach shown in Table A and Table B. The approximated marginal posterior distributions for the parameters in Table C are presented by the histograms given in Fig N and Fig O. Fig Q-Fig Y display the approximated marginal posterior distributions for the parameters estimated for the nine TGI models. The time profiles of the model variables in cellular model generated by simulating the model with parameter estimates shown in Table C are shown in Fig M, and the time profiles of the outputs of the nine TGI models with parameter estimates in Table D are shown in Fig P.

Table C. Estimates generated by MCMC method of the parameters defined for cellular model and the corresponding uniform prior distributions

Parameter	Unit	Posterior mean $(q_{0.25}, q_{0.75})$	Lower bound	Upper bound
**Cellular model**				
**5-FU in interstitial fluid and its anabolism**				
Q_{31}	$\mathrm{mL \cdot mg^{-1} \cdot min^{-1}}$	0.843(0.729,0.943)	0.1	10
$V_{\text{max, influx}}$	$\mathrm{pmol \cdot mg^{-1} \cdot min^{-1}}$	537.821(537.468,538.1)	100	1000
$K_{\text{m, influx}}$	$\mathrm{mL \cdot \left(mg^{-1}\ tisue\ \right)^{-1}}$	180.865(177.858,183.427)	40	400
$V_{\text{max, efflux}}$	$\mathrm{pmol \cdot mg^{-1} \cdot min^{-1}}$	530.619(525.4,535.375)	100	1000
$K_{\text{m, efflux}}$	$\mathrm{pmol \cdot mg^{-1}}$	6.929(6.819,7.047)	0.1	20
k_{03}	$\mathrm{min^{-1}}$	125.241(124.516,126.057)	25	250
$V_{\text{max},54}$	$\mathrm{pmol \cdot mg^{-1} \cdot min^{-1}}$	24.436(23.394,25.465)	0.1	100
$K_{\text{m},54}$	$\mathrm{pmol \cdot mg^{-1}}$	2323.34(2322.663,2323.939)	500	5000
**5-FU incorporation into DNA and RNA**				
k_{65}	$\mathrm{min^{-1}}$	0.104(0.097,0.110)	1.00e-03	0.8
k_{56}	$\mathrm{min^{-1}}$	0.499(0.493,0.505)	1.00e-03	1.1
k_{06}	$\mathrm{min^{-1}}$	0.279(0.256,0.303)	1.00e-05	0.8
$\gamma_{lag,\ \text{RNA}}$	a	0.145(0.099,0.193)	1.00e-05	0.8
$V_{\text{max},75}$	$\mathrm{pmol \cdot mg^{-1} \cdot min^{-1}}$	0.021(0.0167,0.025)	1.00e-03	10
$K_{\text{m},75}$	$\mathrm{pmol \cdot mg^{-1}}$	0.826(0.758,0.892)	1.00e-03	10
$V_{\text{max},57}$	$\mathrm{pmol \cdot mg^{-1} \cdot min^{-1}}$	5.824(5.782,5.866)	1.00e-01	20
$K_{\text{m},57}$	$\mathrm{pmol \cdot mg^{-1}}$	0.814(0.677,0.930)	0.05	5
k_{07}	$\mathrm{min^{-1}}$	0.909(0.892,0.929)	0.05	5
$\boldsymbol{\Theta_2}$ $\gamma_{\text{lag, DNA}}$	a	0.561(0.552,0.568)	0.1	20
$T_{d,RNA}$	day	0.688(0.677,0.698)	1.00e-03	10
$T_{d,DNA}$	day	4.453(4.442,4.464)	0.1	20
σ^2_{intra}	b	200.030(200.023,200.033)	5	500
σ^2_{FNUC}	b	200.039(200.029,200.045)	5	500
σ^2_{RNA}	b	5.063(5.042,5.076)	1.00e-03	20
σ^2_{DNA}	b	2.020(2.015,2.024)	2.01e+00	10
**TS inhibition**				
k_{95}	$\mathrm{min^{-1}}$	0.034(0.033,0.036)	1.00e-03	10
k_{59}	$\mathrm{min^{-1}}$	2.864(2.857,2.871)	0.1	20
k_{09}	$\mathrm{min^{-1}}$	0.149(0.116,0.173)	1.00e-03	10
K_{dUMP}	a	21.975(21.955,21.300)	0.1	100
G_0	$\mathrm{mg \cdot min^{-1}}$	0.499(0.456,0.536)	1.00e-03	10
k_{cat}	$\mathrm{min^{-1}}$	17.435(17.426,17.444)	0.1	100
k_{08}	$\mathrm{min^{-1}}$	0.105(0.081,0.117)	1.00e-03	0.8
α_{TS}	a	2.021	c	c
k_d	a	1.034	c	c
TS_0	pmol.mg $\mathrm{mg^{-1}}$	1.86e-02	c	c
σ^2_{TS}	b	0.297(0.263,0.325)	2.03e-01	10
σ^2_{dUMP}	b	2.009(2.004,2.013)	2	20
**dNTP pool imbalance**				
k_1	a	0.216(0.214,0.218)	1.00e-03	1

Table C. Estimates generated by MCMC method of the parameters defined for cellular model and the corresponding uniform prior distributions

Parameter		Unit	Posterior mean $(q_{0.25}, q_{0.75})$	Lower bound	Upper bound
	k_2	a	32.495(32.488,32.5)	0.1	100
	k_3	a	3.622(3.591,3.632)	0.01	10
	k_4	a	73.339(73.331,73.343)	0.1	200
	k_5	a	3.758(3.757,3.759)	0.01	10
	k_7	a	0.228(0.218,0.240)	1.00e-03	1
	k_7	a	3.273(3.268,3.278)	0.01	10
	k_9	a	1.340(1.338,1.341)	0.01	50
	k_B	a	0.274(0.271,0.277)	1.00e-03	1
	k_A	a	35.666(35.661,35.67)	1	100
$\mathbf{\Theta_3}$	k_{10}	a	6.054(6.023,6.088)	0.1	20
	γ_{dNTP}	a	0.079(0.078,0.078)	1.00e-04	0.4
	σ^2_{dNTP}	b	0.012(0.006,0.015)	0.001	10
	**DSB induction**				
	$T_{d,DSB}$	Day	1.816(1.793,1.832)	0.1	5
	$V_{\max,dNTP}$	min^{-1}	10.529(10.523,10.534)	0.1	50
	$K_{m,dNTP}$	a	151.903(151.9,151.905)	1	500
	$V_{\max,HR}$	Thousands count/min	10.069(10.065,10.074)	0.1	50
	$K_{m,HR}$	Thousands count	193.999(193.9986,194.0003)	1	500
	k_i	min^{-1}	0.1235(0.1227,0.1242)	1.00e-04	1
	k_0	min^{-1}	2.7932(2.793,2.7932)	0.1	10
	γ_{DSB}	a	0.6	c	c
	σ^2_{DSB}	b	199.9677(199.96,199.9717)	5	200

a: unitless parameter
b: variance of the measurement noise
c: no uniform distribution assigned

The numbers of samples used for calculating the credible intervals of the parameters in sets $\mathbf{\Theta_i}, i = 2, 3$ are 25000 and 13500 respectively.

Table D. Estimates generated by MCMC method of the parameters defined for nine TGI models and the corresponding uniform prior distributions

Parameter		Unit	Posterior mean $(q_{0.25}, q_{0.75})$	Lower bound	Upper bound
	$T_{d,Tv}$	min	496.057(494.885,496.791)	100	1000
	IC_{50}	$\text{pmol} \cdot mg^{-1}$	7.445(7.308,7.613)	0.1	50
	$E_{max,damage}$	units	0.942(0.913,0.979)	1.00e-08	1
	$EC_{50,damage}$	units	5.422(5.4,5.45)	1.00e-08	20
**TGI model 1**	λ_g	min^{-1}	7.686e-05(7.679e-05,7.696e-05)	1.00e-08	1
	P_{max}	cm^{-3}	5.126(5.108,5.149)	0.1	20
	λ_d	min^{-1}	1.254e-05(1.141e-05,1.373e-05)	1.00e-08	1
	$\sigma^2_{treated}$	a	0.041(0.023,0.053)	0.001	10
	$\sigma^2_{control}$	a	0.035(0.017,0.039)	0.001	10
	$T_{d,Tv}$	min	598.905(598.479,599.342)	100	10000
	IC_{50}	$\text{pmol} \cdot mg^{-1}$	3.929(3.8311,4.06)	1.00e-08	5
	$E_{max,damage}$	units	24.056(24.054,24.059)	0.1	50
	$EC_{50,damage}$	units	164.071(163.993,164.155)	1	400
**TGI model 2**	λ_g	min^{-1}	0.00044395(0.00044289,0.00044515)	1.00e-08	1
	P_{max}	cm^{-3}	1.312(1.143,1.464)	1	10
	λ_d	min^{-1}	2.711e-05(2.656e-05,2.756e-05)	1.00e-08	10
	$\sigma^2_{treated}$	a	0.0069717(0.0028756,0.0069245)	0.001	10
	$\sigma^2_{control}$	a	0.187(0.181,0.194)	0.001	10
	$T_{d,Tv}$	min	894.4855(894.174,894.7097)	150	1500
	IC_{50}	$\text{pmol} \cdot mg^{-1}$	0.0048747(0.0047874,0.0049994)	1.00e-08	2
	$E_{max,damage}$	units	0.19982(0.19774,0.20324)	1.00e-08	2
**TGI model 3**	$EC_{50,damage}$	units	0.23311(0.22492,0.23664)	1.00e-08	2
	λ_g	min^{-1}	0.00022933(0.00019991,0.00025606)	1.00e-08	1
	P_{max}	cm^{-3}	1.2246(1.223,1.2258)	1	2
	λ_d	min^{-1}	6.549e-05(4.922e-05,8.143e-05)	1.00e-08	1

Table D. Estimates generated by MCMC method of the parameters defined for nine TGI models and the corresponding uniform prior distributions

Parameter		Unit	Posterior mean $(q_{0.25}, q_{0.75})$	Lower bound	Upper bound
	$\sigma^2_{treated}$	a	0.0083436(0.0011709,0.0038854)	0.001	10
	$\sigma^2_{control}$	a	0.0070767(0.0040296,0.0095661)	0.001	10
	λ_g	min^{-1}	0.000202(0.000183,0.000218)	1.00e-08	1
	P_{max}	cm^{-3}	1.725(1.724,1.726)	1	5
**TGI model 4**	λ_d	min^{-1}	1.012e-05(2.511e-06,1.662e-05)	1.00e-08	1
	$\sigma^2_{treated}$	a	0.00746(0.00366,0.00952)	0.001	10
	$\sigma^2_{control}$	a	0.253(0.224,0.254)	0.001	10
	$T_{d,Tv}$	min	1264.483(1264.139,1264.686)	150	1500
	IC_{50}	$\mathrm{pmol} \cdot mg^{-1}$	0.645(0.635,0.645)	1.00e-08	2
	$E_{max,damage}$	units	0.823(0.813,0.833)	1.00e-08	2
	$EC_{50,damage}$	units	0.465(0.460,0.470)	1.00e-08	2
**TGI model 5**	λ_g	min^{-1}	0.000224(0.000217,0.000228)	1.00e-08	1
	P_{max}	cm^{-3}	2.3155(2.3135,2.3154)	0.5	4
	λ_d	min^{-1}	7.68e-06(2.34e-06,1.01e-05)	1.00e-08	1
	$\sigma^2_{treated}$	a	0.00099(0.00042,0.0011)	0.001	10
	$\sigma^2_{control}$	a	0.00158(0.00114,0.00188)	0.001	10
	λ_g	min^{-1}	0.000261(0.000244,0.000278)	1.00e-08	1
	P_{max}	cm^{-3}	11.622(11.621,11.623)	1.00e-02	50
**TGI model 6**	λ_d	min^{-1}	3.219e-05(1.649e-05,4.569e-05)	1.00e-08	1
	$\sigma^2_{treated}$	a	0.138(0.053,0.203)	0.001	10
	$\sigma^2_{control}$	a	0.234(0.163,0.284)	0.001	10
	λ_g	min^{-1}	0.000149(0.000125,0.000170)	1.00e-08	1
	P_{max}	cm^{-3}	2.386(2.36,2.413)	0.5	4
**TGI model 7**	λ_d	min^{-1}	2.396e-05(9.173e-06,3.223e-05)	1.00e-08	1
	$\sigma^2_{treated}$	a	0.00236(0.00115,0.00222)	0.001	10
	$\sigma^2_{control}$	a	0.065(0.052,0.073)	0.001	10
	$T_{d,Tv}$	min	1111.1(1109.1,1112.9)	150	1500
	IC_{50}	$\mathrm{pmol} \cdot mg^{-1}$	87.6(87.2,88.1)	5.00e-01	500
	$E_{max,damage}$	units	0.664(0.506,0.872)	1.00e-08	1
	$EC_{50,damage}$	units	62.541(62.346,62.763)	1.00e-08	100
**TGI model 8**	λ_g	min^{-1}	0.000231(0.00023052,0.00023134)	1.00e-08	1
	P_{max}	cm^{-3}	9.054(9.024,9.078)	1.00e-02	20
	λ_d	min^{-1}	1.966e-05(1.018e-05,2.891e-05)	1.00e-08	1
	$\sigma^2_{treated}$	a	0.056(0.013,0.090)	0.001	10
	$\sigma^2_{control}$	a	0.172(0.069,0.248)	0.001	10
	$T_{d,Tv}$	min	1299.783(1299.643,1299.942)	150	1500
	IC_{50}	$\mathrm{pmol} \cdot mg^{-1}$	72.089(72.077,72.097)	5.00e-01	500
	$E_{max,damage}$	units	1.503(1.499,1.507)	1.00e-08	5
	$EC_{50,damage}$	units	49.648(49.647,49.650)	1.00e-08	100
**TGI model 9**	λ_g	min^{-1}	0.000257(0.000250,0.000263)	1.00e-08	1
	P_{max}	cm^{-3}	7.5646(7.5646,7.5646)	1.00e-02	20
	λ_d	min^{-1}	8.491e-06(3.263e-06,1.312e-05)	1.00e-08	1
	$\sigma^2_{treated}$	a	0.104(0.097,0.106)	0.001	10
	$\sigma^2_{control}$	a	0.020877(0.0063842,0.015029)	0.001	10

a: variance of measurement noise

The numbers of samples used for calculating the credible intervals of the parameters in nine TGI models are 6001, 17501, 10602, 13951, 8001, 10001, 12751, 10001, and 8501 respectively.

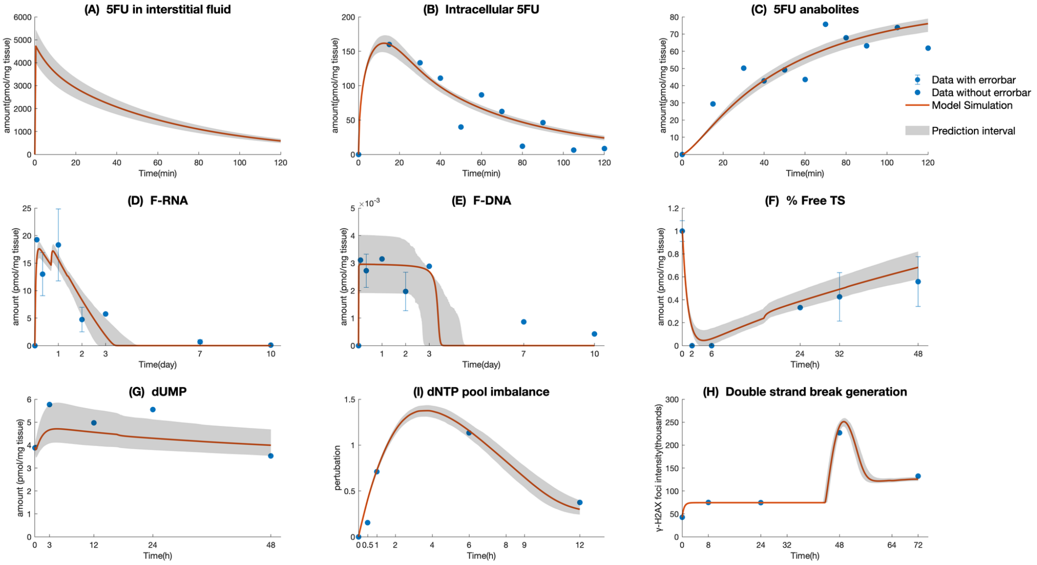

Fig M. Time profiles of intermediate components in cellular model generated by simulating the model with parameter estimates shown in Table C. (A) 5-FU in interstitial fluid, (B) intra-cellular 5-FU, (C) 5-FU anabolites, (D) F-RNA, (E) F-DNA, (F) % Free TS, (G) dUMP, (H) dNTP pool imbalance, (I) double-strand break generation. Red curve, model simulation; solid blue circle, literature data points; and error bar represents standard errors. The shaded areas represent the 0.7 credible regions.

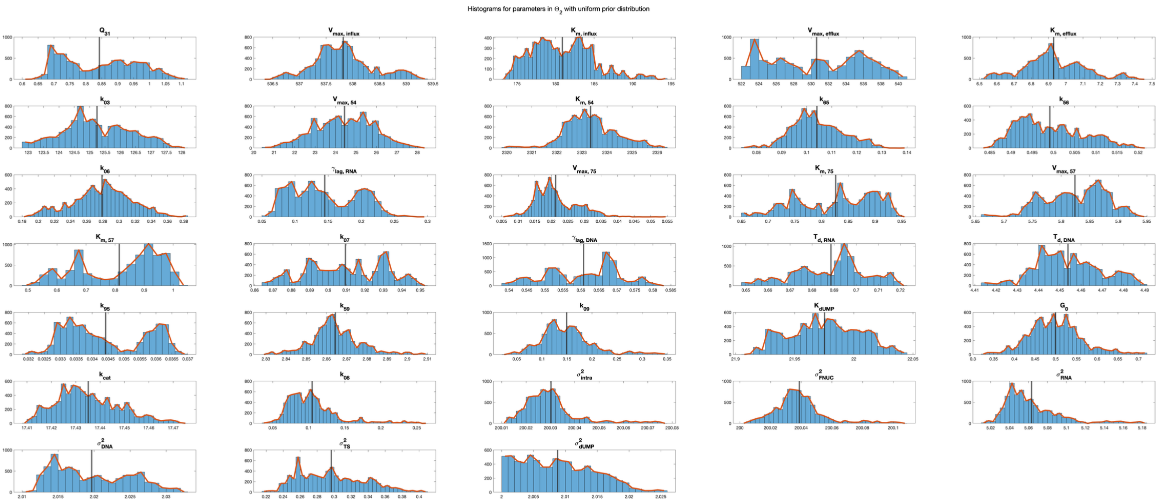

Fig N. Histograms of the parameters defined for model variables A_3(5-FU in interstitial fluid) to A_9(dUMP) in the cellular model under uniform prior distribution. The grey vertical line represents the mean parameter value reported in Table C. The parameters apart from Q_{31}, $K_{m,influx}$, $V_{m,efflux}$, $\gamma_{lag,RNA}$, $V_{max,75}$,$K_{m,57}$ k_{07}, k_{95}, and G_0 are identifiable. The variances of the measurement noises except for σ^2_{DNA} and σ^2_{TS} are identifiable.

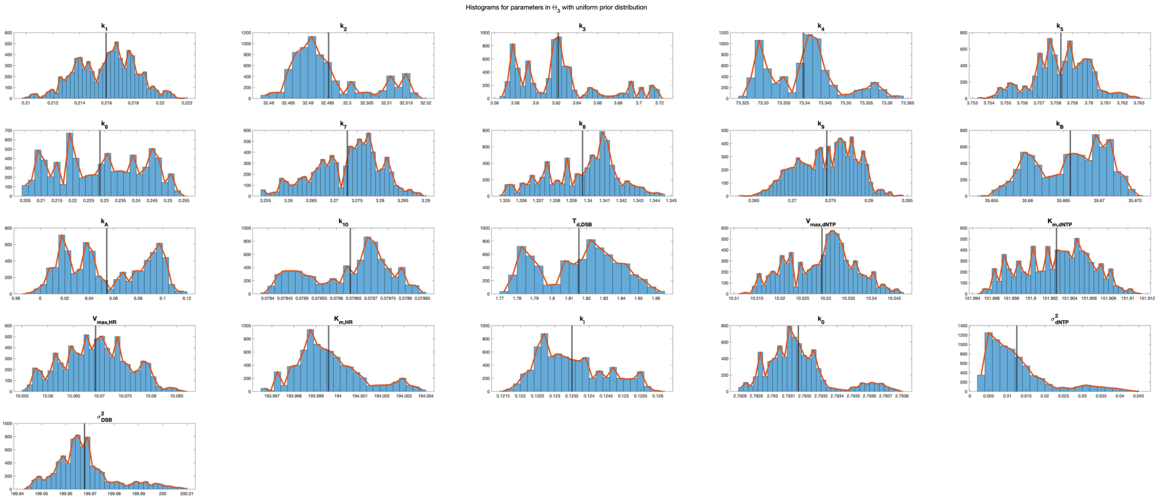

Fig O. Histograms of the parameters defined for model variables N_{dNTP} and N_{DSB} in the cellular model under uniform prior distribution. The grey vertical line represents the mean parameter value reported in Table C. The parameters apart from k_3, k_4,k_5,k_6,k_9, k_B, k_A, k_{10}, $T_{d,DSB}$, $K_{m,dNTP}$, and k_i are identifiable.

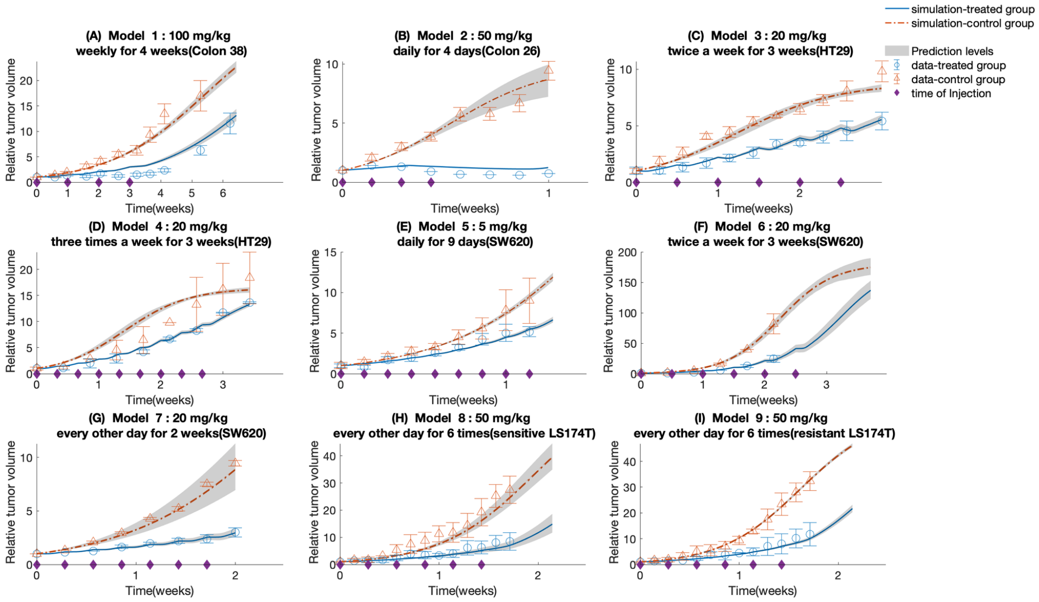

Fig P. Time-dependent outputs of the nine TGI model using parameter estimates shown in Table D. Red line represents the time course of the control group; blue line represents the time course of colon tumor growth treated with corresponding dosage regimens; triangle, literature data for the control group; circle, literature data for the treated group; error bar, standard errors; asterisk on x-axis represents injection time. The shaded areas in each panel represent the 0.7 credible intervals.

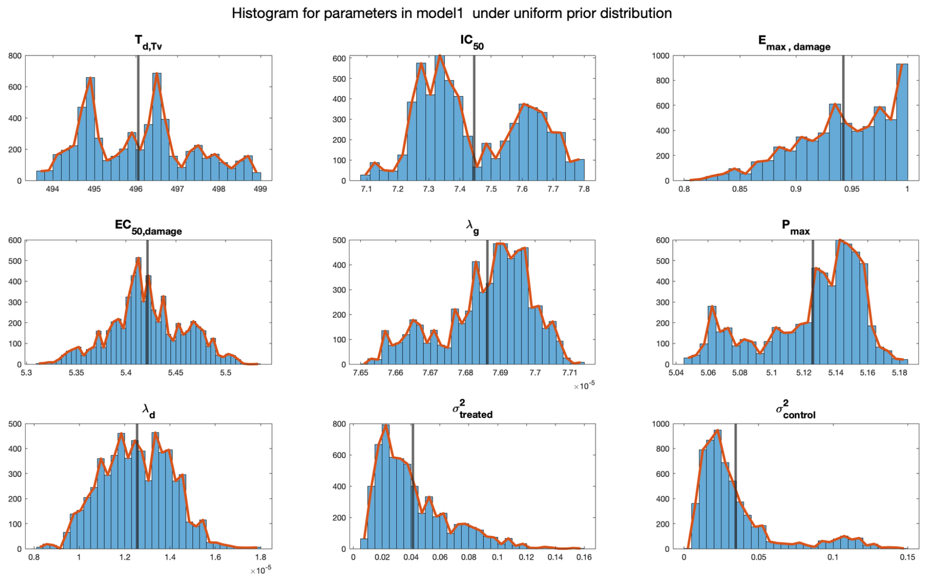

Fig Q. Histograms of the parameters defined for TGI model 1 under uniform prior distribution under uniform prior distribution. The grey vertical line represents the mean parameter value reported in Table D. As shown in the estimated posterior distributions of TGI model parameters, all the parameters apart from $T_{d,Tv}, IC_{50}$, and $E_{max,damage}$ are identifiable.

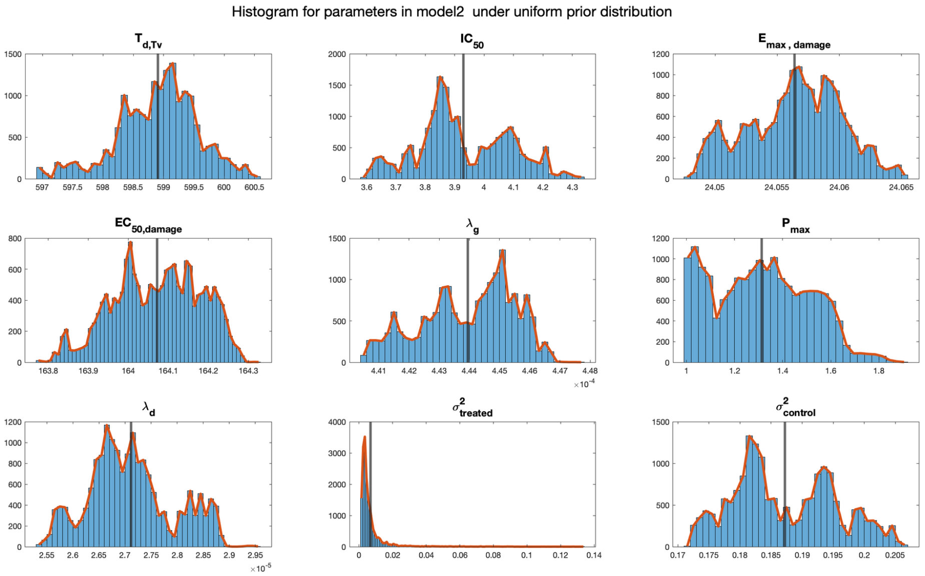

Fig R. Histograms of the parameters defined for TGI model 2 under uniform prior distribution. The grey vertical line represents the mean parameter value reported in Table D. In this case, P_{max} and $\sigma^2_{control}$ are non-identifiable

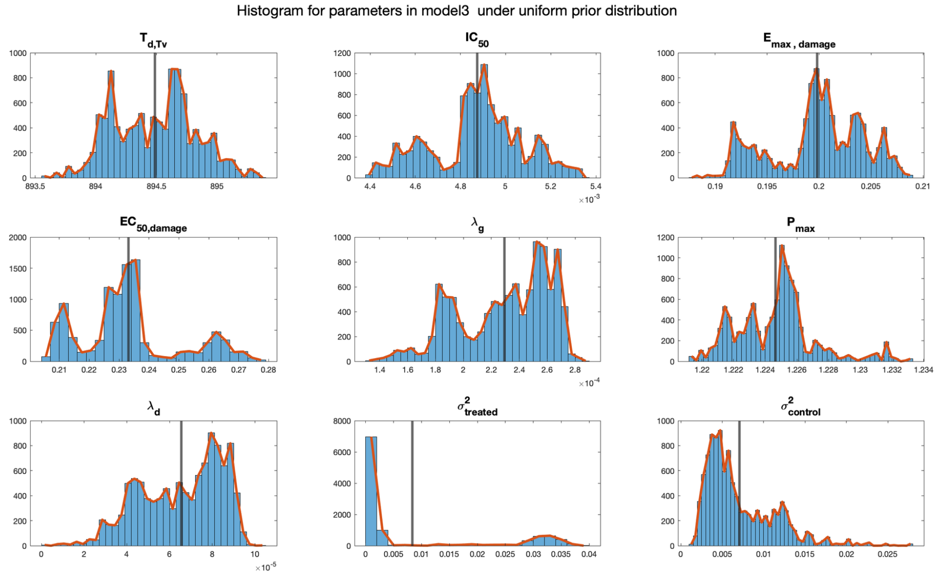

Fig S. Histograms of the parameters defined for TGI model 3 under uniform prior distribution. The grey vertical line represents the mean parameter value reported in Table D. It can be seen that the estimated distributions of $T_{d,Tv}$, λ_d, λ_g, and $P_{\max}$ are non-identifiable.

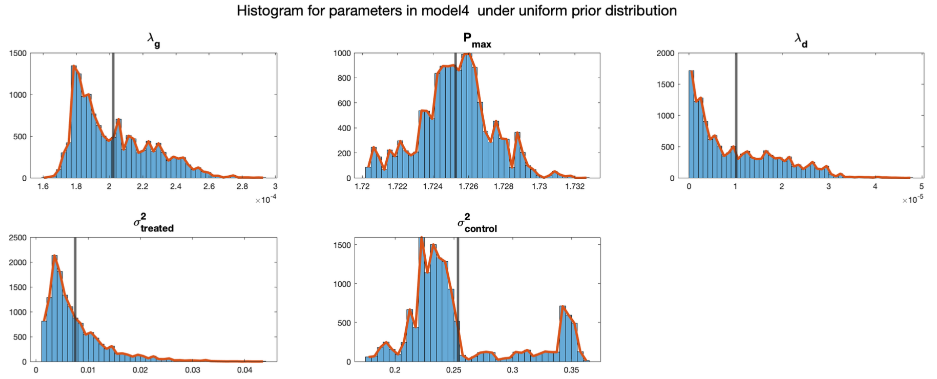

Fig T. Histograms of the parameters defined for TGI model 4 under uniform prior distribution. The grey vertical line represents the mean parameter value reported in Table D. The figure shows that all the parameters in TGI model 2 except $\sigma^2_{control}$ are identifiable.

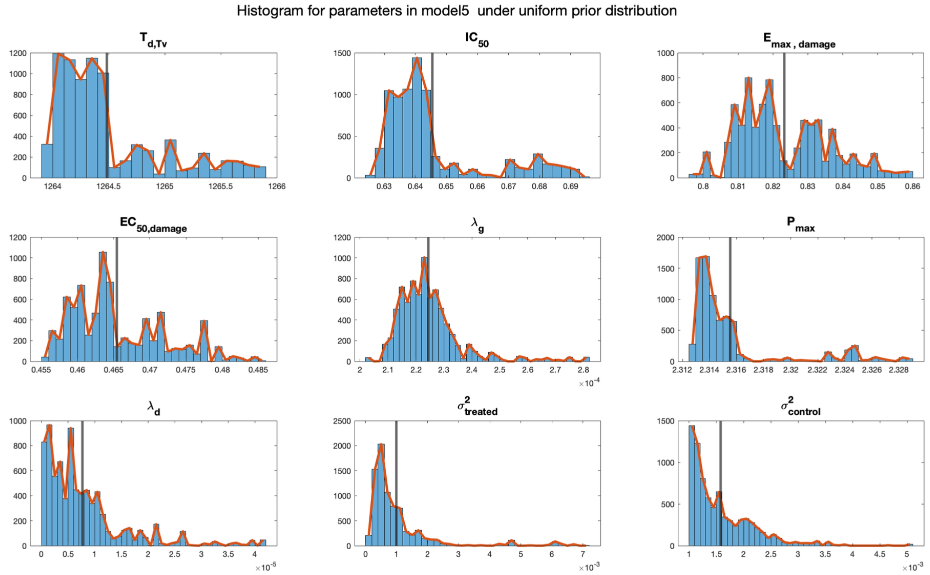

Fig U. Histograms of the parameters defined for TGI model 5 under uniform prior distribution. The grey vertical line represents the mean parameter value reported in Table D. In this case, the estimated distribution of all the parameters apart from $EC_{50,damage}$ and $EC_{max,damage}$ are identifiable.

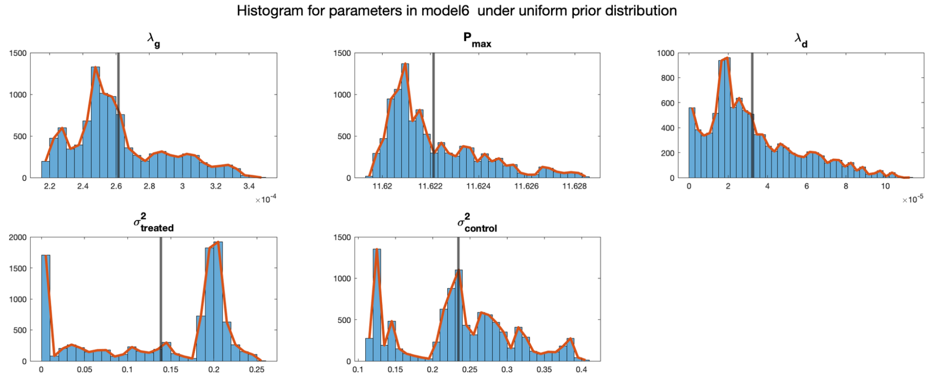

Fig V. Histograms of the parameters defined for TGI model 6 under uniform prior distribution. The grey vertical line represents the mean parameter value reported in Table D. In this case, $\sigma^2_{control}$ and $\sigma^2_{treated}$ are not identifiable.

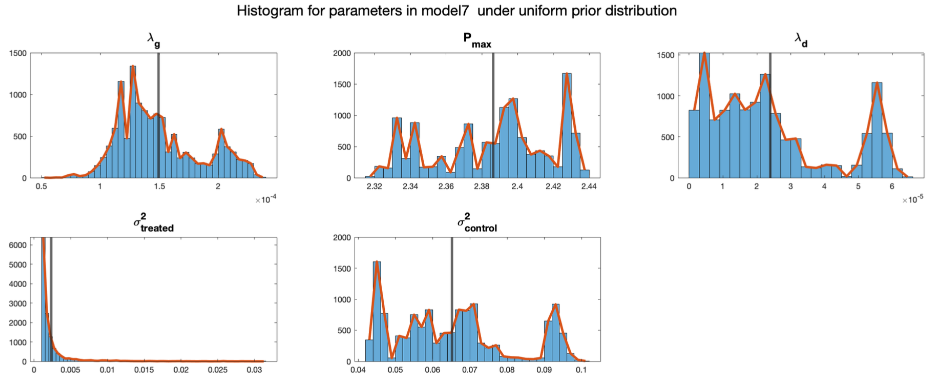

Fig W. Histograms of the parameters defined for TGI model 7 under uniform prior distribution. The grey vertical line represents the mean parameter value reported in Table D. In this case, P_{max}, λ_g, and $\sigma^2_{treated}$ are not identifiable.

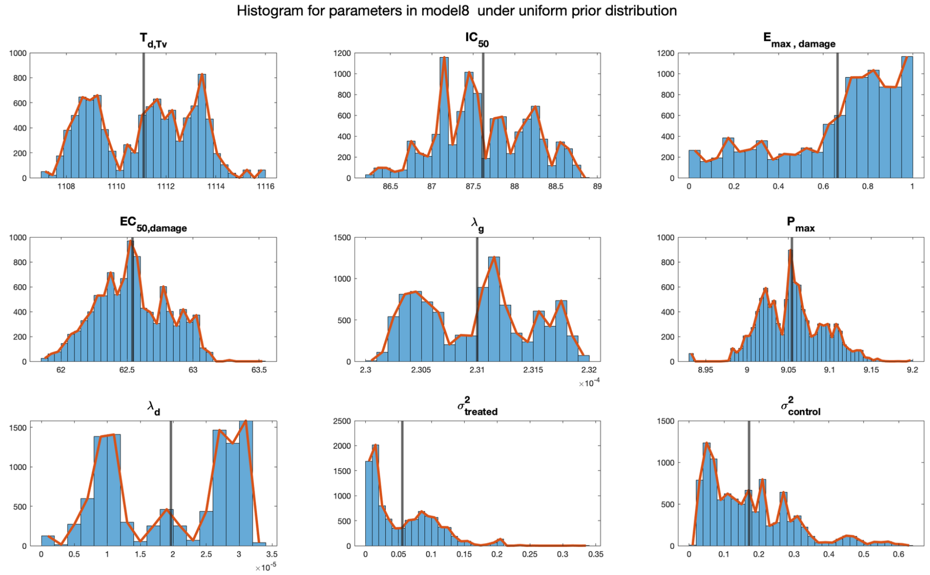

Fig X. Histograms of the parameters defined for TGI model 8 under uniform prior distribution. The grey vertical line represents the mean parameter value reported in Table D. The figure shows that the estimated distributions of $T_{d,Tv}$, IC_{50}, λ_d, and λ_g are not identifiable.

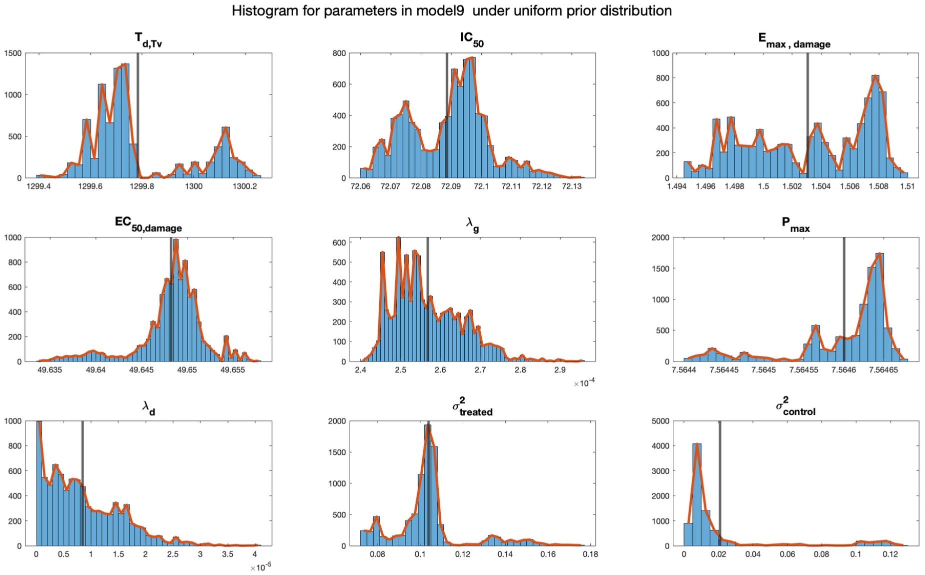

Fig Y. Histograms of the parameters defined for TGI model 9 under uniform prior distribution. The grey vertical line represents the mean parameter value reported in Table D. The figure shows that the estimated distributions of $T_{d,Tv}$, IC_{50}, λ_g, and $E_{\max,damage}$ are not identifiable.
